# Mechanochemical Diastereoselective Substrate-Directed Synthesis of Pyrrole-Isoxazolidines via 1,3-Dipolar Cycloaddition of Maleimides with Nitrones

**DOI:** 10.1021/acs.joc.6c00411

**Published:** 2026-07-27

**Authors:** Iva S. de Jesus, Anna Flávia da C. Lédo, Armando Ferrer, Mauro A. Bueno, Silvio Cunha

**Affiliations:** † Instituto de Química, 28111 Universidade Federal da Bahia, Campus de Ondina, Salvador, Bahia 40170-115, Brazil; ‡ 423876 Universidade Federal do Oeste da Bahia, Rua da Prainha, Morada Nobre, Barreiras, Bahia CEP 47810-059, Brazil

## Abstract

An efficient and sustainable substrate-directed mechanochemical synthesis of pyrrole-isoxazolidines was developed via the 1,3-dipolar cycloaddition of maleimides with nitrones. Moderate to good yields were generally obtained, and the diastereoselectivity is influenced by the steric (to *N*-alkyl nitrones) and electronic (EDG or electron-withdrawing group on aryl groups) nature of the substituents and, in some cases, is the opposite of that obtained by solvent-dependent methods. A reinterpretation of the known exo/endo transition states could explain the *cis*/*trans* selectivity, and on the basis of this, empirical guidelines are presented for predicting the diastereoselectivity as a function of substituents in this cycloaddition. Furthermore, this mechanochemical approach provided a protocol with short reaction times and simple processing, eliminating the need for column chromatography purification. Scaling up to one product and detailed green metrics to all products indicated that this mechanochemical approach is a greener route in relation to the traditional solvent methods.

## Graphical abstract

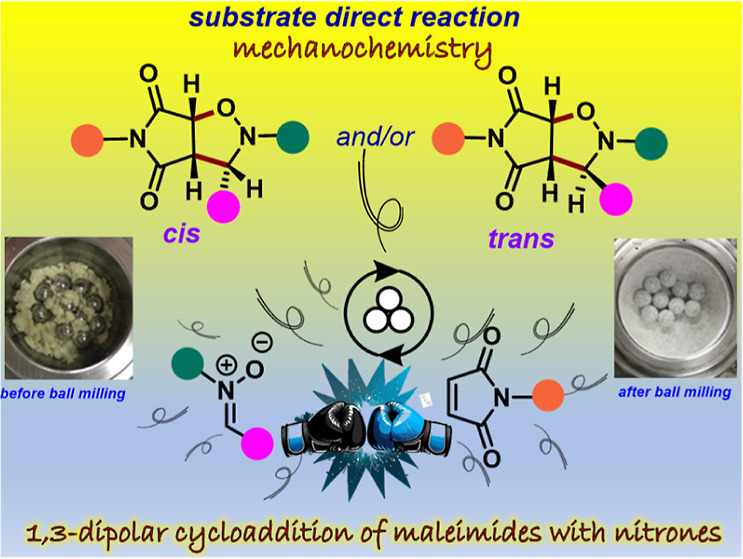

## Introduction

The saturated five-membered isoxazolidine ring is a privileged scaffold present in various natural products and bioactive compounds, Figure 1.^1^ This heterocycle displays a broad spectrum of biological activities,^2^ including antifungal,^3^ anti-inflammatory,^4^ antiviral,^5^ antibacterial,^6^ and antituberculosis.^7^ Isoxazolidines also hold promise as key intermediates in the synthesis of pharmaceutically relevant structures such as β-lactams, γ-amino alcohols, and β-amino acids.^8^ As a result, considerable efforts have been dedicated to exploring and developing isoxazolidine-based frameworks.^9^

**Figure 1:**
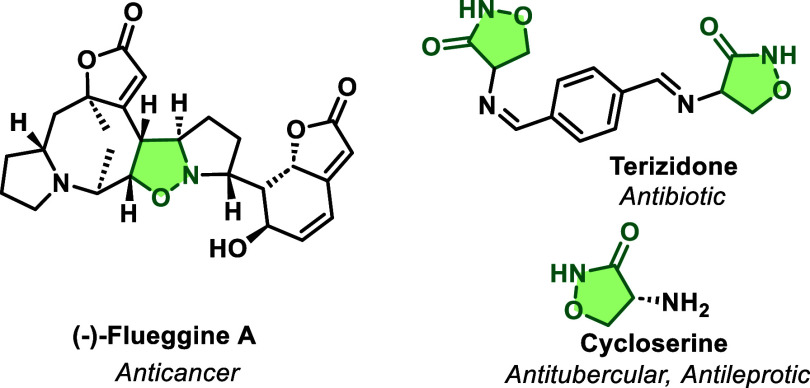
Drugs containing isoxazolidine ring systems.

Among the established strategies for the synthesis of isoxazolidine derivatives, the 1,3-dipolar cycloaddition reaction (1,3-DC) between nitrones and alkenes represents a straightforward way for accessing this class of compounds, enabling the formation of up to three contiguous stereocenters in a single step (Scheme 1a).^10^ When maleimides are used as dipolarophiles, N-substituted pyrrole-isoxazolidines are obtained, containing a pyrrole ring joined to an isoxazole moiety.^11^

**Scheme 1:**
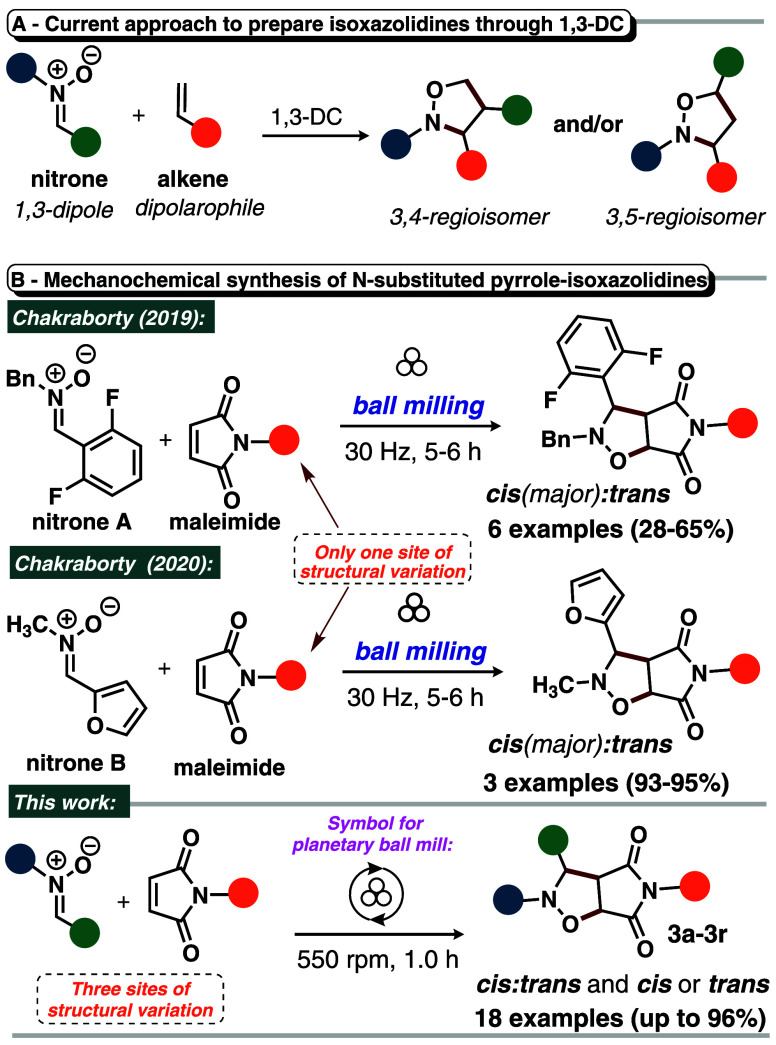
A) Formation of 3,4- and 3,5-Isoxazolidines through 1,3-Dipolar Cycloaddition (1,3-DC); (B) Mechanochemical Synthesis of *N*-Substituted Pyrrole-isoxazolidines via 1,3-DC

Despite substantial methodological advancements in this important class of cycloadditions, achieving high levels of regio- and stereoselectivity remains a persistent challenge.^12–14^ Moreover, the majority of existing protocols for synthesizing N-substituted pyrrole-isoxazolidines often rely on harsh experimental conditions, such as high temperatures and pressures, long reaction times, low-yielding products, and the employment of toxic organic solvents.^15–18^

Recently, mechanochemical synthesis has emerged as a viable and sustainable alternative to conventional solution-based processes.^19–32^ These approaches effectively avoid the need for solvents and elevated temperatures by harnessing the mechanical energy generated by impact forces within the reaction vessel. In this context, the application of mechanochemistry to intramolecular 1,3-DC^33a,33b^ and intermolecular variation for the synthesis of N-substituted pyrrole-isoxazolidines has recently been demonstrated by Chakraborty, showing a significant enhancement in reaction kinetics compared to conventional methods in solution.^33c,33d^ However, the substrate scope studied is very limited (only two nitrones end three maleimides) with no substituent variation at the phenyl groups in both 1,3-DC reaction components and modest diastereoselectivity, Scheme 1b. In light of these considerations, a more comprehensive investigation into this reaction, focused on the systematic evaluation of key parameters in mechanochemical synthesis, is highly warranted.

Regarding our ongoing research on mechanochemically based sustainable organic synthesis,^34^ herein we describe the mechanochemical synthesis of a series of structurally diverse N-substituted pyrrole-isoxazolidines via 1,3-dipolar cycloaddition reactions between nitrones and *N*-aryl maleimides. A range of reaction parameters were systematically evaluated, including the influence of auxiliary grinding liquids (LAG) on both the diastereoselectivity and the overall yield of the cycloadducts, considering the electronic influence of the substituent in each 1,3-DC reactant on yield and selectivity. Therefore, the first mechanochemically diastereoselective substrate-directed 1,3-dipolar cycloaddition of maleimides with nitrones was achieved.

## Results and Discussion

To understand the electronic influence of the substituent on the yield and diastereoselectivity of 1,3-DC between nitrone and maleimide, we considered the presence of electron-donating groups (EDGs) and electron-withdrawing groups (EWGs) in each reactant. To this end, a reaction was rationalized with aryl-substituted nitrone **1a** and maleimide **2a** containing *p*-CH_3_OPh and *p*-ClPh, respectively, as complementary EDG and EWG groups. With these model substrates, some parameters on the reaction outcome were evaluated, Table 1.

**Table 1:**
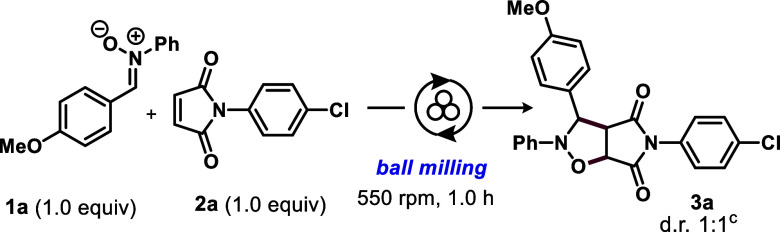
Optimization of Mechanochemical Reaction Conditions and Control Studies^c^

entry^a^	deviation from standard condition	yield (%)^b^
1	standard	95
2	5 stainless steel grinding balls of 5 mm	79
3	2 stainless steel grinding balls of 10 mm	50
4	4 stainless steel grinding balls of 10 mm	88
5	Without grinding balls	n.d^d^

a Standard condition: reaction carried out with 1**a** (0.25 mmol), **2a** (0.25 mmol), using 10 stainless steel grinding balls (5 mm diameter) in a 12 mL stainless steel vial.

b Isolated yield.

c Compounds were obtained as a mixture of 1:1 d.e. determined by ^1^H NMR.

d n.d., not detected.

After a set of preliminary experiments, we found that the reaction of 1.0 equiv of **1a** and 1.0 equiv of **2a** afforded the desired product **3a** in 95% yield after 1 h of grinding at 550 rpm (Table 1, entry 1), using 10 stainless steel balls (5 mm). Once this initial rotation value afforded **3a** in excellent yield, it was fixed to investigate the reaction scope under other mechanochemical parameters. Entries 2–5 summarize some deviations from these optimized conditions. Slight erosion in reaction efficiency was further observed when a smaller amount of grinding balls was used (Table 1, entry 2). When the reaction was conducted in the presence of 10 mm grinding balls, **3a** was not obtained (Table 1, entries 3–4); nitrone **1a** was detected, and maleimide **2a** was consumed, as indicated by thin-layer chromatography (TLC). On the basis of the results, we assume that the energy transferred to the reaction medium by the 10 mm grinding balls degrades the maleimide before the reaction is complete. Lastly, control experiments revealed that grinding balls were essential to the reaction outcome (entry 5). In addition, when reactants **1a** and **2a** were ground in a mortar-pestle, no reaction occurred.

With optimal mechanochemical reaction conditions established, we focused our attention on the scope of 1,3-DC. To this end, 1-aryl, *N-*phenylnitrones **1a**–**c** and *N-*aryl maleimides **2a**–**d** bearing EDG and EWG in each 1,3-DC reactant were evaluated, Scheme 2.

**Scheme 2:**
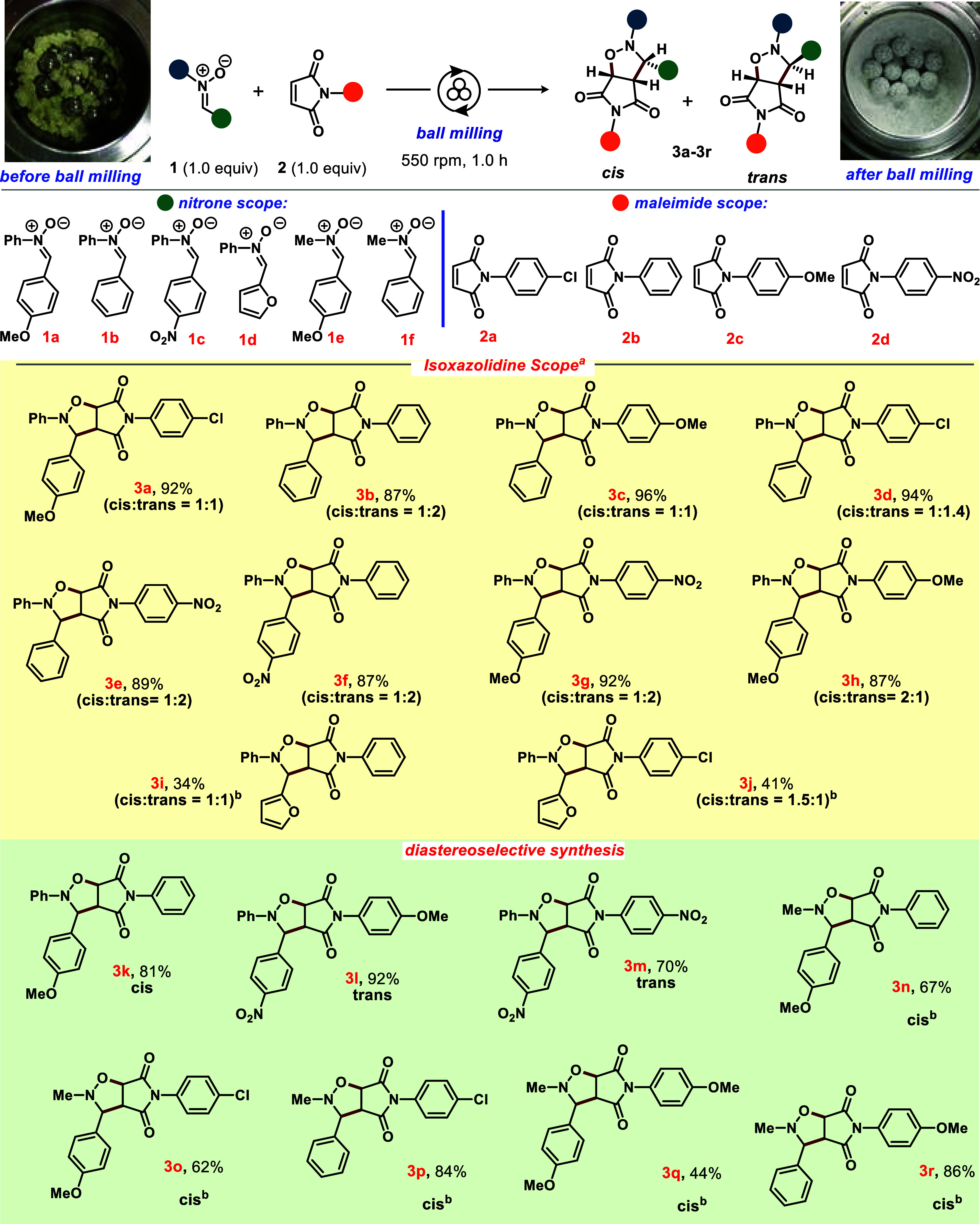
Mechanochemical Synthesis of N-Substituted Pyrrole-isoxazolidines via 1,3-DC Reaction^a^

a Nitrone (0.25 Mmol), maleimide (1.0 Equiv, 0.25 mmol), and 10 stainless steel grinding balls (5 mm). Yields refer to isolated compounds. The dr. Values were calculated from crude ^1^H NMR.
b Silica (100 Mg) was used as solid grinding auxiliary.

In relation to reaction yields, the obtained results showed good tolerance to the presence of different functional groups affording the corresponding N-substituted pyrrole-isoxazolidine derivatives **3a**–**3r**, Scheme 2. In general, the reaction of 1-aryl, *N-*phenylnitrones **1a**–**c** with *N-*aryl maleimides **2a**–**c** bearing EDG or EWG in these reactants underwent the desired transformation, giving products **3a-h,3k**–**m** in good yields. The protocol is also tolerated with heterocyclic scaffolds such as furan, widely explored in medicinal chemistry,^33b^ furnishing the corresponding products **3i** and **3j**, albeit in modest yields.

The reaction scope was extended to 1-phenyl, *N*-methyl nitrones **1e,f** evaluated with maleimides **2a**–**c**, delivering the corresponding N-substituted pyrrole-isoxazolidines **3n**–**r** in satisfactory yields.

When nitrones with a melting point below 100 °C were mechanochemically reacted, the reaction mixture forms a paste (see Figure S1), reducing the efficiency of collisions during the milling process^35^ and consequently lowering product yields. Although the reaction takes place at room temperature, the energy generated within the reactor increases the temperature, causing the nitrones to melt. In addition, paste formation reduces the overall efficiency of mechanochemical reactions, hindering the recovery of crude product from reaction vessels. This drawback can be mitigated by incorporating a grinding auxiliary to facilitate the formation of homogeneous solid dispersion, thereby improving product recovery and handling.^36^ To overcome this limitation, silica was added as a solid grinding auxiliary, allowing the reaction to proceed efficiently, affording **3i,j** and **3n**–**r**.

Gratified by the successful reaction scope and obtained yields under practical mechanochemical conditions, which afforded a representative set of cycloadducts, we turned our attention to the stereochemical aspect.

The reaction resulted in the formation of three continuous asymmetric centers in a single step. The ^1^H NMR analysis of the crude products revealed that **3a**–**j** had formed as a mixture of two diastereomers, while **3k**–**r** had formed only one. To the mixtures, the ratio was determined by analyzing the signals corresponding to the hydrogen H4 of the isoxazolidine moiety in both isomeric cycloadducts. For **3j**, the two diastereomers were separated by column chromatography and identified by NMR, analyzing the multiplicity and coupling constant values of the hydrogen signals at H3, H4, and H5, highlighted in Figure 2.^37^ In the *cis* diastereomer of **3j** (Figure 2, left), H4 is observed as a double doublet with coupling constants of 9.0 and 8.0 Hz, whereas H3 and H5 are doublets with coupling constants 9.0 and 8.0 Hz, respectively. These values indicate a *cis* relationship between H5 and H4 in a five-membered ring. Conversely, for the *trans* diastereomer of **3j** (Figure 2, right), H4 is observed as a large doublet with coupling constants 7.5 Hz, whereas H3 is a large singlet (a very small coupling constant indicating a *trans* relationship between H4/H3), and H5 is a doublet of 7.5 Hz.

**Figure 2:**
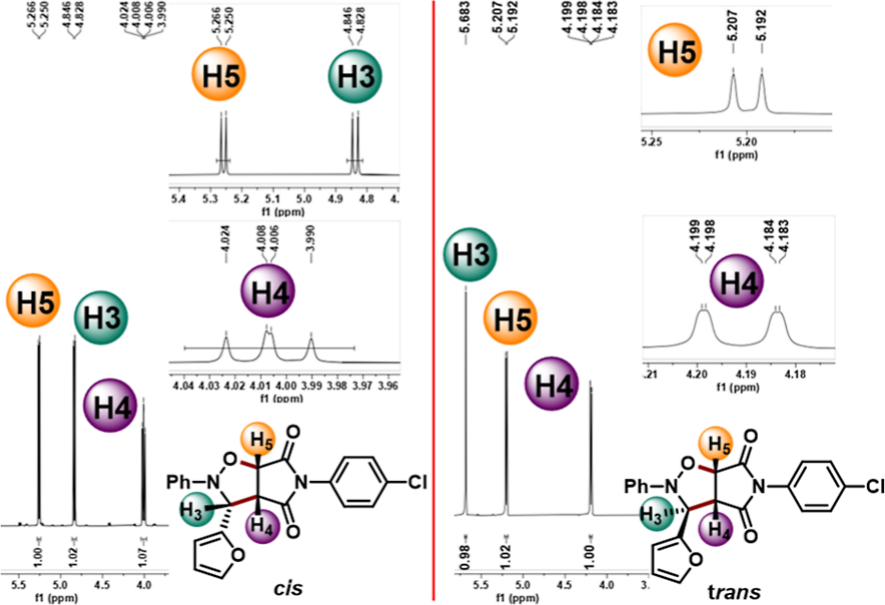
Comparison of partial ^1^H NMR spectra (CDCl_3_, 500 MHz) of diastereomeric **3j** *cis* (left) and *trans* (right) showing hydrogens H3/H4/H5 of the isoxazolidine moiety.

The results depicted in Scheme 2 represent the broader set of pyrrole-isoxazolidines synthesized by mechanochemical 1,3-dipolar cycloaddition of maleimides with nitrones. In this context, we observed a diastereoselective substrate-directed effect, whose rationalization will be presented. Before this, it was now possible to compare ball-milling technology with other methodologies that used solvents.^38^

Advantages were found in some cases, such as better yields and diastereoselectivity of cycloadducts in relation to the corresponding solvent-dependent synthesis. Under conventional heating using toluene as a solvent, for example, the reactions occurred slowly (up to 10 h versus 1 h mechanochemically) and at high temperature (110 °C).^5^ Furthermore, column chromatography was necessary to purify products, while cycloadducts were herein obtained in shorter reaction times and purified by simple recrystallization. On the other hand, the cycloaddition reaction between nitrones and maleimides under photoredox catalysis exhibits a diastereoselectivity distinct from that observed under mechanochemical conditions. For instance, in the case of cycloadduct **3b**, only the *cis* diastereomer is formed via photoredox catalysis.^39^ In contrast, the mechanochemical approach yields a diastereomeric mixture with a predominance of the *trans* diastereomer.

After reliable differentiation of each *cis*-/*trans*-diastereomer, the observed selectivity was rationalized. This task proved to be somewhat complex due to the apparent random distribution, as shown in Scheme 2. Some nitrone/maleimide combinations produce a mixture of *cis*/*trans* isomers, sometimes one predominating and sometimes the other, while other combinations produce only the *cis* or *trans* isomer. This scenario contrasts with the other two notable studies developed by Chakraborty^33c,33d^ that describe the mechanochemical reaction of 1,3-DC between nitrone and maleimide, whereby a mixture of *cis/trans* isomers (*cis* being the major isomer) was formed, Scheme 1.

To reinforce our hypothesis that the electronic nature of the substituent directly influences diastereoselectivity, we proceeded to a Hammett analysis of the cycloaddition reactions. This Hammett analysis provides insight into the electronic effects of the substituent on the reagent, specifically regarding the transition-state energy during product formation.^40^ The individual analysis of the diastereomeric ratio of the products obtained from the reaction between **2d** with **1a**–**c** reveals that the formation of the *trans* diastereomer increases proportionally to the substituent effect, as indicated by the relationship between the Hammett constant for each substituent (σ R1) and the ratio between the amounts of *trans* and *cis* diastereomers Dt/Dc, expressed as a percentage, Figure 3. Although the Hammett constants and *trans/cis* ratio is not a simple relationship, it is observed that as the electronegativity of R1 increases, the log D*t*/D*c* values also increase, confirming that the *cis*/*trans* selectivity in these 1,3-DC is directly influenced by the electronic properties of the substituent in the nitrone structure. A plot of the Hammett substituent constants (σ_p_) at the *para*-position of the nitrone versus the percentage of *trans*-diastereomers highlights several key trends, Figure 3. Highly EWGs, such as the nitro substituent, afford nearly exclusive *trans*-selectivity (**3** and **3m**) regardless of the maleimide partner. Conversely, EDGs like (–OCH_3_) shift the selectivity toward the *cis*-diastereomer (**3k**, **3n**, **3o**, **3q**, and **3r**). Other substituent combinations (**3a**, **3b**, **3c**, **3d**, **3e**, **3f**, **3g**, and **3h**) demonstrate a clear trend where increasing polarity favors the *trans*-isomer. Given the absence of specific Hammett-type parameters for maleimides in the literature, a direct mathematical correlation remains elusive; however, it is evident that diastereoselectivity is primarily governed by the electronic nature of the nitrone, with the *N*-phenylmaleimide effect being secondary. Further studies are required to decouple the relative contributions of electronic and steric factors to this observed selectivity.

**Figure 3:**
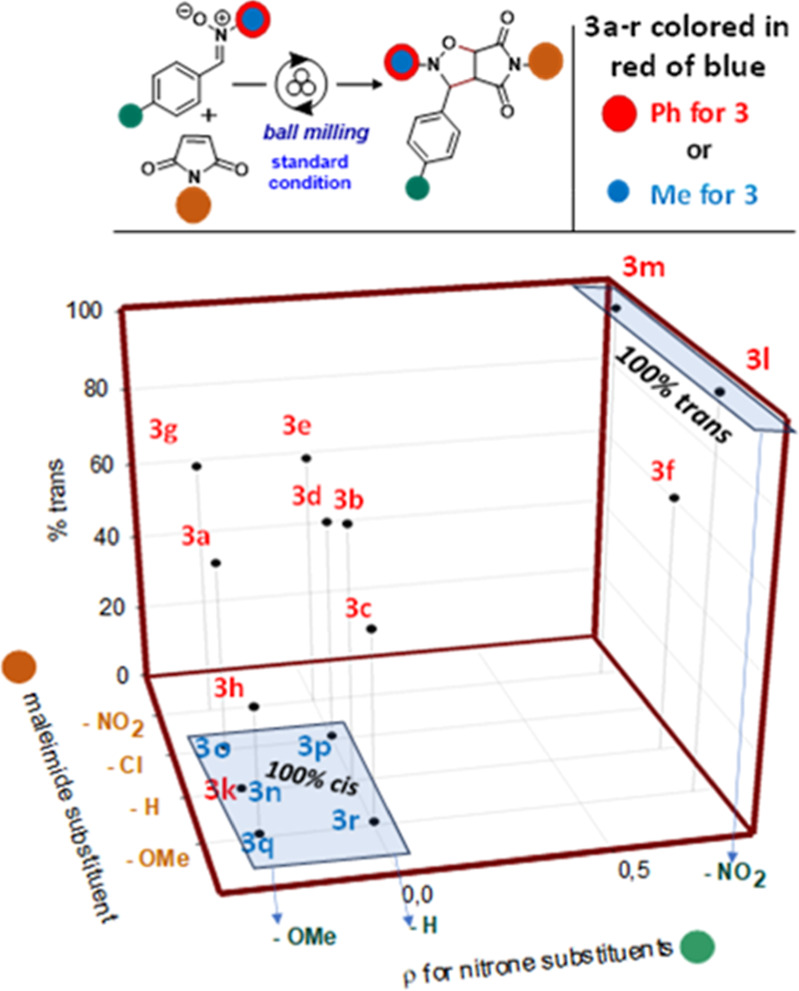
Comparison between the diastereomeric ratio of the pyrrole-isoxazolidine obtained by 1,3-DC via mechanochemistry and Hammett constant of substituent of nitrone (σ R1) and maleimide substituent versus percentage (%) of diastereomer *trans*.

Computational analyses have been reported to rationalize the mechanism and diastereoselectivity,^41^ invoking various electronic and energetic factors to explain the predominant *cis* isomer in the previously described mechanochemical 1,3-DC.^33c,33d^ However, these described parameters in the reported theoretical studies are insufficient to explain the influence of the substituent (EDG or EWG) on the selectivity observed in all examples of Scheme 2, since in some cases the *trans*-isomer is the major or exclusive one.

Following an exhaustive analysis of available computational works,^41^ we propose an additional explanation based on the structures of transition states (TS) calculated in these studies, which can explain the trends observed in Scheme 2, considering the electronic influence of the substitute (EDG or EWG) on the observed selectivity. Mechanistically, two approximations are possible, exo and endo, which result in *cis* and *trans* diastereomers, respectively, Figure 4A. On the one hand, in the optimized exo TS, the C1-aryl nitrone group eclipses with one of the C=O carbonyls of the maleimide, developing the noncovalent π_aryl_/π_C=O_ stacking interaction. On the other hand, when the endo TS is optimized, the *N*-aryl nitrone group eclipses with the *N*-aryl maleimide moiety, developing π_aryl_/π_aryl_ stacking (these π–π interactions are highlighted in yellow, Figure 4A). Following an exhaustive analysis of available computational works,^41^ we propose an additional explanation based on the structures of calculated TS in these studies, which can explain the trends observed in Scheme 2, considering the electronic influence of the substitute (EDG or EWG) on the observed selectivity. Mechanistically, two approximations are possible, exo and endo, which result in *cis* and *trans* diastereomers, respectively, Figure 4A. On the one hand, in the optimized exo TS, the C1-aryl nitrone group eclipses with one of the C=O carbonyls of the maleimide, developing the noncovalent π_aryl_/π_C=O_ stacking interaction. On the other hand, when the endo TS is optimized, the *N*-aryl nitrone group eclipses with the *N*-aryl maleimide moiety, developing π_aryl_/π_aryl_ stacking (these π–π interactions are highlighted in yellow, Figure 4A).

**Figure 4:**
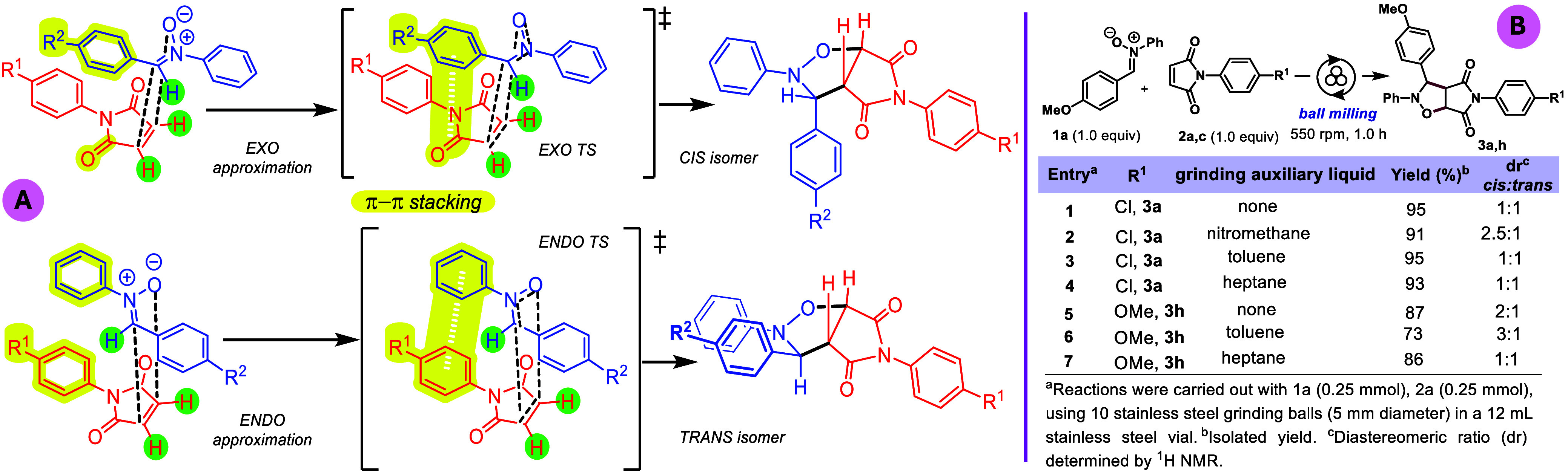
(A) Exo/endo approximations and TS with π–π stacking interactions highlighted in yellow (π_aryl_/π_C=O_ to exo TS and π_aryl_/π_aryl_ to endo TS) to 1,3-DC of *Z*-nitrone with maleimides affording diastereoisomeric bicyclic isoxazolidines and (B) influence of LAG on yield and diastereomeric ratio (dr).

The diastereoselective synthesis of Scheme 2 can be directly rationalized by the π–π stacking shown in Figure 4A. Thus, in the exo TS to **3k**, the C=O group is a strong π electron acceptor, and p­(CH_3_O)­Ph is a strong π electron donor, whereas in the endo TS, the two unsubstituted Ph are weak π electron acceptors/donors. In this way, the *cis* selectivity is due to the strong π_*p*(MeO)Ph_/π_C=O_ interaction, in contrast to the weak π_Ph_/π_Ph_ stacking. If the aryl component of the π_aryl_/π_C=O_ interaction becomes a poor π donor, as in the case of **3L** and **3m** with the strong EWG p­(NO_2_)­Ph in C1-nitrone, the π_Ph_/π_Ph_ stacking becomes preferential and the endo TS is favored even against *p*-EWG or *p*-EDG in *N*-arylmaleimide. Furthermore, when *N*-methyl nitrone **1e,f** reacted to produce **3o**–**r**, only *cis* cycloadducts were formed. To **3o**–**s**, the *N*-alkyl nitrone hinders (or prevents) the concurrence of endo TS, since there is no π_aryl_/π_aryl_ stacking to compete with the π_aryl_/π_C=O_ stacking of exo TS. This rationale also explains all products of Chakraborty’s studies.

As mentioned in the mixtures, there is an apparent random distribution of diastereomers in Scheme 2. However, the low or nonexistent selectivity observed for **3a**–**j** can be explained in the same way as the selective formation of **3k**–**r**. Rationalization can be achieved by fixing the nitrone and varying the maleimide, as for **3a,g,h**, which has in common the same π_aryl_/π_C=O_ stacking stabilization. For the **3a**, both exo and endo TS are favored because π_*p*(MeO)Ph_/π_C=O_ and π_Ph_/π_*p*ClPh_ interactions should be equally stabilizing, justifying the 1:1 isomeric ratio. Comparing **3a** and **3g**, endo TS becomes favored in **3g**, and the *trans* isomer increases due to the greater EWG power of the nitro group in the π_Ph_/π_*p*(NO2)Ph_ interaction, increasing the isomeric ratio to 1:2 (*cis/trans*). Similarly, **3h** has a strong *p*-(EDG)­Ph relative to **3a,g**, which should inhibit electron flow from the C1-Ph nitrone to the *N*-aryl maleimide group, not favoring the endo TS and decreasing the *trans* isomer, inverting the isomeric ratio to 2:1 (*cis/trans*).

The same rationalization can be achieved for **3b**–**e**, wherein the *N*-aryl maleimide with neutral PhH and *p*-(EWG)­Ph, endo TS becomes favored, and the *trans* isomer is favorable due to π_Ph_/π_*p*(EWG)Ph_ interactions in **3b**–**e**. Conversely, for **3c**, the endo TS becomes disfavored in relation to **3b** because *p*-(CH_3_O)­Ph decreases π_Ph_/π_*p*(EDG)Ph_ interactions and *trans* isomer decreases, and isomeric ratio changes from **3b** to **3c** (from 1:2 to 1:1/*cis/trans*, respectively), Scheme 2. The same line of reasoning can explain various combinations of Scheme 2, such as **3**d**/3j**, **3**e**/3f**, and **3**i**/3j**, for example. The rationalizations herein developed suggest a general trend to accommodate and predict the substrate-directed diastereoselectivity of mechanochemical 1,3-DC of maleimides with nitrones, Figure 5. These tentative guidelines to predict selectivity are deduced from the results of Scheme 2 and literature results^33c,33d^ and are based on the electronic and steric interactions of alkyl/aryl substituents of nitrone and maleimide. With these self-explanatory predictive guidelines, all known products of Schemes 1 and 2 can be rationalized, and new combinations of reactants with adequate substituents can be planned to accomplish good selectivity. Apparently, a proximal interaction between the indicated groups of Figure 5 is more relevant than a distal interaction in the deduction of which diastereomer will prevail.

**Figure 5:**
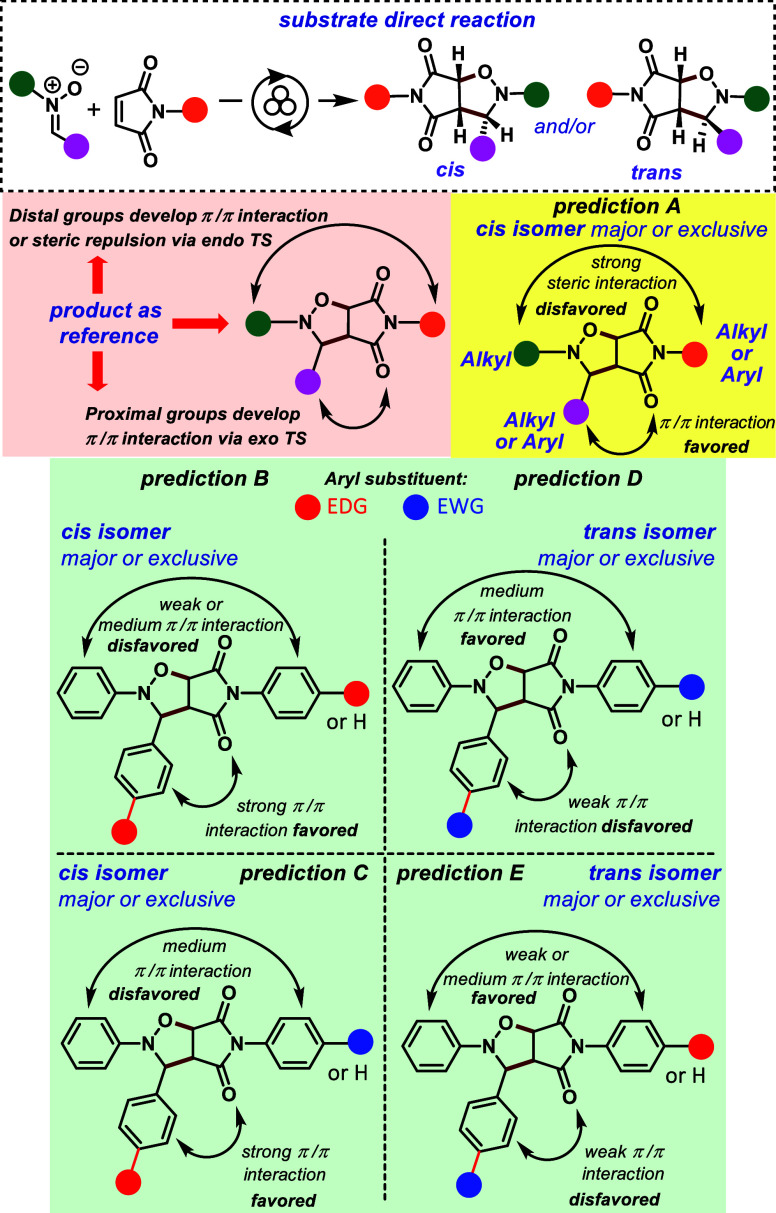
Practical guidelines to predict diastereoselectivity as a function of substituents in the mechanochemical substrate-directed 1,3-dipolar cycloaddition of maleimides with nitrones.

According to a previous experimental study, ethanol and acetonitrile do not change the reaction rate of known reactions of Scheme 1b,^33c,33d^ and theoretical studies have shown that inclusion of solvent effects of ethanol and acetonitrile increases the activation energy, disfavoring both exo and endo pathways, with endo TS being more affected.^41^ Considering that there is no study concerning the solvent effect in the selectivity of mechanochemical 1,3-DC of maleimides with nitrones, the effect of solvent was investigated, according to the proposed model of Figure 4A. Liquid-assisted grinding (LAG) can be investigated to enhance the mechanochemical process. These auxiliaries can play a crucial role in modulating reaction selectivity toward the desired products^42^ while also influencing reaction yields depending on their specific characteristics.^43^ For these reasons, we also investigated the influence of LAG on the yield and, more importantly, the diastereoselectivity of the cycloaddition reactions between nitrone **1a** and two maleimides **2a**,**c** with distinct reactivity patterns (R1 = Cl and R1 = OCH_3_ in Figure 4B and Scheme 2), affording **3a**,**h**.

Three liquids with different polarities (heptane, toluene, and nitromethane) were tested as LAG, and significant variations in reaction outcomes were observed depending on the nature of the auxiliary employed, see Table of Figure 4B. For **2a** with R1 = Cl, the use of a more polar milling liquid (entry 2) stabilized the exo transition state, leading to an increased formation of the *cis*-diastereomer. In contrast, less polar solvents (entries 3 and 4) exhibited no significant effect on the diastereoselectivity of the cycloaddition. On the other hand, when **2a** containing R1 = OCH_3_ was used, it was observed that the low-polar solvent (entry 6) favored the formation of the *cis*-diastereomer and an apolar solvent (entry 7) favored the formation of the *trans*-diastereomer. The mechanochemical reaction using nitromethane as LAG for the **3c** was performed; however, the ^1^H NMR spectrum was inconclusive.

In general, the use of LAG was not significantly detrimental to the yield (entries 5–7). The selectivity of these reactions does not follow a common pattern for the two nuclei **3a**,**h** evaluated; therefore, a more in-depth study should be carried out to better understand the influence of the substituent groups of the nitrones and maleimides on the TS of 1,3-DC when an LAG is used in mechanochemical synthesis. Apparently, the endo TS of **3a,h** is disfavored in more polar solvents because dipole orientations of two groups (R^1^ = CH_3_O and R^2^ = CH_3_O/Cl in Figure 4A) tend to minimize the polar effect when compared to the exo TS.

To evaluate the practical scalability of this mechanochemical protocol, the synthesis of isoxazolidine **3k** was investigated on an increased scale. Elevating the reaction scale from 0.25 to 1.0 mmol while maintaining a 1h milling time led to a pronounced decrease in yield from 81% to 42%. Notably, performing the reaction in the presence of silica as a solid auxiliary slightly enhanced the efficiency, affording **3k** in 50% yield. This finding highlights the capability of solid supports to modulate the reactivity in larger-scale mechanochemical processes. Regardless of the auxiliary used, these observations indicated that achieving an optimal conversion at larger scales requires extended milling times. Consequently, increasing the reaction time to 2 h restored the efficiency, providing **3k** in 79% yield, which suggests a direct correlation between the grinding time and product conversion. Finally, further scale-up to a 10 mmol scale was successfully performed; although it required an extended milling time of 16 h, it delivered the desired product in a commendable 65% yield.

Because mechanochemistry inherently targets solvent-free synthesis, a comprehensive evaluation of green chemistry metrics^47^ was conducted to quantify the sustainability of this protocol. In this system, solvents are completely omitted during the reaction and are employed exclusively during subsequent isolation and purification steps. Since the formation of isoxazolidines via 1,3-dipolar cycloaddition proceeds with 100% atom economy, more discriminating criteria such as mass intensity (MI) and the environmental factor (*E-*factor) were employed to rigorously assess the impact of minimizing solvent consumption, Figure 6. MI serves as an illustrative metric by directly relating the total mass of all inputs (excluding water) to the mass of the isolated product on a weight-by-weight basis, where an ideal process approaches a value of 1.

**Figure 6:**
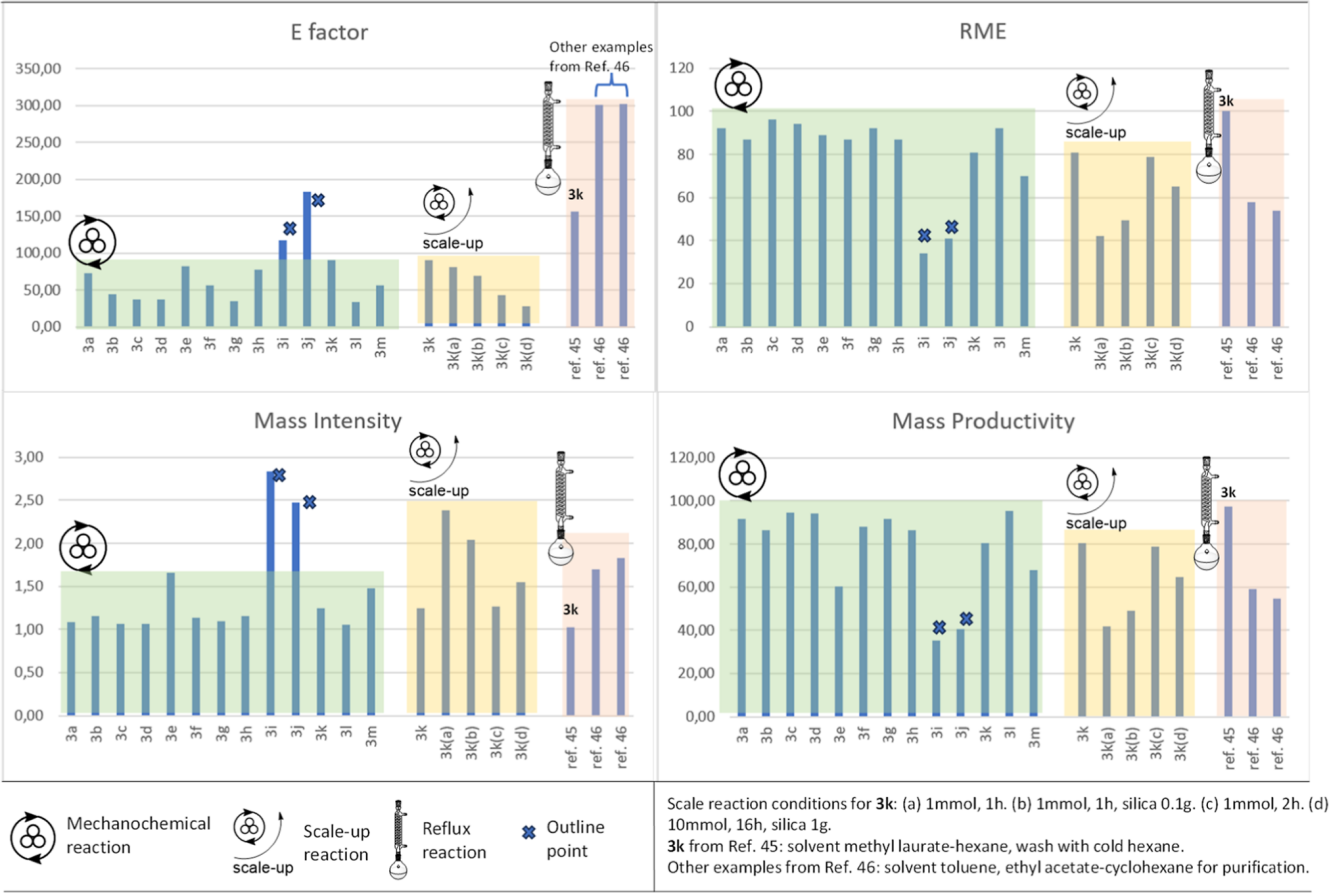
Comparison of green chemistry metrics for the mechanochemical synthesis of isoxazolidines (this work) versus conventional solvent-mediated reflux conditions (reported literature values).^45,46^

With the exception of the outliers identified for derivatives **3i** and **3j**, the MI and mass productivity metrics reveal a systematic convergence toward the ideal unitary value across the evaluated mechanochemical processes. The pronounced divergence observed for **3i** and **3j** directly correlates with the intrinsic reactivity of the furan core. Possessing a low resonance energy and diminished aromatic stability, the furan ring frequently behaves as a reactive conjugated diene rather than an inert aromatic system. Under the high-energy shear stress and impact conditions of the mechanochemical reactor, the carbon–oxygen bond undergoes mechanical activation that induces nucleophilic or electrophilic ring-opening pathways. These competing decomposition routes significantly lower the overall chemical yield, thus driving the environmental and mass metrics away from the ideal baseline. Crucially, the greenness of this protocol is further validated by the environmental factor (*E-*factor) profile. A dramatic disparity is observed when comparing our neat mechanochemical framework against traditional solution-phase methodologies reported in the literature,^45,46^ where extensive solvent requirements and arduous aqueous workup/purification protocols inflate the *E-*factor up to values near 300 for **3k** (see ref 45); other examples of 1,3-dipolar cycloaddition of maleimides with nitrones from ref 46 are presented in Figure 6. In stark contrast, our bench-scale millimanipulations maintain a highly compressed waste footprint. More importantly, upon scaling up the process to a 10 mmol regime indicated for **3k** in condition **3k**(d) of Figure 6, a continuous stepwise minimization of the *E-*factor is achieved. This confirms that the mechanical activation mechanism scales endergonically without requiring proportional increases in processing solvents or purification masses, solidifying the synthetic scalability of this methodology. Consequently, the comparison of environmental factors confirms a profound reduction in waste generation when contrasting this neat mechanochemical approach with classical, solvent-mediated reflux methods. In this framework, solvent utilization is exclusively restricted to the minimal volumes necessary for product isolation from the grinding matrix, thereby maintaining an exceptionally suppressed *E-*factor. Taken together, the holistic assessment of these parameters, complemented by the favorable scaling trends in reaction mass efficiency and mass productivity, unambiguously establishes this mechanochemical protocol as a premier, sustainable alternative to traditional solution-phase synthesis.

## Conclusions

This study demonstrated an efficient and sustainable mechanochemical substrate-directed synthesis of pyrrole-isoxazolidines via 1,3-dipolar cycloaddition of maleimides with nitrones, a still underexploited area. The mechanochemical methodology offers significant advantages over conventional solvent-based protocols, including shorter reaction times, milder conditions, and simplified product isolation without column chromatographic purification. Systematic investigations revealed that both the nature of the starting materials and the milling parameters, such as ball size and its number, and the use of grinding auxiliaries have a significant impact on yield and diastereoselectivity of the cycloadducts. Notably, the addition of silica as a solid grinding auxiliary proved essential for reactions involving low-melting nitrones, while the LAG method exhibited substrate-dependent effects on stereoselectivity. The diastereoselectivity is influenced by steric (to *N*-alkyl nitrones) and by electronic (to *N*-aryl nitrones and maleimides with *p*-EDG-Ph and *p*-EWG-Ph) nature of the substituent and, in some cases, is the opposite of that obtained by solvent-dependent methods. A reinterpretation of known exo/endo TS could explain *cis*/*trans* selectivity based on π_aryl_/π_C=O_ and π_aryl_/π_aryl_ stacking interaction, and based on this, empirical guidelines are formulated for predicting the selectivity as a function of substituents in the substrate-directed 1,3-dipolar cycloaddition of maleimides with nitrones. The results described herein represent a greener route and pave the way to develop a mechanochemical route to an asymmetric 1,3-dipolar cycloaddition reaction between nitrones and maleimides. This hypothesis is under investigation, and results will be described in due course.

## Experimental Section

### Materials and Methods

Melting points were determined on a Microquímica MQAPF 301 hot plate apparatus and are uncorrected. Infrared spectra were recorded as KBr discs on Shimadzu IR Affinity-1. The ^1^H NMR and ^13^C NMR of samples in CDCl_3_ and DMSO-*d*_6_ were measured using a Bruker Avance III 500 spectrometer, with tetramethylsilane as an internal standard for ^1^H and ^13^C. For ^1^H NMR spectra, chemical shifts were reported in ppm (δ), multiplicity (s = singlet, d = doublet, t = triplet, q = quartet, dd = double doublet, td = triple doublet, ddd = double doublet, and m = multiplet), and coupling constant (Hz). Mechanochemical reactions were performed in a planetary ball mill Retsch PM100. Elemental analyses were performed on a PerkinElmer 2401 Elemental Analysis. Chromatography columns were carried out using Merck 70–230 mesh silica gel. Analytical TLC was performed using Merck silica gel 60F_254_, 0.2 mm precoated TLC plates. TLC plates were visualized using UV (254 and 366 nm). High-resolution mass spectra (HRMS) were recorded on a Thermo Scientific Q-Exactive mass spectrometer equipped with a heated electrospray ionization source operating in both positive and negative ionization modes. Samples were prepared in HPLC-grade methanol at a concentration of 50 mg mL^–1^ and filtered through a 0.22 μm PTFE syringe filter prior to analysis. The capillary temperature was set at 320 °C and the auxiliary gas temperature at 37 °C. Mass spectra were acquired over the *m*/*z* range of 150–950, using a spray voltage of 4.0 kV and an S-lens RF level of 50. Sheath and auxiliary gas flow rates were set to 12 and 0 arbitrary units, respectively. Spectral data of compounds **3a**–**c**, **3e**,**f**, **3h**,**i**, **3k**, and **3o** are in accordance with the reported literature and are indicated in each case.

### General Procedure for the Mechanochemical Synthesis of N-Substituted Pyrrole-isoxazolidines

In the 12 mL stainless steel container of the ball mill, the nitrone, the maleimide, and 10 stainless steel spheres (5 mm diameter) were added. The reactor was placed in a planetary ball mill, and the parameters were adjusted to a rotation speed of 550 rpm, with directional inversion every 30 min. The reaction progress was monitored by TLC—thus, a small amount of the mixture was collected and dissolved in ethyl acetate (AcOEt). When necessary, silica (SiO_2_) was added as a milling auxiliary (indicated in each case). After grinding, the crude mixture was dissolved in ethyl acetate (AcOEt) and transferred to a 25 mL round-bottom flask, followed by concentration under reduced pressure. The crude product was then recrystallized from hexane/ethyl acetate to afford a white solid. In cases where silica (SiO_2_) was used as a milling auxiliary, the reaction mixture was dissolved in ethyl acetate (AcOEt) and transferred to a sintered-glass funnel. The silica was washed twice with hot AcOEt to ensure maximal product recovery. The combined filtrates were concentrated under reduced pressure, and the resulting residue was recrystallized as described above.

#### 5-(4-Chlorophenyl)-3-(4-methoxyphenyl)-2-phenyltetrahydro-4*H*-pyrrolo­[3,4-*d*]­isoxazole-4,6­(5H)-dione **3a**.^15b^

**1** = 56.7 mg (0.25 mmol), **2** = 51.9 mg (0.25 mmol). Reaction time −1 h. Purified by recrystallization in AcOEt/hexane. White solid, (95% yield—cis/trans mixture). FT-IR (ATR, *v*_max_/cm^–1^): 1784, 1716, 1612, 1598, 1514, 1490, 1201, 817, 758. ^1^H NMR (CDCl_3,_ 500 MHz): δ 7.45 (d, *J* = 8.6 Hz, 2H), 7.37 (d, *J* = 8.6 Hz, 2H), 7.31 (dd, *J* = 15.1, 8.6 Hz, 4H), 7.27–7.20 (m, 4H), 7.11 (t, *J* = 7.0 Hz, 4H), 7.05 (t, *J* = 7.5 Hz, 3H), 6.99–6.90 (m, 3H), 6.87 (d, *J* = 8.6 Hz, 2H), 6.59 (d, *J* = 8.6 Hz, 2H), 5.68 (s, 1H), 5.24 (d, *J* = 7.7 Hz, 1H), 5.10 (d, *J* = 7.4 Hz, 1H), 4.86 (d, *J* = 9.0 Hz, 1H), 4.00 (t, *J* = 8.4 Hz, 1H), 3.97 (d, *J* = 7.4 Hz, 1H), 3.81 (s, 3H), 3.78 (s, 3H). ^13^C­{^1^H} NMR (CDCl_3,_ 126 MHz): δ 174.0, 173.4, 172.5, 171.3, 160.0, 159.5, 148.8, 147.4, 135.0, 134.6, 130.5, 129.7, 129.4, 129.3, 128.9, 128.8, 127.9, 127.5, 127.3, 126.2, 125.0, 123.0, 119.1, 114.6, 114.5, 114.4, 77.3, 76.9, 71.3, 69.7, 57.4, 55.5, 55.3, 54.7.

#### 2,3,5-Triphenyltetrahydro-4*H*-pyrrolo­[3,4-*d*]­isoxazole-4,6­(5H)-dione **3b**.^44^

**1** = 98.5 mg (0.5 mmol), **2** = 86.4 mg (0.5 mmol). Reaction time −1 h. Purified by recrystallization in AcOEt/hexane. White solid, (160 mg −87% yield—cis/trans mixture). FT-IR (ATR, *v*_max_/cm^–1^): 1788, 1716, 1595, 1583, 1489, 1384, 1184, 750, 692. ^1^H NMR (CDCl_3,_ 500 MHz): δ 7.56 (d, *J* = 7.5 Hz, 2H), 7.50–7.20 (m, 13H), 7.18–6.95 (m, 5H), 6.66–6.57 (m, 2H), 5.75 (s, 1H), 5.26 (d, *J* = 7.8 Hz, 1H), 5.09 (d, *J* = 7.5 Hz, 1H), 4.93 (d, *J* = 9.1 Hz, 1H), 4.04 (dd, *J* = 8.9, 8.0 Hz, 1H), 4.02–3.99 (m, 1H). ^13^C­{^1^H} NMR (CDCl_3,_ 126 MHz): δ 174.2, 173.5, 172.7, 171.4, 149.0, 147.5, 138.7, 134.6, 131.2, 131.0, 129.5, 129.2, 129.1, 129.1, 129.1, 129.0, 128.9, 128.8, 128.3, 127.7, 126.6, 126.2, 126.1, 124.9, 123.0, 118.9, 114.5, 77.4, 77.1, 71.6, 70.1, 57.4, 54.7.

#### 5-(4-Methoxyphenyl)-2,3-diphenyltetrahydro-4*H*-pyrrolo­[3,4-*d*]­isoxazole-4,6­(5H)-dione **3c**.^45^

**1** = 98.5 mg (0.5 mmol), **2** = 102.6 mg (0.5 mmol). Reaction time −1 h. Purified by recrystallization in AcOEt/hexane. White solid, (190 mg −96% yield—cis/trans mixture). FT-IR (ATR, *v*_max_/cm^–1^): 1791, 1710, 1635, 1591, 1512, 1257, 1249, 1201, 827, 754, 700. ^1^H NMR (CDCl_3,_ 500 MHz): δ 7.56 (d, *J* = 7.6 Hz, 2H), 7.47–7.39 (m, 4H), 7.38–7.32 (m, 3H), 7.26–7.22 (m, 4H), 7.13 (dd, *J* = 11.6, 8.1 Hz, 4H), 7.09–7.05 (m, 1H), 6.98 (s, 1H), 6.94–6.87 (m, 3H), 6.82 (d, *J* = 8.9 Hz, 2H), 6.51 (d, *J* = 8.9 Hz, 2H), 5.74 (s, 1H), 5.26 (d, *J* = 7.8 Hz, 1H), 5.09 (d, *J* = 7.5 Hz, 1H), 4.94 (d, *J* = 9.1 Hz, 1H), 4.06–4.01 (m, 1H), 3.99 (d, *J* = 7.5 Hz, 1H), 3.78 (s, 3H), 3.77 (s, 3H). ^13^C­{^1^H} NMR (CDCl_3,_ 126 MHz): δ 174.5, 173.6, 172.9, 171.6, 159.9, 159.7, 149.0, 147.6, 138.8, 134.7, 129.5, 129.1, 129.1, 128.9, 128.2, 127.9, 127.7, 127.5, 127.3, 126.6, 124.8, 123.8, 123.6, 123.0, 118.7, 114.5, 114.5, 114.4, 77.1, 71.6, 70.1, 57.4, 55.6, 55.5, 54.7.

#### 5-(4-Chlorophenyl)-2,3-diphenyltetrahydro-4*H*-pyrrolo­[3,4-*d*]­isoxazole-4,6­(5H)-dione **3d**

**1** = 98.5 mg (0.5 mmol), **2** = 103.8 mg (0.5 mmol). Reaction time −1 h. Purified by recrystallization in AcOEt/hexane. White solid, (190.4 mg −94% yield—cis/trans mixture). FT-IR (ATR, *v*_max_/cm^–1^): 1793, 1718, 1597, 1558, 1489, 1452, 1390, 823, 756, 702. ^1^H NMR (CDCl_3,_ 500 MHz): δ 7.58–6.55 (m, 11H), 7.58–6.96 (m, 11H), 5.76 (s, 1H), 5.26 (d, *J* = 7.8 Hz, 1H), 5.09 (d, *J* = 7.5 Hz, 1H), 4.94 (d, *J* = 9.1 Hz, 1H), 4.08–4.02 (m, 14H), 4.01 (d, *J* = 7.4 Hz, 14H). ^13^C­{^1^H} NMR (CDCl_3,_ 126 MHz): δ 174.0, 173.2, 172.4, 171.1, 148.9, 147.4, 138.6, 135.0, 134.7, 134.5, 129.6, 129.5, 129.4, 129.4, 129.3, 129.2, 129.1, 129.1, 129.0, 128.3, 127.6, 127.5, 127.2, 126.6, 125.0, 123.1, 118.9, 114.5, 77.3, 77.0, 71.6, 70.1, 57.4, 54.7. HRMS (ESI) *m*/*z* calcd for C_23_H_18_ClN_2_O_3_ [M + H]^+^, 405.1006; found, 405.1000.

#### 5-(4-Nitrophenyl)-2,3-diphenyltetrahydro-4*H*-pyrrolo­[3,4-*d*]­isoxazole-4,6­(5H)-dione **3e**.^45^

**1** = 69.0 mg (0.35 mmol), **2** = 76.3 mg (0.35 mmol). Reaction time −2 h. Purified by recrystallization in AcOEt/hexane. Yellow solid, (87.6 mg −89% yield—cis/trans mixture). FT-IR (ATR, *v*_max_/cm^–1^): 1789, 1716, 1612, 1597, 1523, 1489, 1342, 1180, 748, 700. ^1^H NMR (CDCl_3,_ 500 MHz): δ ^1^H NMR (500 MHz, CDCl_3_) δ 8.26 (d, *J* = 9.0 Hz, 1H), 8.19 (d, *J* = 9.0 Hz, 2H), 7.58 (d, *J* = 7.6 Hz, 2H), 7.45 (t, *J* = 7.0 Hz, 4H), 7.39 (q, *J* = 6.1 Hz, 3H), 7.33 (d, *J* = 9.0 Hz, 1H), 7.30–7.24 (m, 4H), 7.19–7.09 (m, 4H), 7.01 (t, *J* = 7.3 Hz, 1H), 6.92 (d, *J* = 9.0 Hz, 2H), 5.81 (s, 1H), 5.32 (d, *J* = 7.8 Hz, 1H), 5.16 (d, *J* = 7.5 Hz, 1H), 4.98 (d, *J* = 9.1 Hz, 1H), 4.14–4.10 (m, 1H), 4.08 (d, *J* = 7.5 Hz, 1H). ^13^C­{^1^H} NMR (CDCl_3,_ 126 MHz): δ ^13^C NMR (126 MHz, CDCl_3_) δ 173.5, 172.9, 171.9, 170.7, 148.8, 147.4, 147.1, 147.1, 138.2, 136.6, 136.3, 134.3, 129.5, 129.2, 129.2, 129.1, 129.0, 128.4, 127.5, 126.9, 126.5, 126.5, 125.2, 124.4, 124.3, 123.3, 119.1, 114.5, 77.3, 76.9, 71.6, 70.2, 57.4, 54.8.

#### 3-(4-Nitrophenyl)-2,5-diphenyltetrahydro-4*H*-pyrrolo­[3,4-*d*]­isoxazole-4,6­(5H)-dione **3f**.^45^

**1** = 84.0 mg (0.35 mmol), **2** = 60 mg (0.35 mmol). Reaction time −1 h. Purified by recrystallization in AcOEt/hexane. White solid, (126.8 mg −87% yield—cis/trans mixture). FT-IR (ATR, *v*_max_/cm^–1^): 1778, 1710, 1604, 1593, 1516, 1487, 1384, 1350, 1197, 1186, 771, 700. ^1^H NMR (CDCl_3,_ 500 MHz): δ 8.29 (d, *J* = 8.3 Hz, 2H), 8.22 (d, *J* = 8.5 Hz, 1H), 7.79 (d, *J* = 8.3 Hz, 2H), 7.64 (d, *J* = 8.5 Hz, 1H), 7.43 (t, *J* = 7.2 Hz, 1H), 7.39 (d, *J* = 6.7 Hz, 1H), 7.34 (s, 3H), 7.27 (d, *J* = 9.0 Hz, 6H), 7.17–7.00 (m, 6H), 6.65–6.57 (m, 2H), 5.85 (s, 1H), 5.12 (d, *J* = 7.3 Hz, 1H), 4.00 (d, *J* = 7.3 Hz, 1H). ^13^C­{^1^H} NMR (CDCl_3,_ 126 MHz): δ 173.6, 173.5, 173.0, 172.1, 148.3, 147.9, 145.8, 145.7, 130.8, 129.7, 129.6, 129.5, 129.4, 129.3, 129.2, 128.7, 127.8, 127.8, 126.2, 125.9, 125.8, 124.4, 124.3, 124.3, 123.6, 119.6, 116.1, 114.5, 77.3, 77.2, 70.6, 69.4, 57.3, 54.7.

#### 3-(4-Methoxyphenyl)-5-(4-nitrophenyl)-2-phenyltetrahydro-4*H*-pyrrolo­[3,4-*d*]­isoxazole-4,6­(5H)-dione **3g**

**1** = 113.6 mg (0.5 mmol), **2** = 109.8 mg (0.5 mmol). Reaction time −3 h. Purified by recrystallization in AcOEt/hexane. White solid, (204.5 mg −92% yield—cis/trans mixture). FT-IR (ATR, *v*_max_/cm^–1^): 1786, 1716, 1612, 1593, 1512, 1340, 1246, 1174, 840, 700. ^1^H NMR (CDCl_3,_ 500 MHz): δ 8.27 (d, *J* = 8.9 Hz, 2H), 8.19 (d, *J* = 8.9 Hz, 1H), 7.45 (d, *J* = 8.6 Hz, 1H), 7.38 (d, *J* = 9.0 Hz, 3H), 7.32 (d, *J* = 8.6 Hz, 3H), 7.28–7.20 (m, 4H), 7.10 (dt, *J* = 13.8, 7.7 Hz, 5H), 6.97 (t, *J* = 7.4 Hz, 1H), 6.93 (dd, *J* = 8.8, 4.0 Hz, 2H), 6.88 (d, *J* = 8.7 Hz, 2H), 5.72 (s, 1H), 5.29 (d, *J* = 7.7 Hz, 1H), 5.15 (d, *J* = 7.4 Hz, 1H), 4.89 (d, *J* = 9.0 Hz, 1H), 4.06 (t, *J* = 8.4 Hz, 1H), 3.82 (s, 2H), 3.79 (s, 4H). ^13^C­{^1^H} NMR (CDCl_3,_ 126 MHz): δ 173.6, 173.1, 172.0, 170.9, 160.1, 159.6, 148.7, 147.5, 147.2, 147.1, 136.6, 136.4, 130.2, 129.5, 128.9, 128.7, 127.9, 127.0, 126.5, 125.9, 125.2, 124.5, 124.3, 123.2, 119.3, 114.7, 114.6, 114.5, 77.2, 76.8, 71.3, 69.9, 57.4, 55.5, 55.4, 54.8. HRMS (ESI) *m*/*z* calcd for C_24_H_20_N_3_O_6_ [M + H]^+^, 446.1352; found, 446.1346.

#### 3,5-Bis­(4-methoxyphenyl)-2-phenyltetrahydro-4*H*-pyrrolo­[3,4-*d*]­isoxazole-4,6­(5H)-dione **3h**.^15b^

**1** = 56.7 mg (0.25 mmol), **2** = 51.3 mg (0.25 mmol). Reaction time −1 h. Purified by recrystallization in AcOEt/hexane. White solid, (87% yield—cis/trans mixture). FT-IR (ATR, *v*_max_/cm^–1^): 1795, 1712, 1612, 1591, 1512, 1489, 1300, 1253, 761. ^1^H NMR (CDCl_3,_ 500 MHz): δ 7.45 (d, *J* = 8.4 Hz, 2H), 7.34 (d, *J* = 8.4 Hz, 2H), 7.23 (t, *J* = 8.7 Hz, 5H), 7.17–7.04 (m, 5H), 7.03–6.87 (m, 8H), 6.82 (d, *J* = 8.6 Hz, 3H), 6.54 (d, *J* = 8.6 Hz, 2H), 5.67 (s, 1H), 5.23 (d, *J* = 7.0 Hz, 1H), 5.09 (d, *J* = 7.1 Hz, 1H), 4.86 (d, *J* = 8.7 Hz, 1H), 4.03–3.97 (m, 1H), 3.95 (d, *J* = 7.1 Hz, 1H), 3.81 (s, 3H), 3.79 (s, 3H), 3.78 (s, 3H), 3.77 (s, 3H). ^13^C­{^1^H} NMR (CDCl_3,_ 126 MHz): δ 174.4, 173.7, 172.9, 171.7, 159.8, 159.7, 159.5, 159.4, 148.8, 147.4, 130.6, 129.3, 128.7, 127.8, 127.3, 127.2, 126.3, 124.7, 123.8, 123.5, 122.8, 118.8, 114.5, 114.5, 114.4, 114.4, 114.3, 114.2, 77.2, 76.8, 71.1, 69.6, 57.2, 55.4, 55.4, 55.3, 55.2, 54.5.

#### 3-(Furan-2-yl)-2,5-diphenyltetrahydro-4*H*-pyrrolo­[3,4-*d*]­isoxazole-4,6­(5H)-dione **3i**.^44^

**1** = 93.6 mg (0.5 mmol), **2** = 86.5 mg (0.5 mmol), Silica = 200 mg. Reaction time −2 h. Purified by column chromatography (hexane/EtOAc 7:3). *cis* isomer—White solid, (29.2 mg −16% yield) mp: 181.5–183.9 °C. FT-IR (ATR, *v*_max_/cm^–1^): 1793, 1718, 1595, 1490, 1386, 1190, 1101, 769, 727, 694. ^1^H NMR (CDCl_3,_ 500 MHz): δ 7.51–7.42 (m, 3H), 7.39 (t, *J* = 7.4 Hz, 1H), 7.32–7.23 (m, 4H), 7.11 (t, *J* = 7.4 Hz, 1H), 7.08–7.01 (m, 2H), 6.40–6.33 (m, 2H), 5.27 (d, *J* = 7.8 Hz, 1H), 4.87 (d, *J* = 8.9 Hz, 1H), 4.07–3.98 (m, 1H). ^13^C­{^1^H} NMR (CDCl_3,_ 126 MHz): δ 173.3, 171.6, 147.1, 146.9, 143.5, 131.5, 129.3, 129.0, 128.9, 126.3, 125.4, 118.9, 111.0, 110.9, 76.8, 66.8, 52.8. *trans* isomer—White solid, (33.1 mg – 18,3% yield), mp: 164.0–166.7 °C. FT-IR (ATR, *v*_max_/cm^–1^): 1774, 1716, 1705, 1595, 1558, 1496, 1377, 1251, 1193, 769, 727, 721, 690, 673. ^1^H NMR (CDCl_3,_ 500 MHz): δ 7.40 (dd, *J* = 1.6, 0.7 Hz, 1H), 7.34 (tdd, *J* = 4.8, 3.6, 1.7 Hz, 3H), 7.28–7.21 (m, 2H), 7.13–7.08 (m, 2H), 7.00 (t, *J* = 7.4 Hz, 1H), 6.80–6.73 (m, 2H), 6.36–6.30 (m, 2H), 5.68 (s, 1H), 5.20 (d, *J* = 7.5 Hz, 1H), 4.21–4.16 (m, 1H). ^13^C­{^1^H} NMR (CDCl_3,_ 126 MHz): δ 173.9, 172.8, 150.4, 147.7, 142.9, 131.1, 129.3, 129.1, 129.1, 126.3, 123.5, 115.4, 110.8, 108.6, 77.0, 64.4, 53.8.

#### 5-(4-Chlorophenyl)-3-(furan-2-yl)-2-phenyltetrahydro-4*H*-pyrrolo­[3,4-*d*]­isoxazole-4,6­(5H)-dione **3j**

**1** = 46.8 mg (0.25 mmol), **2** = 52.0 mg (0.25 mmol), Silica = 100 mg. Reaction time −3 h. Purified by column chromatography (hexane/EtOAc 7:3). *cis* isomer—White solid, (24.6 mg −25% yield), mp: 160.1–161.1 °C. FT-IR (ATR, *v*_max_/cm^–1^): 1799, 1724, 1593, 1489, 1390, 1190, 1101, 1064, 827, 798, 758. ^1^H NMR (CDCl_3,_ 500 MHz): δ 7.43 (dt, *J* = 7.8, 2.6 Hz, 3H), 7.29–7.21 (m, 4H), 7.14–7.09 (m, 1H), 7.06–7.03 (m, 1H), 7.03 (s, 1H), 6.36 (d, *J* = 1.3 Hz, 2H), 5.26 (d, *J* = 7.8 Hz, 1H), 4.84 (d, *J* = 8.9 Hz, 1H), 4.01 (dd, *J* = 8.8, 7.9 Hz, 1H). ^13^C­{^1^H} NMR (CDCl_3,_ 126 MHz): δ 173.1, 171.3, 147.0, 146.7, 143.6, 134.8, 129.9, 129.5, 128.9, 127.5, 125.5, 118.9, 111.0, 111.0, 76.7, 66.9, 52.8. *trans* isomer—White solid, (15.3 mg—16% yield), mp: 160.7–161.2 °C. FT-IR (ATR, *v*_max_/cm^–1^): 1786, 1710, 1599, 1493, 1392, 1182, 1091, 1010, 764, 742, 690. ^1^H NMR (CDCl_3,_ 500 MHz): δ 7.41 (d, *J* = 1.0 Hz, 1H), 7.35–7.30 (m, 2H), 7.27–7.21 (m, 2H), 7.12–7.08 (m, 2H), 6.99 (t, *J* = 7.4 Hz, 1H), 6.74–6.69 (m, 2H), 6.36–6.31 (m, 2H), 5.68 (s, 1H), 5.20 (d, *J* = 7.5 Hz, 1H), 4.21–4.18 (m, 1H). ^13^C­{^1^H} NMR (CDCl_3,_ 126 MHz): δ 173.5, 172.4, 150.1, 147.5, 142.9, 134.9, 129.3, 129.2, 127.4, 123.4, 115.2, 110.7, 108.5, 76.8, 64.3, 53.6. HRMS (ESI) *m*/*z* calcd for C_21_H_15_ClN_2_NaO_4_ [M + Na]^+^, 417.0618; found, 417.0612.

#### 3-(4-Methoxyphenyl)-2,5-diphenyltetrahydro-4*H*-pyrrolo­[3,4-*d*]­isoxazole-4,6­(5H)-dione **3k.** ^45^

**1** = 56.7 mg (0.25 mmol), **2** = 43.2 mg (0.25 mmol). Reaction time −1 h. Purified by recrystallization in AcOEt/hexane. White solid, (80.3 mg −81% yield -*cis* isomer), mp: 189.3–191.5 °C (Lit.^45^ 193–195 °C). FT-IR (ATR, *v*_max_/cm^–1^): 1799, 1720, 1614, 1597, 1514, 1390, 1249, 1182, 1170, 819, 756, 690. ^1^H NMR (CDCl_3,_ 500 MHz): δ 7.40 (d, *J* = 7.3 Hz, 2H), 7.35 (d, *J* = 7.8 Hz, 3H), 7.27–7.20 (m, 2H), 7.15–7.04 (m, 5H), 6.88 (d, *J* = 8.0 Hz, 2H), 5.25 (d, *J* = 7.3 Hz, 1H), 4.86 (d, *J* = 8.7 Hz, 1H), 4.01 (t, *J* = 7.9 Hz, 1H), 3.78 (s, 3H). ^13^C­{^1^H} NMR (CDCl_3,_ 126 MHz): δ 173.7, 171.6, 159.9, 147.5, 131.3, 129.2, 128.9, 126.3, 126.1, 124.9, 119.1, 114.5, 77.0, 71.3, 55.3, 54.7.

#### 5-(4-Methoxyphenyl)-3-(4-nitrophenyl)-2-phenyltetrahydro-4*H*-pyrrolo­[3,4-d]­isoxazole-4,6­(5H)-dione **3l**.

**1** = 121.1 mg (0.5 mmol), **2** = 102.63 mg (0.5 mmol). Reaction time −2 h. Purified by recrystallization in AcOEt/hexane. White solid, (213.0 mg −96% yield—*trans* isomer), mp: 213.6–217.3 °C. FT-IR (ATR, *v*_max_/cm^–1^): 1780, 1708, 1614, 1539, 1514, 1487, 1390, 1350, 1255, 1195, 821, 769, 758, 700. ^1^H NMR (CDCl_3,_ 500 MHz): δ 8.28 (d, *J* = 8.4 Hz, 2H), 7.78 (d, *J* = 8.4 Hz, 2H), 7.27 (d, *J* = 9.0 Hz, 3H), 7.14 (d, *J* = 8.0 Hz, 2H), 7.03 (t, *J* = 7.3 Hz, 1H), 6.83 (d, *J* = 8.6 Hz, 2H), 6.50 (d, *J* = 8.6 Hz, 2H), 5.84 (s, 1H), 5.09 (d, *J* = 7.4 Hz, 1H), 3.98 (d, *J* = 7.4 Hz, 1H), 3.78 (s, 3H). ^13^C­{^1^H} NMR (CDCl_3,_ 126 MHz): δ 173.8, 172.4, 160.0, 148.3, 147.9, 145.8, 129.7, 127.8, 127.4, 124.3, 123.5, 123.3, 114.5, 114.5, 77.3, 69.3, 57.3, 55.6. HRMS (ESI) *m*/*z* calcd for C_24_H_18_N_3_O_6_ [M – H]^−^, 444.1196; found, 444.12011.

#### 3,5-Bis­(4-nitrophenyl)-2-phenyltetrahydro-4*H*-pyrrolo­[3,4-*d*]­isoxazole-4,6­(5H)-dione **3m**

**1** = 100.0 mg (0.4 mmol), 2 = 90.1 mg (0.4 mmol). Reaction time −3 h. Purified by recrystallization in AcOEt/hexane. White solid, (129.0 mg −70% yield—*trans* isomer), mp: Decomposes above 207 °C. FT-IR (ATR, *v*_max_/cm^–1^): 1784, 1720, 1595, 1529, 1514, 1487, 1373, 1344, 1186, 848, 773, 748. ^1^H NMR (CDCl_3,_ 500 MHz): δ 8.31 (dd, *J* = 8.6, 4.0 Hz, 2H), 8.21 (d, *J* = 8.9 Hz, 2H), 7.84–6.85 (m, 7H), 6.87 (d, *J* = 8.9 Hz, 2H), 5.91 (s, 1H), 5.15 (d, *J* = 7.5 Hz, 2H), 4.06 (d, *J* = 7.5 Hz, 2H). ^13^C­{^1^H} NMR (CDCl_3,_ 126 MHz): δ 172.9, 172.6, 148.0, 147.9, 147.5, 145.3, 136.1, 129.8, 127.9, 127.0, 124.5, 124.4, 124.0, 114.3, 77.1, 69.4, 57.0. HRMS (ESI) *m*/*z* calcd for C_23_H_15_N_4_O_7_ [M – H]^−^, 459.0946; found, 459.0939.

#### 3-(4-Methoxyphenyl)-2-methyl-5-phenyltetrahydro-4*H*-pyrrolo­[3,4-*d*]­isoxazole-4,6­(5H)-dione **3n**.^16^

**1** = 82.5 mg (0.5 mmol), **2** = 86.5 mg (0.5 mmol), Silica = 200 mg. Reaction time −2 h. Purified by recrystallization in AcOEt/hexane. White solid, (113.2 mg −67% yield—*cis* isomer), mp: 154.5–157.3 (Lit.^16^ 165 °C). FT-IR (ATR, *v*_max_/cm^–1^): 1793, 1708, 1610, 1585, 1512, 1386, 1247, 1203, 1180, 1103, 1022, 871, 840, 815, 759, 692, 624. ^1^H NMR (CDCl_3,_ 500 MHz): δ 7.45 (t, *J* = 7.7 Hz, 2H), 7.37 (t, *J* = 7.4 Hz, 1H), 7.24 (dd, *J* = 15.7, 8.1 Hz, 4H), 6.89 (d, *J* = 8.5 Hz, 2H), 5.03 (d, *J* = 7.3 Hz, 1H), 3.91 (d, *J* = 8.7 Hz, 1H), 3.82 (d, *J* = 7.9 Hz, 1H), 3.79 (s, 3H), 2.68 (s, 3H). ^13^C­{^1^H} NMR (CDCl_3,_ 126 MHz): δ 174.8, 172.3, 160.0, 131.4, 129.3, 129.0, 128.8, 126.1, 125.2, 114.4, 76.5, 75.3, 55.3, 54.3, 42.6.

#### 5-(4-Chlorophenyl)-3-(4-methoxyphenyl)-2-methyltetrahydro-4*H*-pyrrolo­[3,4-*d*]­isoxazole-4,6­(5H)-dione **3o**

**1** = 82.5 mg (0.5 mmol), **2** = 103.8 mg (0.5 mmol), Silica = 200 mg. Reaction time −2 h. Purified by recrystallization in AcOEt/hexane. Colorless crystals, (116.6 mg—62,5% yield—*cis* isomer), mp: 184.7–186.7 °C. FT-IR (ATR, *v*_max_/cm^–1^): 1786, 1718, 1489, 1371, 1178, 1047, 854, 833, 781, 700, 611. ^1^H NMR (CDCl_3,_ 500 MHz): δ 7.41 (d, *J* = 8.6 Hz, 2H), 7.21 (dd, *J* = 14.5, 8.6 Hz, 4H), 6.88 (d, *J* = 8.6 Hz, 2H), 5.02 (d, *J* = 7.4 Hz, 1H), 3.91 (d, *J* = 8.8 Hz, 1H), 3.82 (d, *J* = 8.0 Hz, 1H), 3.79 (s, 3H), 2.68 (s, 3H). ^13^C­{^1^H} NMR (CDCl_3,_ 126 MHz): δ 174.5, 172.0, 160.0, 134.5, 129.8, 129.5, 128.9, 127.3, 125.1, 114.4, 114.4, 76.4, 76.3, 75.3, 75.2, 55.3, 55.2, 54.4, 54.2, 42.7, 42.6. HRMS (ESI) *m*/*z* calcd for C_19_H_18_ClN_2_O_4_ [M + H]^+^, 373.0955; found, 373.0949.

#### 5-(4-Chlorophenyl)-2-methyl-3-phenyltetrahydro-4*H*-pyrrolo­[3,4-*d*]­isoxazole-4,6­(5H)-dione **3p**

**1** = 33.7 mg (0.25 mmol), **2** = 51.9 mg (0.25 mmol), Silica = 100 mg. Reaction time −3 h. Purified by recrystallization in AcOEt/hexane. Colorless crystals, (70.1 mg—84% yield—*cis* isomer), mp: 152.0–153.6 °C. FT-IR (ATR, *v*_max_/cm^–1^): 1786, 1714, 1610, 1538, 1514, 1492, 1386, 1251, 1182, 1172, 1028, 873, 844, 817. ^1^H NMR (CDCl_3,_ 500 MHz): δ 7.39 (t, *J* = 8.3 Hz, 2H), 7.35 (t, *J* = 7.4 Hz, 3H), 7.31–7.24 (m, 2H), 7.20 (d, *J* = 8.6 Hz, 2H), 5.02 (d, *J* = 7.3 Hz, 1H), 3.95 (d, *J* = 8.7 Hz, 1H), 3.84 (t, *J* = 8.0 Hz, 1H), 2.70 (s, 3H). ^13^C­{^1^H} NMR (CDCl_3,_ 126 MHz): δ 174.4, 171.8, 134.5, 133.6, 129.9, 129.4, 129.2, 129.0, 127.8, 127.3, 76.4, 75.7, 54.5, 42.7. HRMS (ESI) *m*/*z* calcd for C_18_H_16_ClN_2_O_3_ [M + H]^+^, 343.0849; found, 343.0854.

#### 3,5-Bis­(4-methoxyphenyl)-2-methyltetrahydro-4*H*-pyrrolo­[3,4-*d*]­isoxazole-4,6­(5H)-dione **3q**

**1** = 82.5 mg (0.5 mmol), **2** = 101.5 mg (0.5 mmol), Silica = 200 mg. Reaction time −2 h. Purified by recrystallization in AcOEt/hexane. White solid, 80.6 mg—44% yield—*cis* isomer), mp: 172.0–173.0 °C. FT-IR (ATR, *v*_max_/cm^–1^): 1784, 1707, 1612, 1587, 1510, 1301, 1247, 1195, 1166, 1028, 833, 800. ^1^H NMR (CDCl_3,_ 500 MHz): δ 7.23 (d, *J* = 8.3 Hz, 2H), 7.20 (d, *J* = 8.6 Hz, 2H), 6.97 (d, *J* = 8.6 Hz, 2H), 6.90 (d, *J* = 8.1 Hz, 2H), 5.05–5.00 (m, 1H), 3.93–3.89 (m, 1H), 3.83 (s, 4H), 3.81 (s, 4H), 2.70 (s, 3H). ^13^C­{^1^H} NMR (CDCl_3,_ 126 MHz): δ 174.9, 172.4, 160.0, 159.6, 129.0, 127.4, 125.4, 124.2, 114.6, 114.4, 76.5, 75.4, 55.6, 55.3, 54.4, 42.7. HRMS (ESI) *m*/*z* calcd for C_20_H_21_N_2_O_5_ [M + H]^+^, 369.1450; found, 369.1445.

#### 5-(4-Methoxyphenyl)-2-methyl-3-phenyltetrahydro-4*H*-pyrrolo­[3,4-*d*]­isoxazole-4,6­(5H)-dione **3r**

**1** = 67.5 mg (0.5 mmol), **2** = 101.5 mg (0.5 mmol), Silica = 200 mg. Reaction time −2 h Purified by recrystallization in AcOEt/hexane. White solid, 145.6 mg—86% yield (*cis* isomer), mp: 204.0–206.0 °C. FT-IR (ATR, *v*_max_/cm^–1^): 1784, 1710, 1606, 1589, 1508, 1454, 1379, 1242, 1198, 1166, 1024, 833, 754, 700. ^1^H NMR (CDCl_3,_ 500 MHz): δ 7.41–7.31 (m, 5H), 7.18 (d, *J* = 8.9 Hz, 2H), 6.96 (d, *J* = 8.9 Hz, 2H), 5.04 (d, *J* = 7.4 Hz, 1H), 3.96 (d, *J* = 8.7 Hz, 1H), 3.88–3.84 (m, 1H), 3.83 (s, 3H), 2.73 (s, 3H). ^13^C­{^1^H} NMR (CDCl_3,_ 126 MHz): δ 174.8, 172.2, 159.6, 133.7, 129.0, 129.0, 127.8, 127.3, 124.1, 114.6, 76.5, 75.7, 55.6, 54.5, 42.7. HRMS (ESI) *m*/*z* calcd for C_19_H_19_N_2_O_4_ [M + H]^+^, 339.1345; found, 339.1339.

### Scaled Mechanochemical Synthesis

#### Procedure to Scaled Mechanochemical Synthesis of 3-(4-Methoxyphenyl)-2,5-diphenyltetrahydro-4*H*-pyrrolo­[3,4-*d*]­isoxazole-4,6­(5H)-dione **3k**.^45^

In the 12 mL stainless steel container of the ball mill, the nitrone **1a**, the maleimide **2b**, and 10 stainless steel spheres (5 mm diameter) were added. The reactor was placed in a planetary ball mill, and the parameters were adjusted to a rotation speed of 550 rpm, with directional inversion every 30 min. When necessary, silica (SiO_2_) was added as a milling auxiliary (as indicated in each case). After grinding, the crude mixture was dissolved in ethyl acetate (AcOEt) and transferred to a 25 mL round-bottomed flask, followed by concentration under reduced pressure. The crude product was then recrystallized from hexane/ethyl acetate to afford a white solid. In cases where silica (SiO_2_) was used as a milling auxiliary, the reaction mixture was dissolved in ethyl acetate (AcOEt) and transferred to a sintered glass funnel. The silica was washed twice with hot AcOEt to ensure a maximal product recovery. The combined filtrates were concentrated under reduced pressure, and the resulting residue was recrystallized, as described above.

Condition A: **1** = 228 mg (1 mmol), **2** = 173 mg (1 mmol). Reaction time −1 h. Purified by recrystallization in AcOEt/hexane. White solid (168 mg—42% yield—*cis* isomer), mp: 195–197 °C (Lit.^45^ 193–195 °C).

Condition B: **1** = 228 mg (1 mmol), **2** = 173 mg (1 mmol). Silica = 400 mg. Reaction time −1 h. Purified by recrystallization in AcOEt/hexane. White solid, (196.8 mg—49.2% yield—*cis* isomer), mp: 196–198 °C.

Condition C: **1** = 228 mg (1 mmol), **2** = 173 mg (1 mmol). Reaction time −2 h. Purified by recrystallization in AcOEt/hexane. White solid, (316 mg—79% yield -*cis* isomer), mp: 195–197 °C.

Condition D: **1** = 2.28 g (10 mmol), **2** = 1.73 g (10 mmol). Silica = 2 g. 50 mL stainless steel container. Twenty stainless steel spheres (5 mm diameter). Reaction time −16 h. Purified by recrystallization in AcOEt/hexane. White solid, (2.61 g—65% yield -*cis* isomer), mp: 195–197 °C.

## Supplementary Material



## Data Availability

The data underlying this study are available in the published article and its online Supporting Information.

## Notes

Selected experimental procedures of compounds **1**–**6** and copies of NMR spectra mentioned throughout the text and detailed green metrics to all products and scale up of **3k** (PDF)
