# Supplementary material for: Mechanochemical Diastereoselective Substrate-Directed Synthesis of Pyrrole-Isoxazolidines via 1,3-Dipolar Cycloaddition of Maleimides with Nitrones
Source: J Org Chem. 2026 Jul 27;91(31):10595–606. doi: 10.1021/acs.joc.6c00411 (PMC13459418; doi:10.1021/acs.joc.6c00411)
Supplement: Supplementary file 1 [file jo6c00411_si_001.pdf]

# Supporting Information

## Mechanochemical Diastereoselective Substrate-Directed Synthesis of Pyrrole-Isoxazolidines via 1,3-Dipolar Cycloaddition of Maleimides with Nitrones

Iva S. de Jesus[1], Anna Flávia da C. Lédo[2], Armando Ferrer[1], Mauro A. Bueno[2] and Silvio Cunha*[1]

[1]Instituto de Química, Universidade Federal da Bahia, Campus de Ondina, Salvador, Bahia, 40170-115, Brazil

[2]Universidade Federal do Oeste da Bahia, Rua da Prainha, Morada Nobre, Barreiras, Bahia, CEP 47810-059, Brazil

silviodc@ufba.br

1. **General Procedure for the synthesis of nitrones ..........S1**
2. **General Procedure for the synthesis of *N*-aryl maleimides..........S3**
3. **Experiment setup - synthesis of compound 3i..........S4**
4. **$^{1}$H, $^{13}$C NMR and HRMS spectra ..........S5**
5. **Scale-up experiments and Green Chemistry Metrics..........S75**

## 1. General Procedure for the synthesis of nitrones

### *1.1 C–N-Diarylnitrones*

A solution of nitrobenzene (3.4 mL, 32.4 mmol) in absolute EtOH (20 mL) was prepared in a 100 mL one-neck round-bottom flask equipped with magnetic stirring. An aqueous solution of $NH_4Cl$ (2.080 g, 37 mmol) was added under vigorous stirring. The suspension was cooled to 0 °C, and Zn powder (5.1 g, 78 mmol) was added slowly. After stirring for 1.5 h at ambient temperature, formation of a white suspension was observed. The corresponding aldehyde and AcOH (20 mL, glacial) were then added, and the mixture was stirred for an additional 30 min at ambient temperature. The reaction mixture was transferred to a separatory funnel containing toluene (20 mL), and the organic layer was washed with $H_2O$ (2 × 20 mL), saturated $NaHCO_3$(aq) (20 mL), and saturated NaCl(aq) (20 mL). The organic phase was dried over $MgSO_4$, filtered, and concentrated under reduced pressure. Precipitation of the nitrone was achieved by addition of hexane. The solid was collected by vacuum filtration and purified as required.[1]

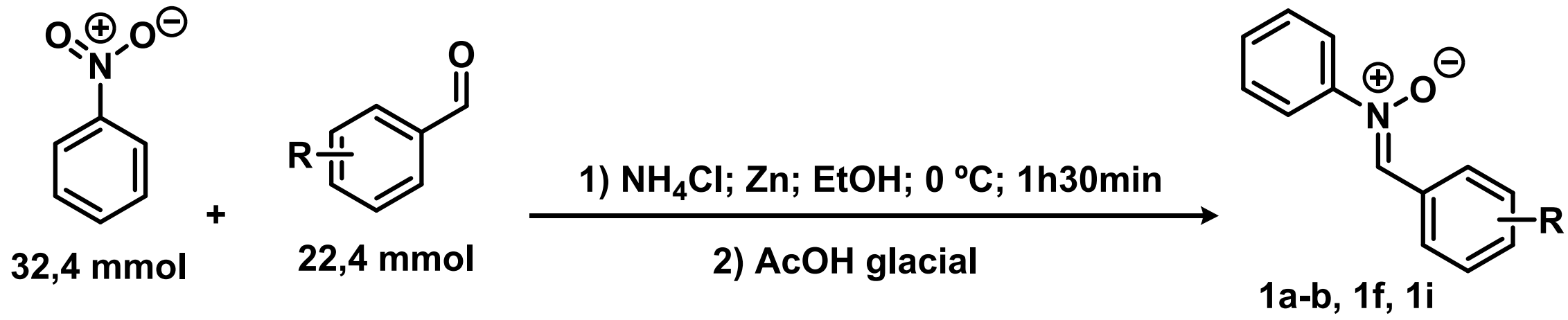


**Scheme S1.** Synthesis of C-*N*-Diarylnitrones.

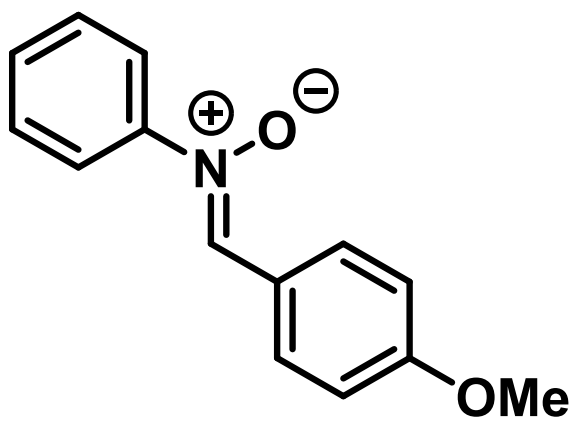

***(Z)-1-(4-methoxyphenyl)-N-phenylmethanimine oxide (1a):*** 2,23 g, 44% yield, colorless needles, purified by recrystallization from ethanol, followed by recrystallization from hot hexane, mp: 115.7-117.6°C (Lit.[1] 115-116°C). FT-IR (ATR, *v*max/cm$^{-1}$): 2931, 1600, 1504, 1458, 1396, 1257, 1173, 1064, 852, 756.

[1] Pinto, R. M. G. C. *Síntese Selectiva de Moléculas com Potencial Interesse Biológico: Desenvolvimento de Metodologias Sustentáveis*; Ph.D. Dissertation, Universidade Nova de Lisboa, Lisboa, 2012.

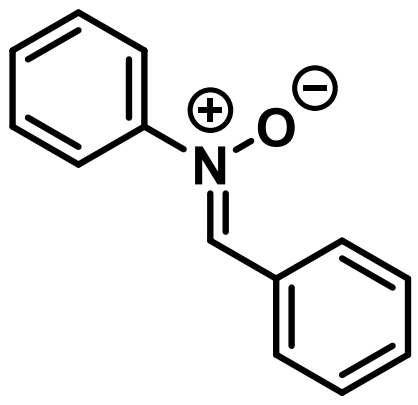

*(Z)-N,1-diphenylmethanimine oxide (1b):*

2,0 g, 45% yield, colorless needles, purified by recrystallization from ethanol, followed by recrystallization from hot hexane, mp: 111.2-113.0°C (Lit.[1] 110°C). **FT-IR** (ATR, *v*max/cm$^{-1}$): 3055, 1547, 1485, 1458, 1443, 1396.

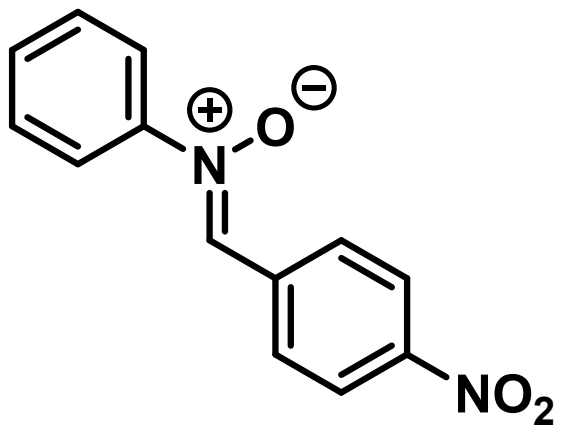

*(Z)-1-(4-nitrophenyl)-N-phenylmethanimine oxide (1f):*

0,50 g, 9% yield, yellow needles, purified by recrystallization from etanol. The unreacted *p*-nitrobenzaldehyde was recovered. mp: 187.6-189.6°C (Lit.[1] 183-184°C). FT-IR (ATR, *v*max/cm$^{-1}$): 1595, 1508, 1458, 1402, 1340, 1330, 1192, 1072, 858, 773, 690

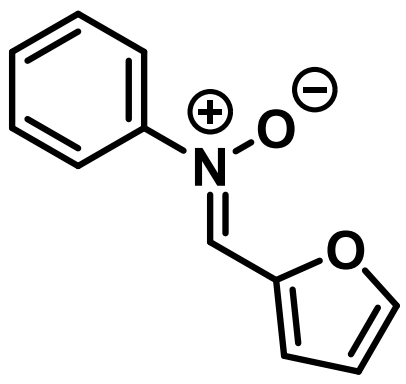

*(Z)-1-(furan-2-yl)-N-phenylmethanimine oxide (1i):*

1,68 g, 40% yield, colorless needles, purified by column chromatography (hexane/EtOAc 8:2), followed by recrystallization from hexane. mp: 90.1-90.7°C (Lit.[1] 86-87°C). FT-IR (ATR, *v*max/cm$^{-1}$): 3097, 3055, 1593, 1570, 1543, 1473, 1388, 1296, 1157, 1068, 871, 763

### *1.2 C-aryl-N-alkylnitrones*

In a 50 mL single-neck round-bottom flask equipped with magnetic stirring, an aqueous solution containing NaOH (0.48 g, 12 mmol), distilled water (20 mL), and *N*-methylhydroxylamine hydrochloride (1.0 g, 12 mmol) was prepared. The corresponding aldehyde was then added under stirring. The reaction mixture was stirred at room temperature for 1 h. After completion of the reaction, the mixture was transferred to a separatory funnel and extracted with $CH_2Cl_2$ (3 × 20 mL). The combined organic layers were dried over anhydrous $MgSO_4$, filtered, and concentrated under reduced pressure.[1] Each nitrone was subsequently purified according to its specific procedure.

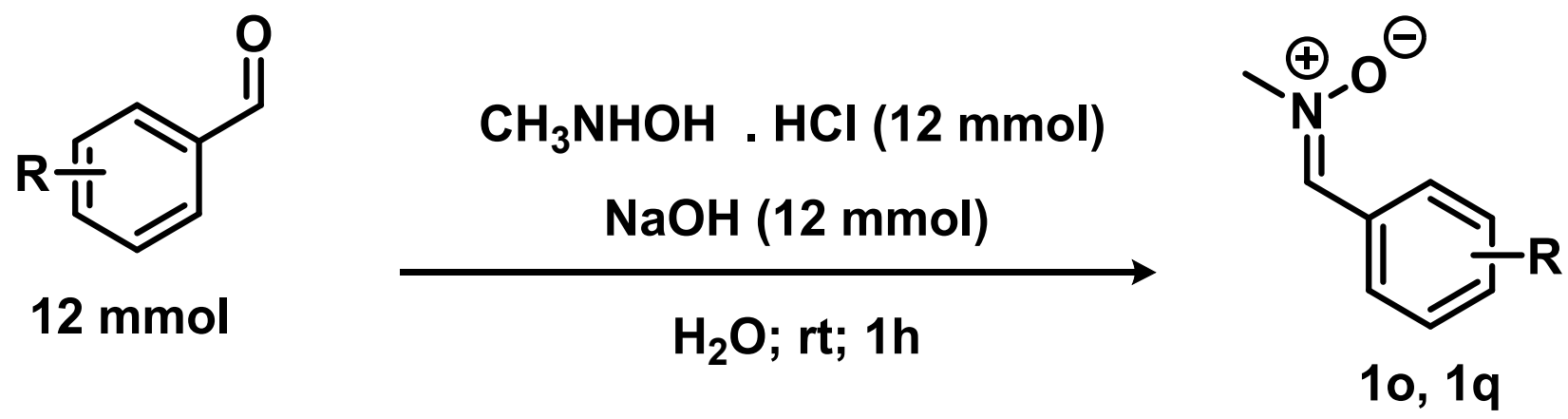

**Scheme S2.** Synthesis of C-aryl-*N*-alkylnitrones.

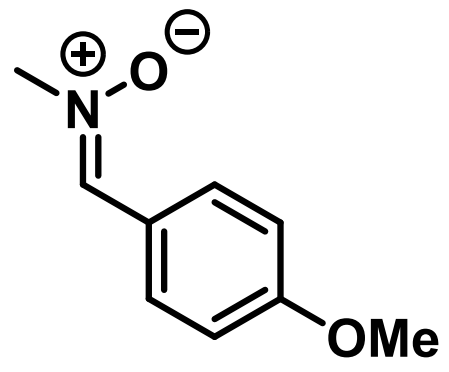

***(Z)-1-(4-methoxyphenyl)-N-methylmethanimine oxide (1o):***

1,98 g, 60% yield, colorless needles, purified by column chromatography (EtOAc/Methanol 9:1). mp: 70.2-71.7°C (**Lit.**[1] 72-73°C). **FT-IR** (ATR, *v*max/cm$^{-1}$): 3012, 2943, 1672, 1600, 1508, 1462.

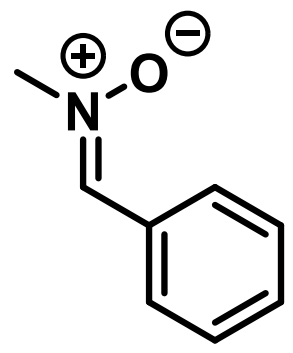

***(Z)-N-methyl-1-phenylmethanimine oxide (1q):***

0,93 g, 29% yield, colorless needles, purified by column chromatography (EtOAct), followed by recrystallization from hexane. mp: 82.8-84.1°C (Lit.[1] 83-84°C). FT-IR (ATR, *v*max/cm$^{-1}$): 3057, 2943, 1595, 1444, 1419, 1400, 1300, 1163.

## 2. General Procedure for the synthesis of *N*-aryl maleimides

The experimental procedure for the synthesis of maleimides was performed based on the method described by Deshpande and co-workers.[2]

## 3. Experiment setup - synthesis of compound 3i:

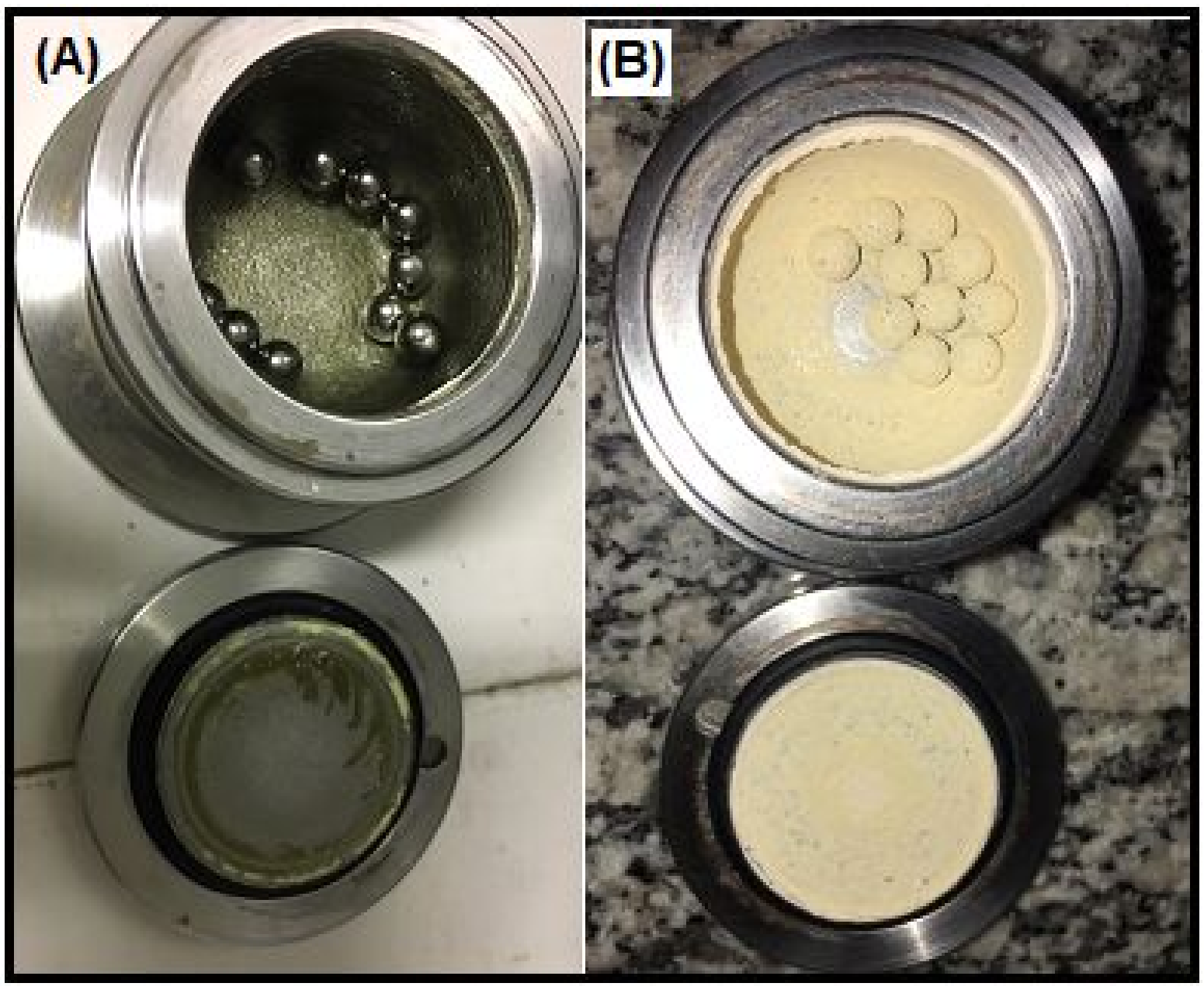


**Figure S1:** Reaction for the synthesis of compound **3i** after 1 hour of reaction (A) without the use of silica and (B) with the use of silica.

[2] Deshpande, R.; Maybhate, P.; Likhite, P.; Chaudhary, M. A Facile Synthesis of N-Substituted Maleimides. *Indian J. Chem., Sect. B* **2010**, *49* (4), 487–488.

## 4. $^{1}$H, $^{13}$C NMR and FT-IR spectra

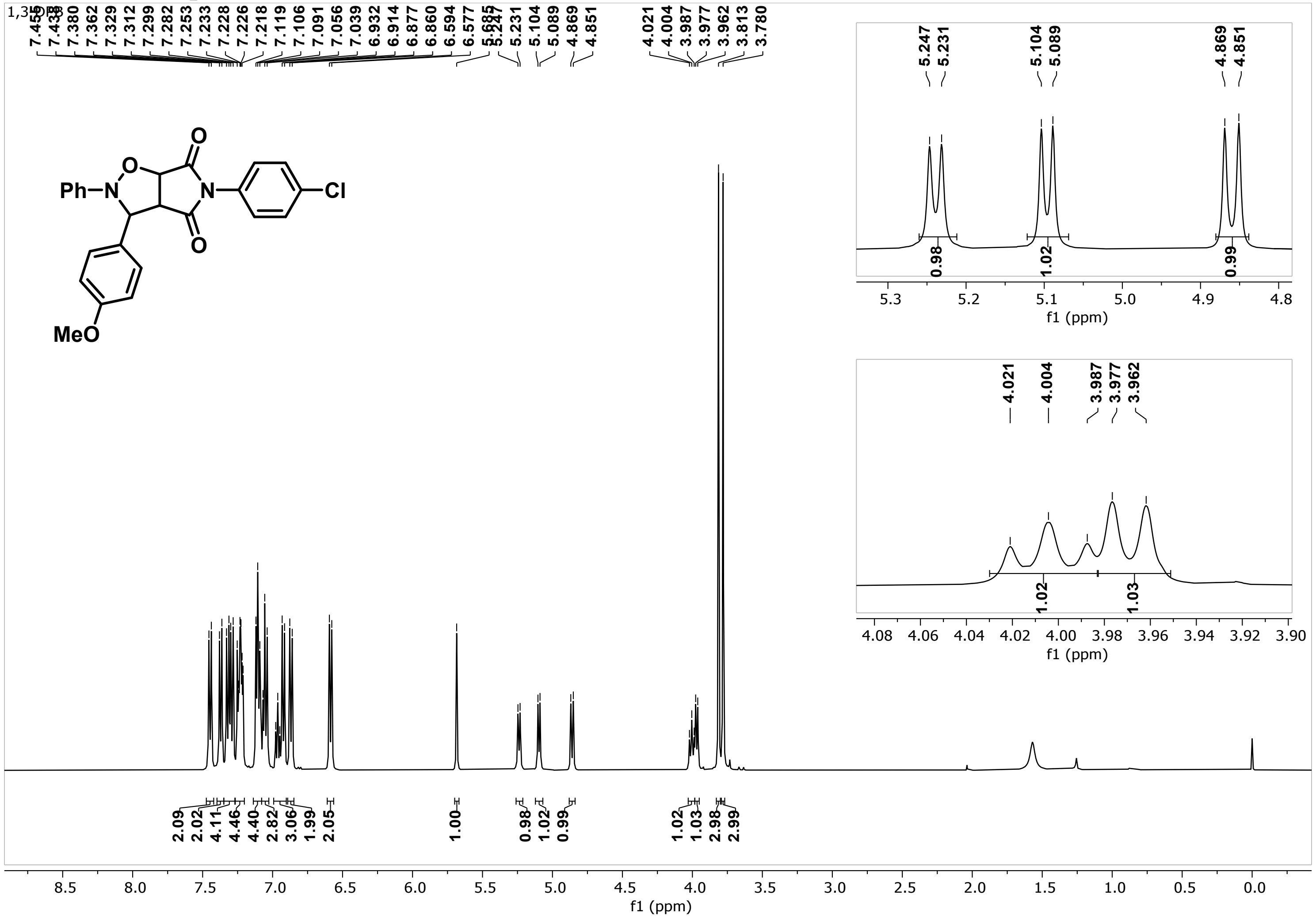

**Figure S10.** $^{1}$H NMR spectrum of **3a** (500 MHz, $CDCl_3$).

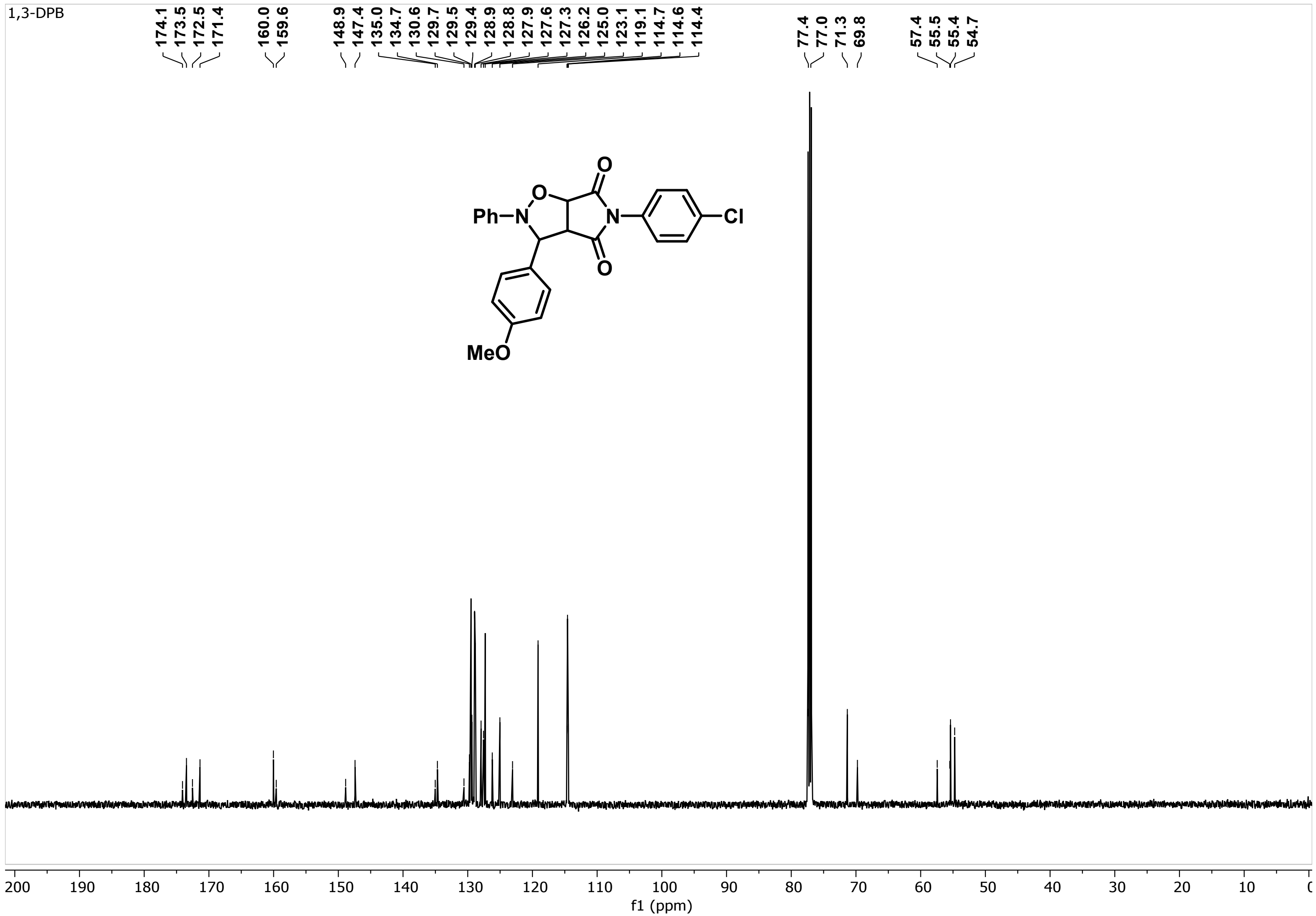


**Figure S11.** $^{13}$C NMR spectrum of **3a** (125 MHz, $CDCl_3$).

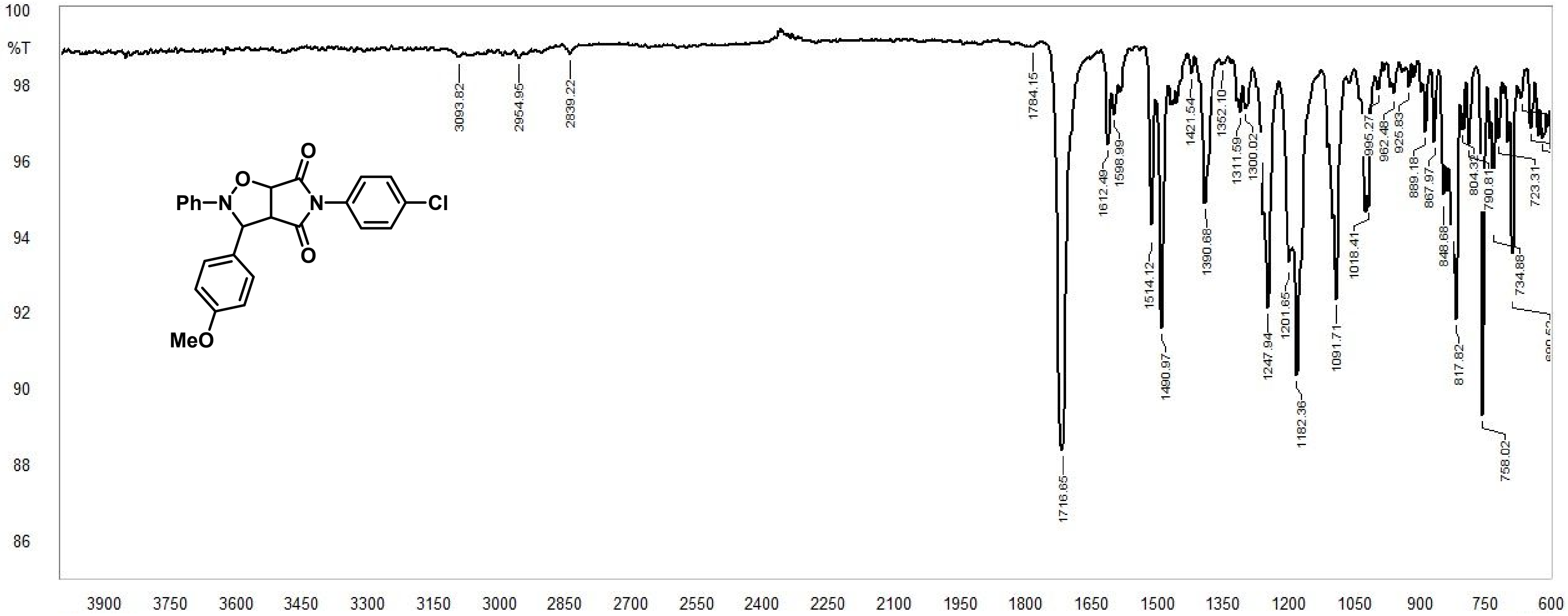


**Figure S12.** FT-IR spectrum of **3a**.

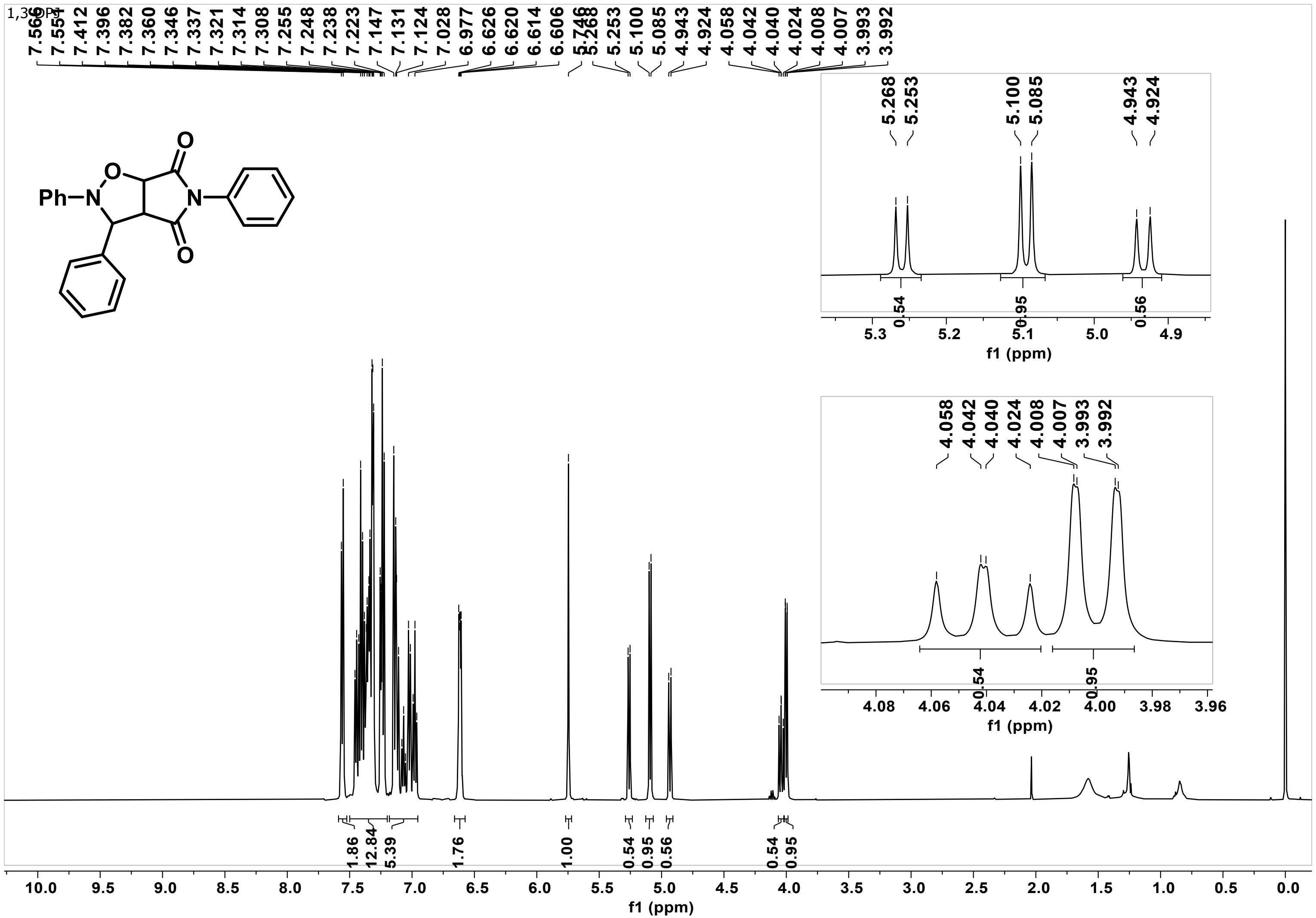

**Figure S13.** $^1$H NMR spectrum of **3b** (500 MHz, $CDCl_3$).

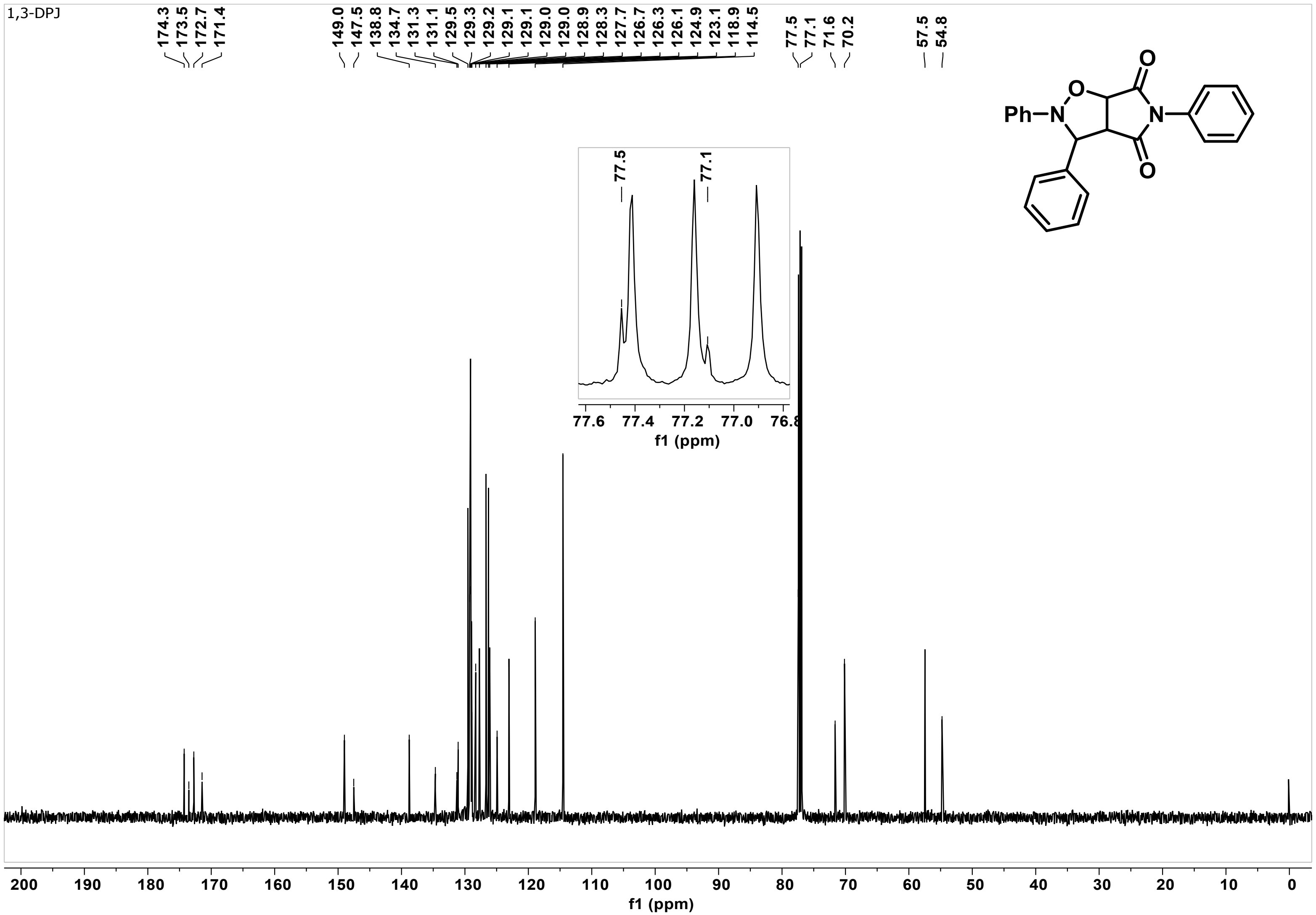


**Figure S14.** $^{13}C$ NMR spectrum of **3b** (125 MHz, $CDCl_3$).

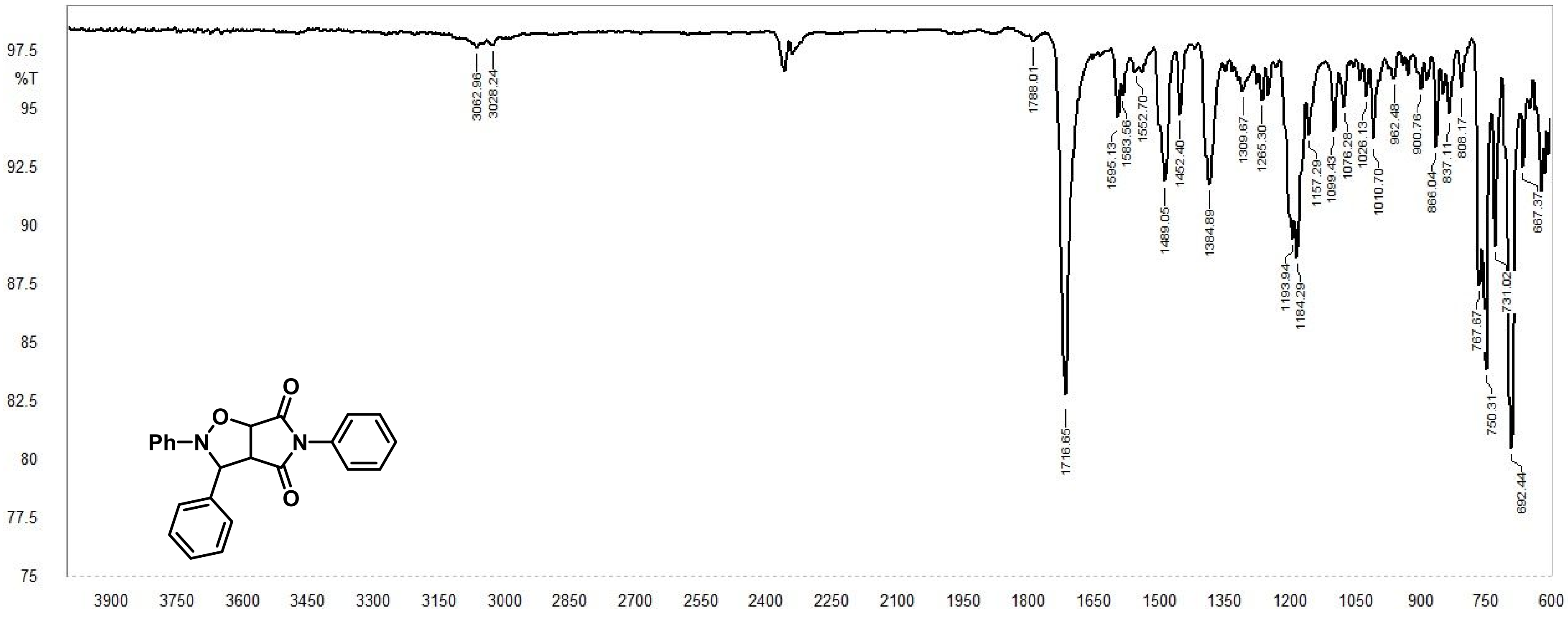


**Figure S15.** FT-IR spectrum of **3b**.

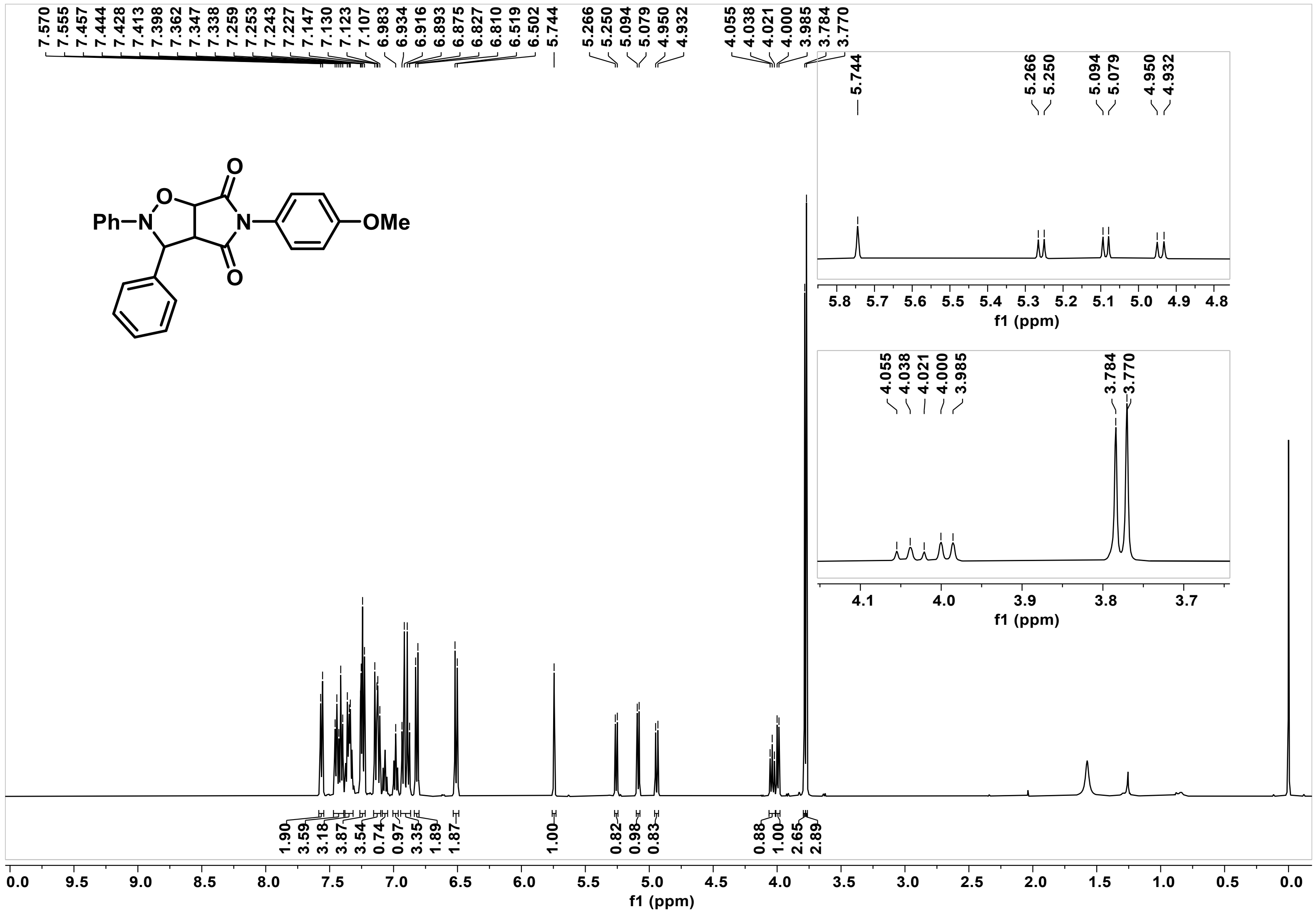

**Figure S16.** $^{1}$H NMR spectrum of **3c** (500 MHz, $CDCl_3$).

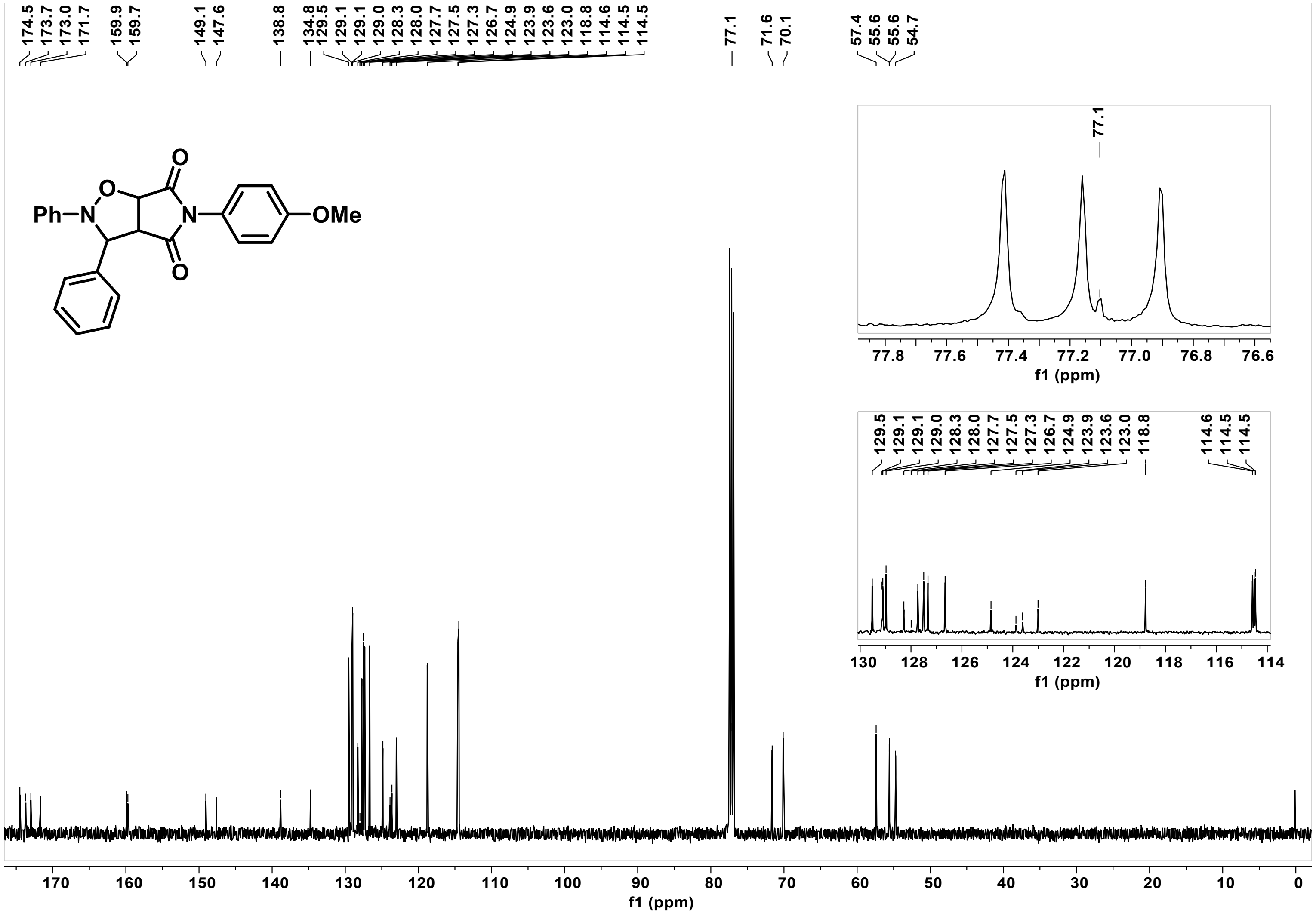

**Figure S17.** $^{13}C$ NMR spectrum of **3c** (125 MHz, $CDCl_3$).

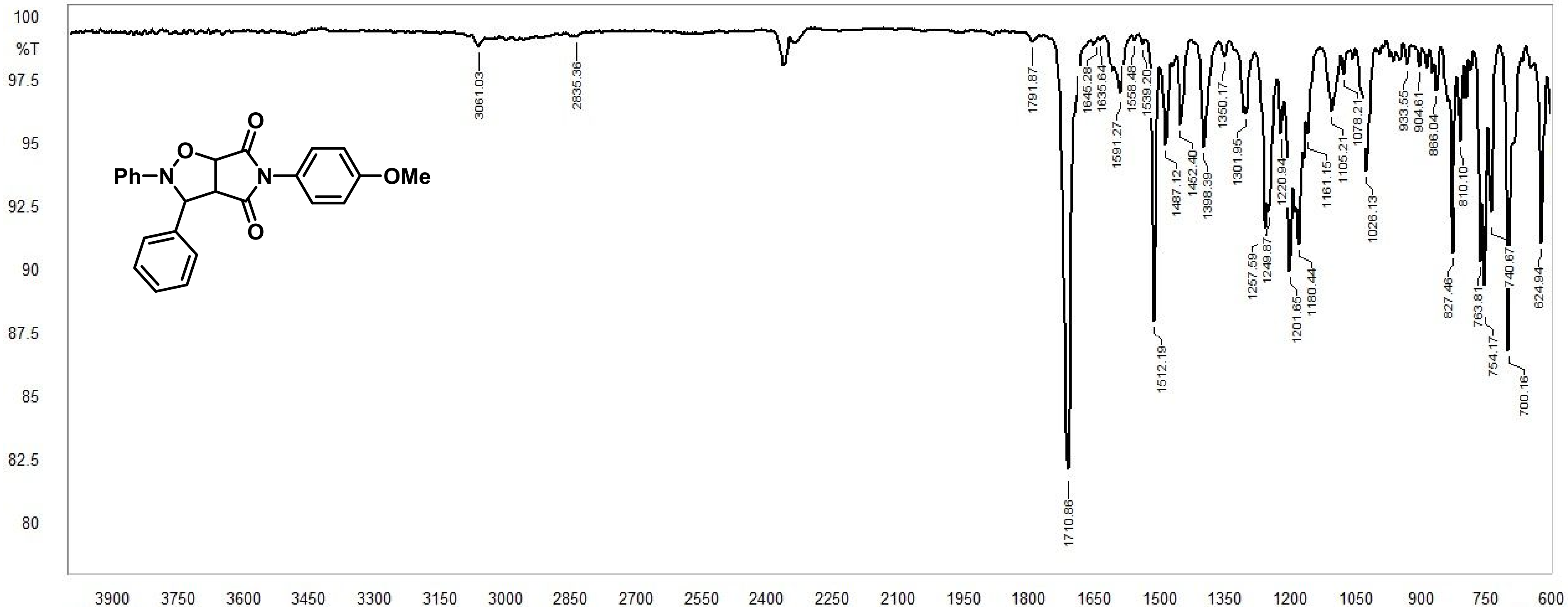

**Figure S18.** FT-IR spectrum of **3c**.

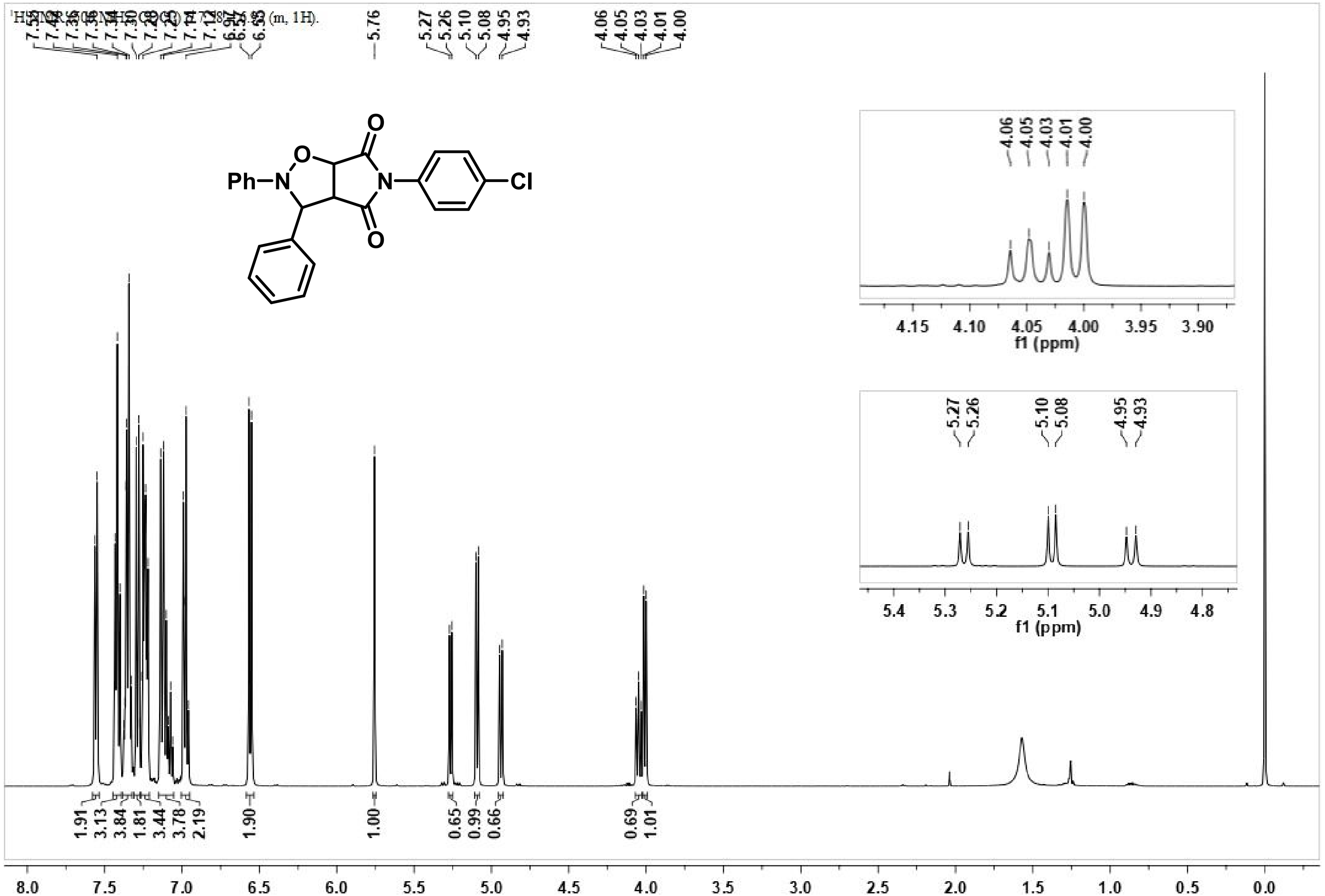

**Figure S19.** $^{1}H$ NMR spectrum of **3d** (500 MHz, $CDCl_3$).

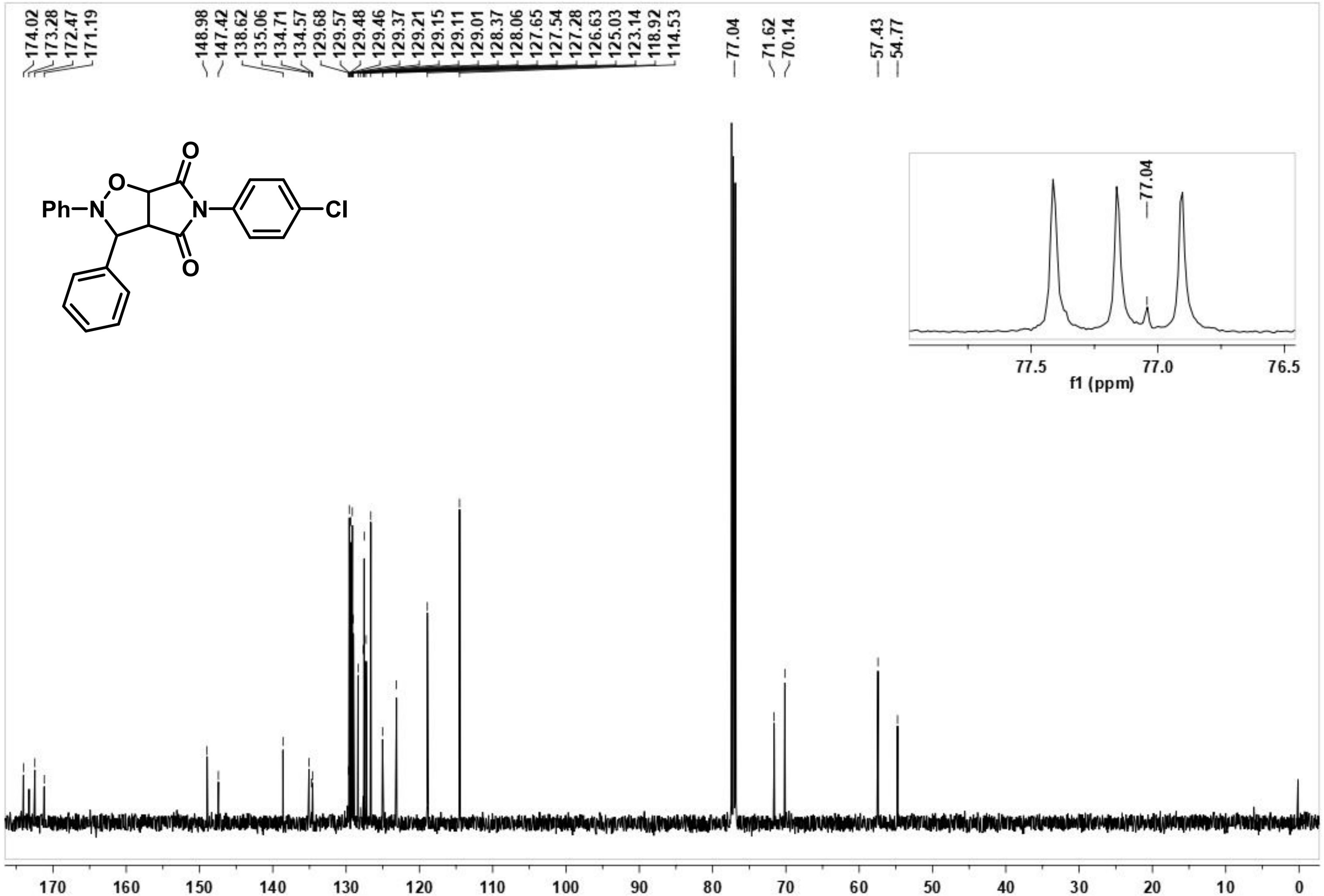

**Figure S20.** $^{13}C$ NMR spectrum of **3d** (125 MHz, $CDCl_3$).

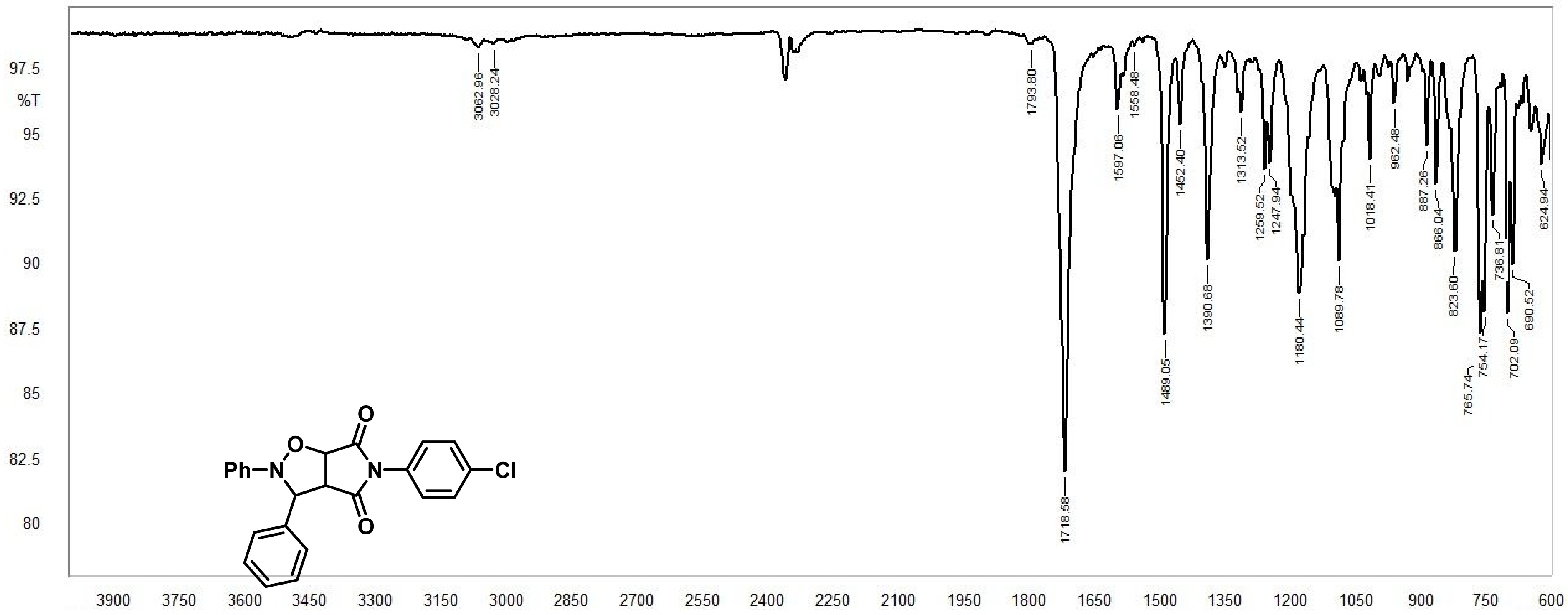


**Figure S21.** FT-IR spectrum of **3d**.

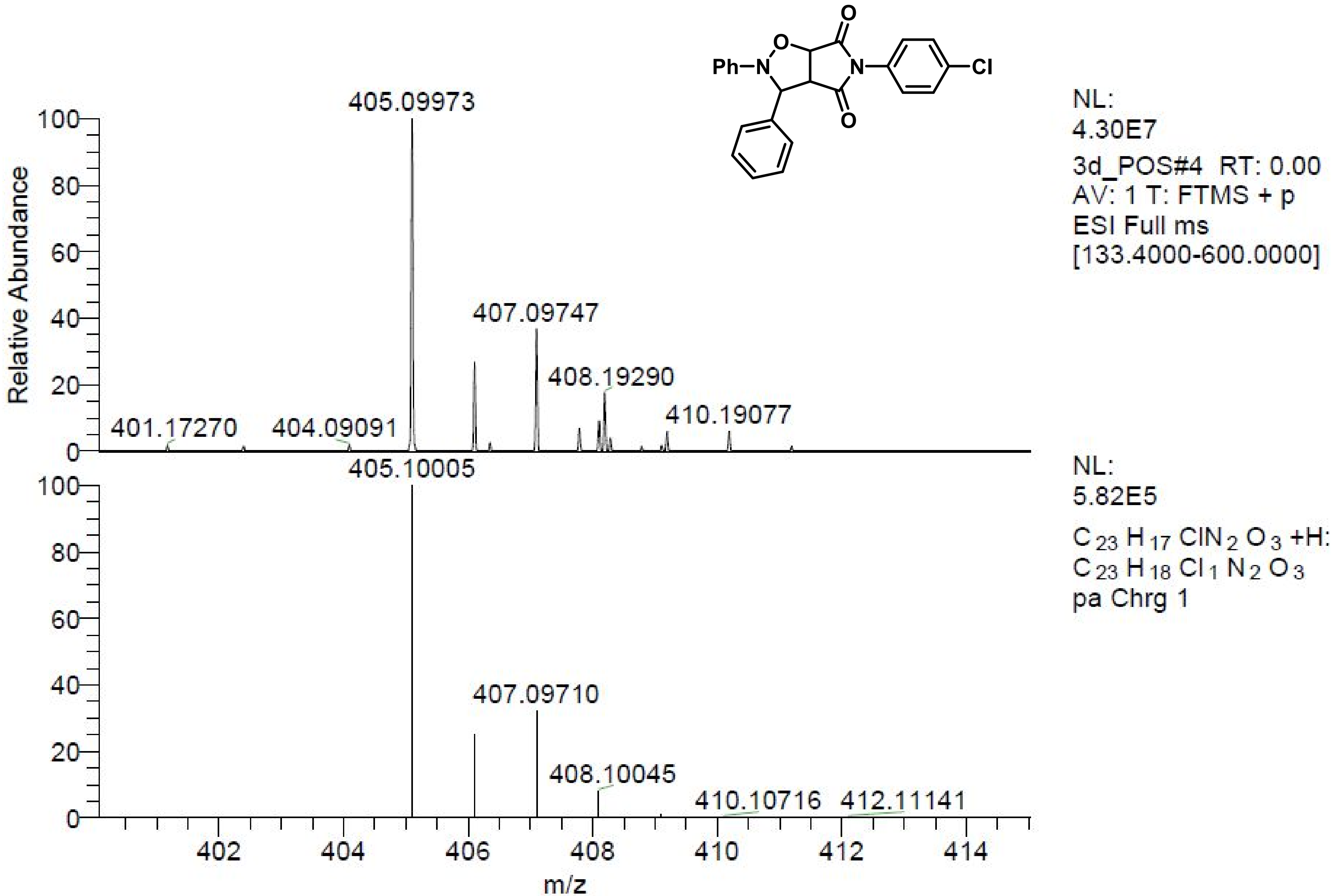


**Figure S22.** HRMS spectrum of **3d.**

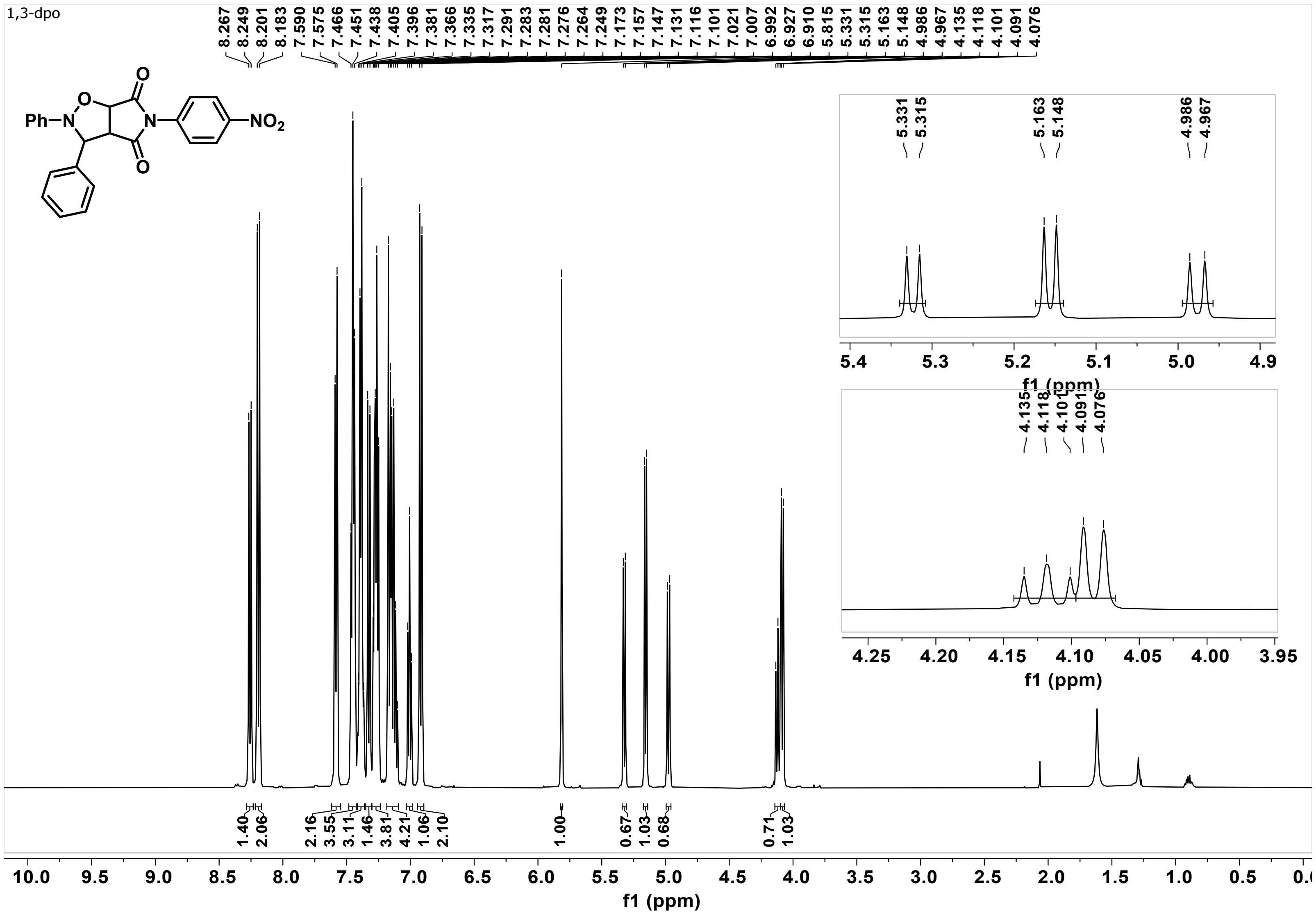


**Figure S23.** $^{1}$H NMR spectrum of **3e** (500 MHz, $CDCl_3$).

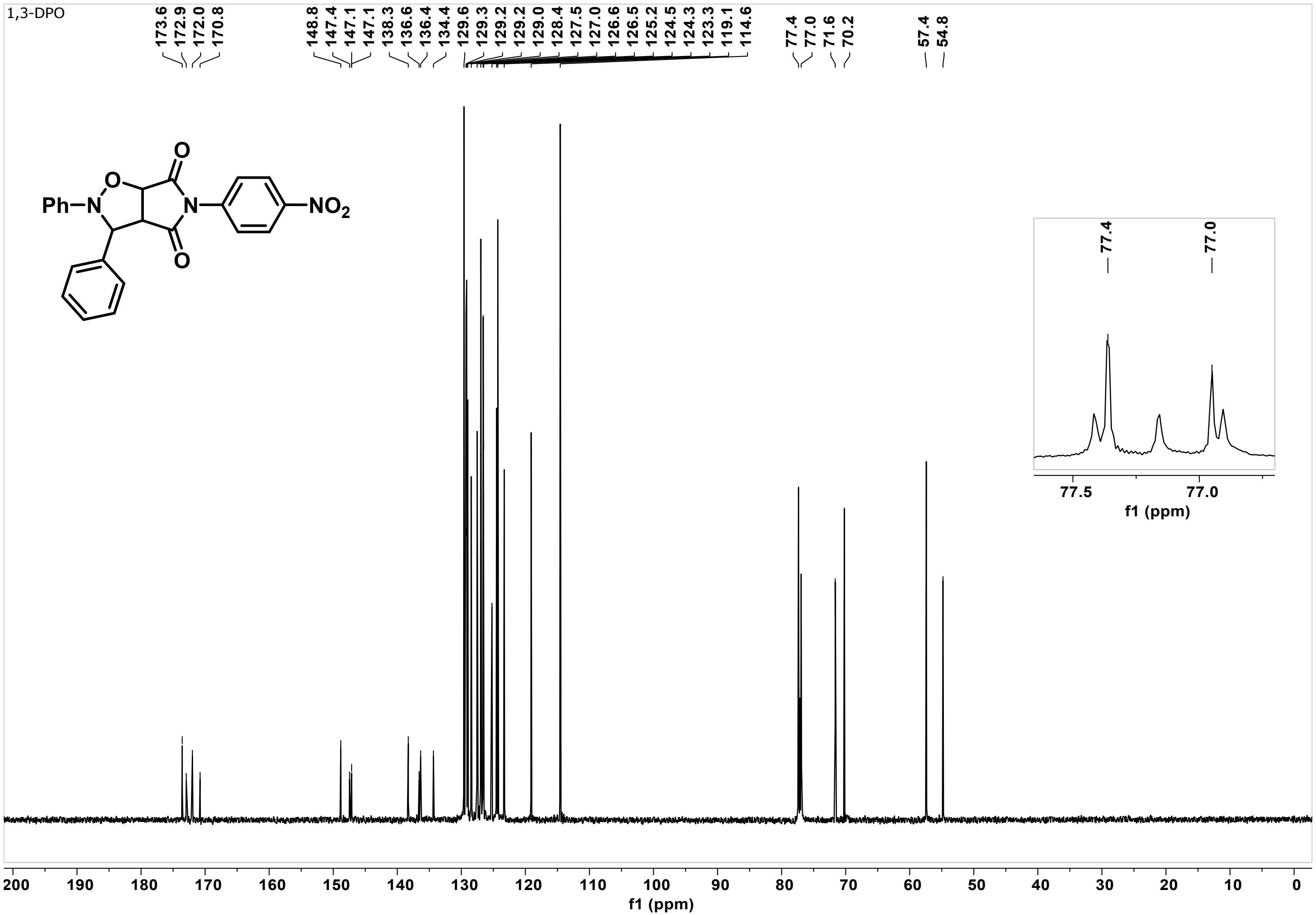


**Figure S24.** $^{13}$C NMR spectrum of **3e** (125 MHz, $CDCl_3$).

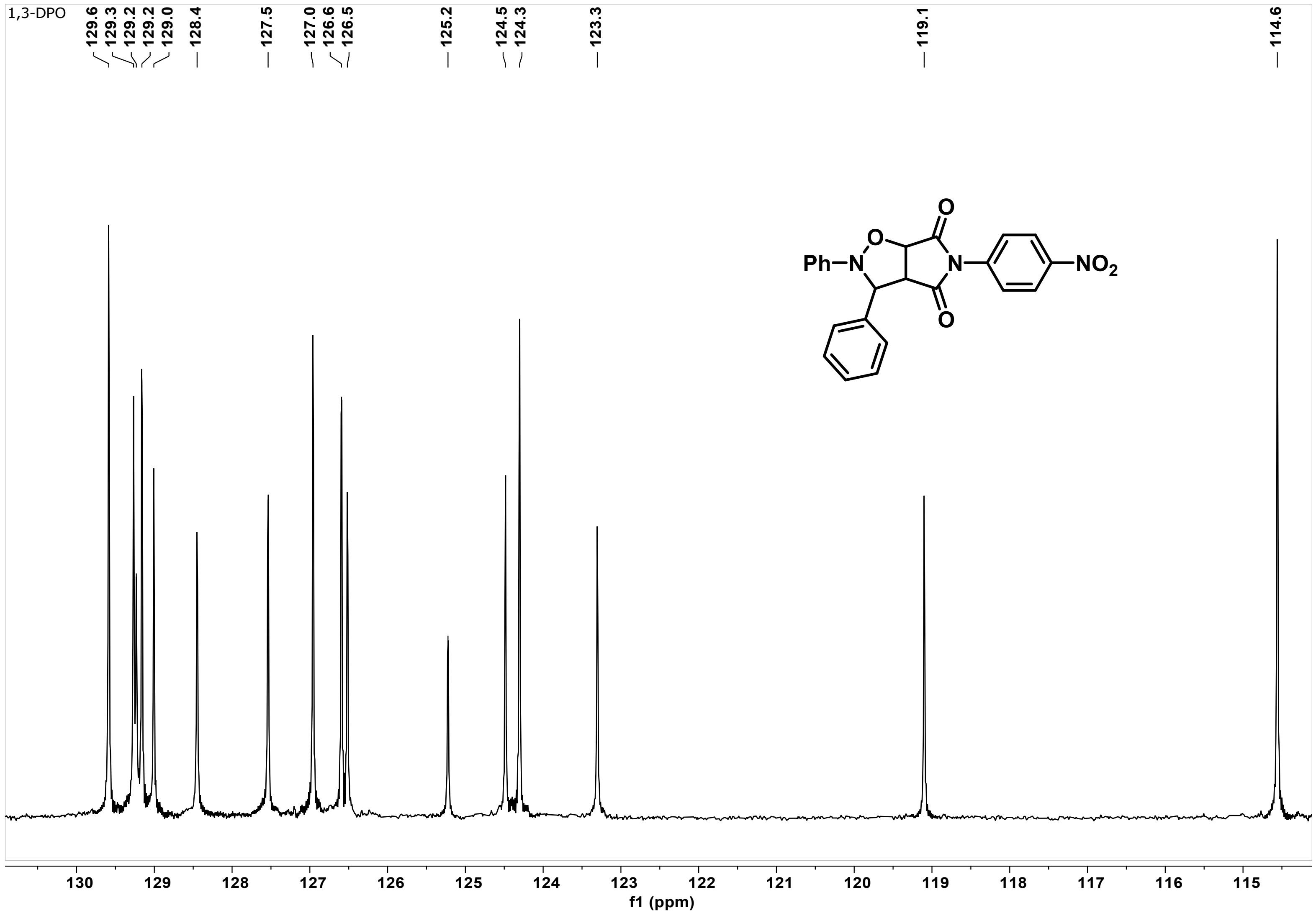


**Figure S25.** Expanded $^{13}C$ NMR spectrum of **3e** (125 MHz, $CDCl_3$).

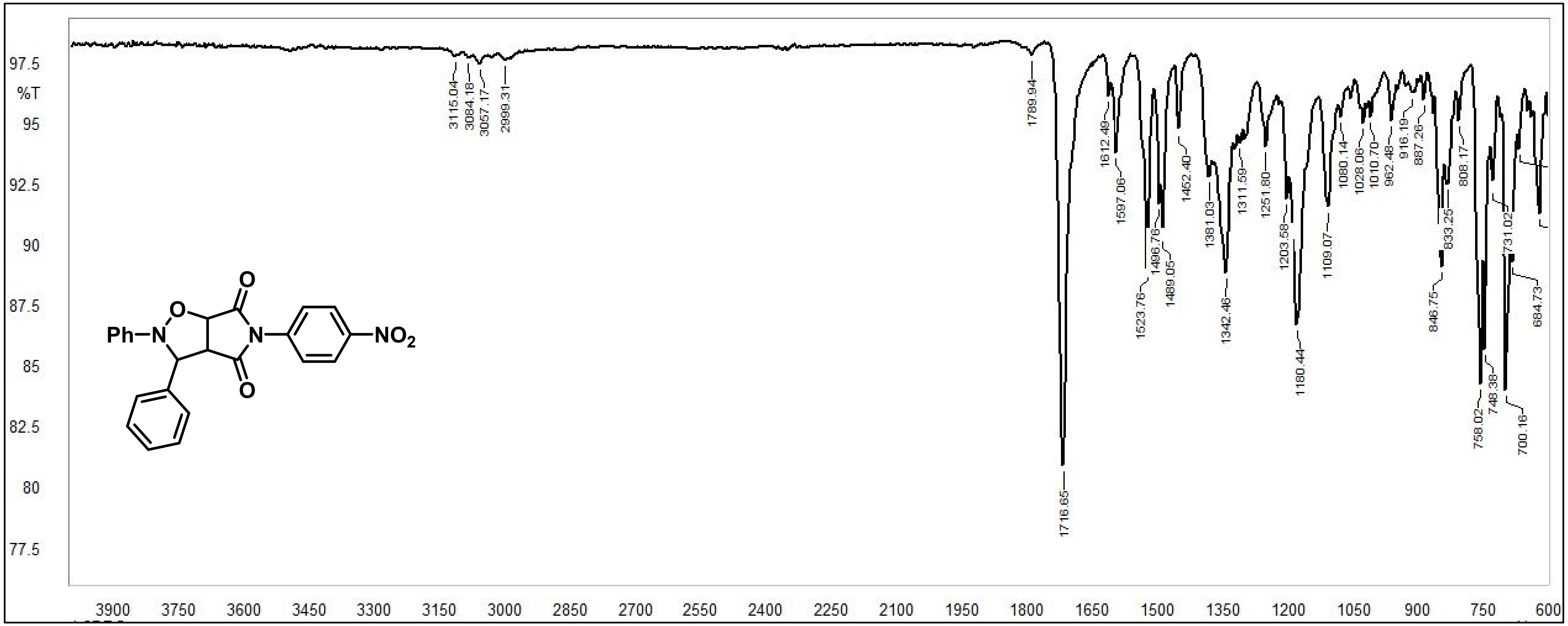


**Figure S26.** FT-IR spectrum of **3e**.

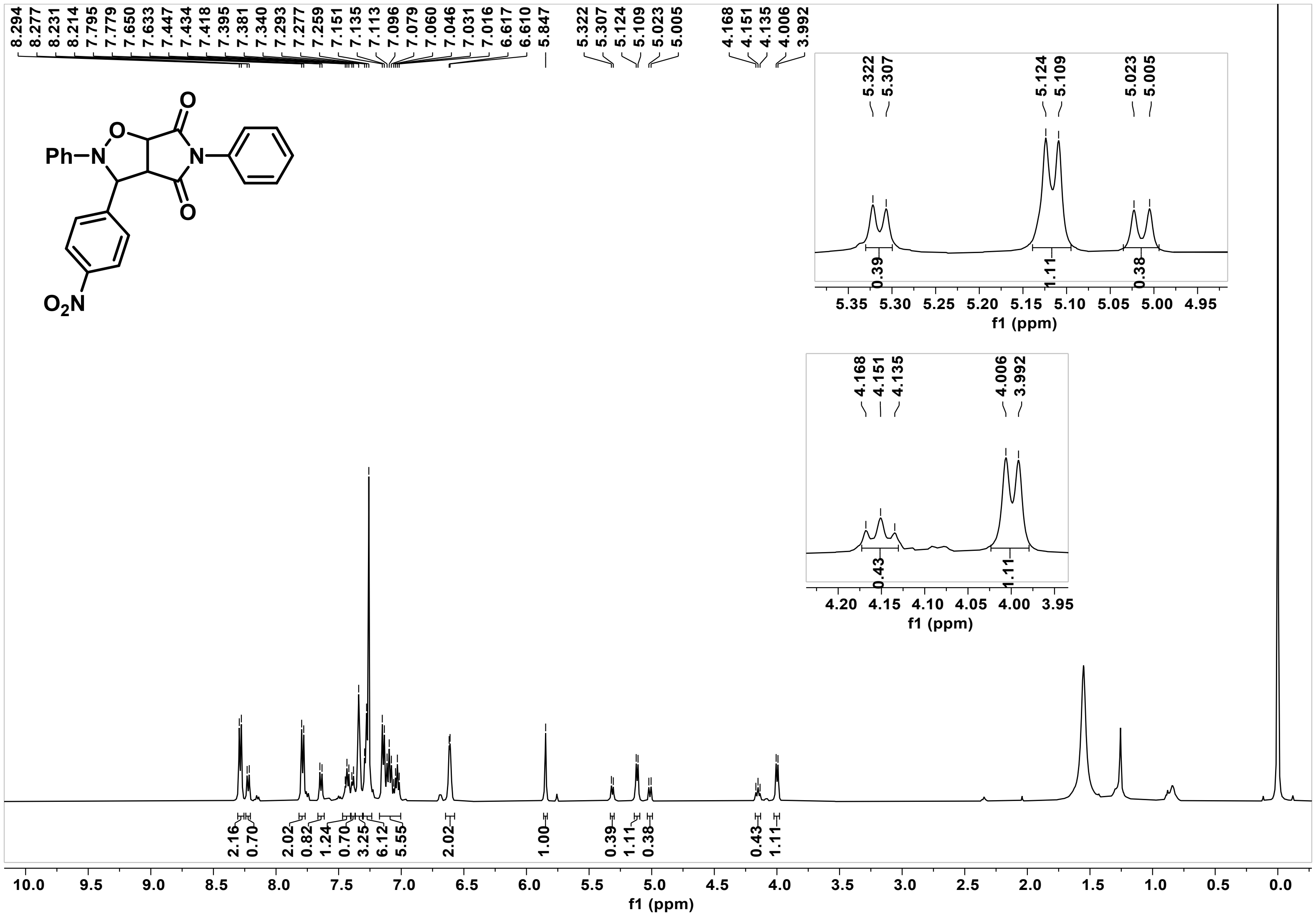

**Figure S27.** $^{1}$H NMR spectrum of **3f** (500 MHz, $CDCl_3$).

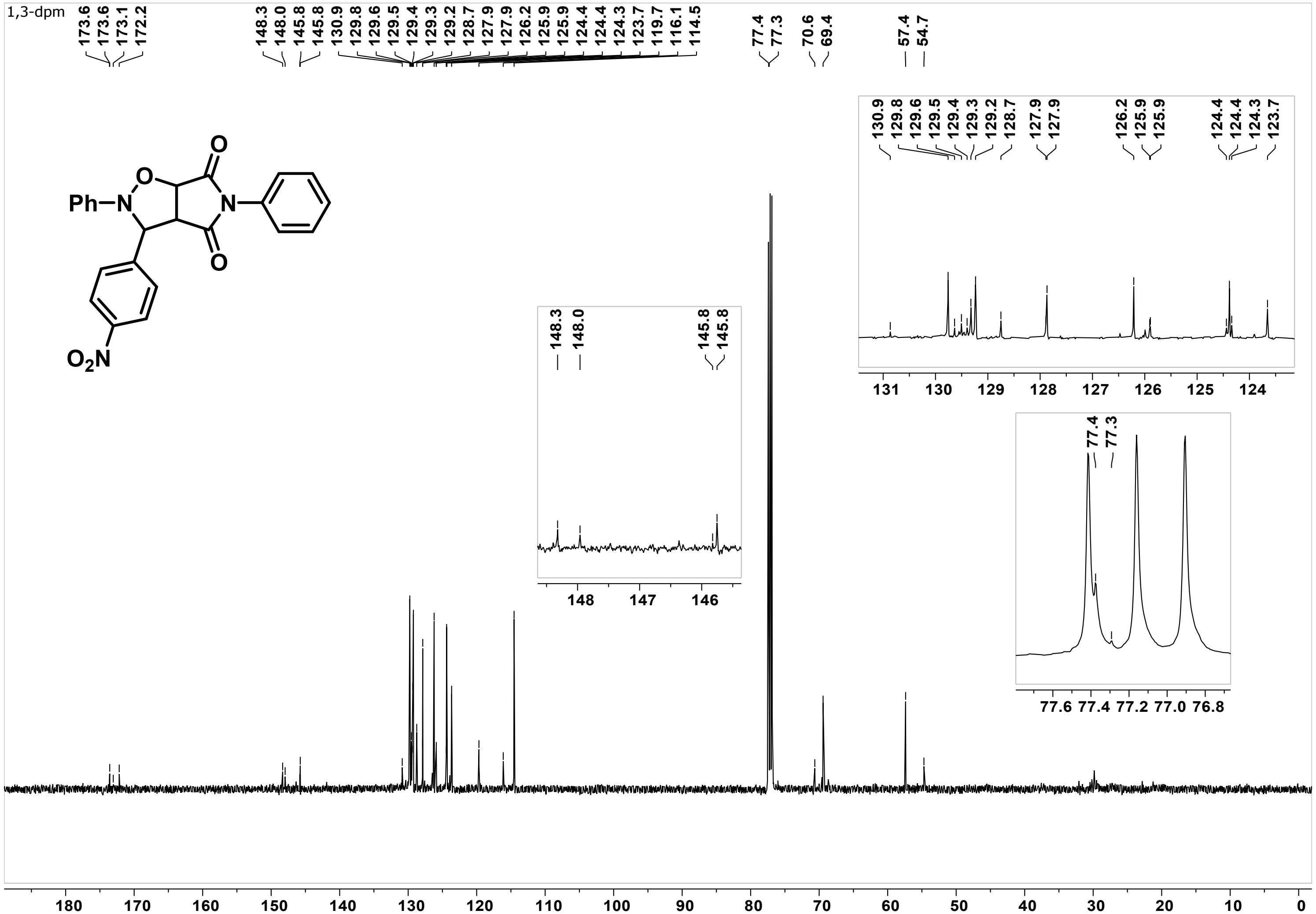


**Figure S28.** $^{13}$C NMR spectrum of **3f** (125 MHz, $CDCl_3$).

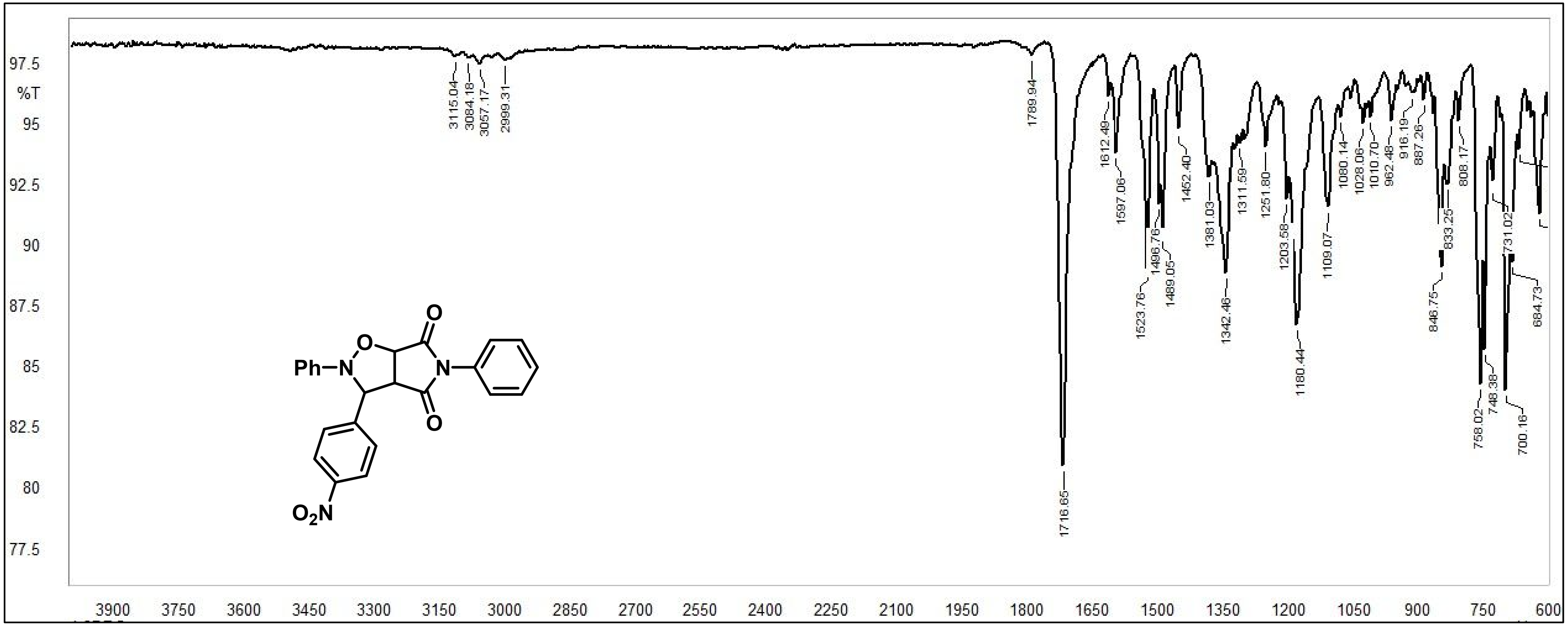


**Figure S29.** FT-IR spectrum of **3f**.

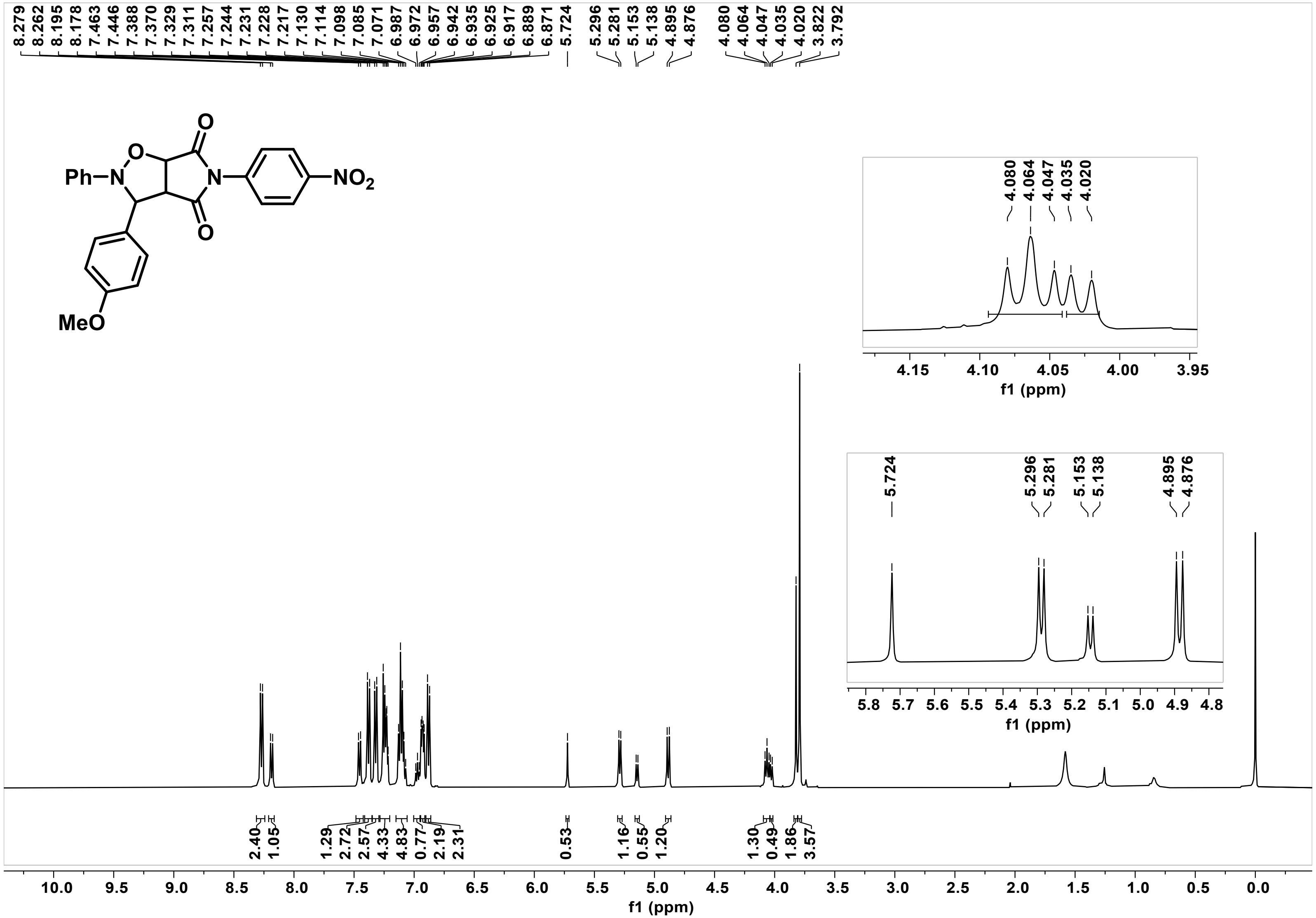

**Figure S30.** $^1$H NMR spectrum of **3g** (500 MHz, $CDCl_3$).

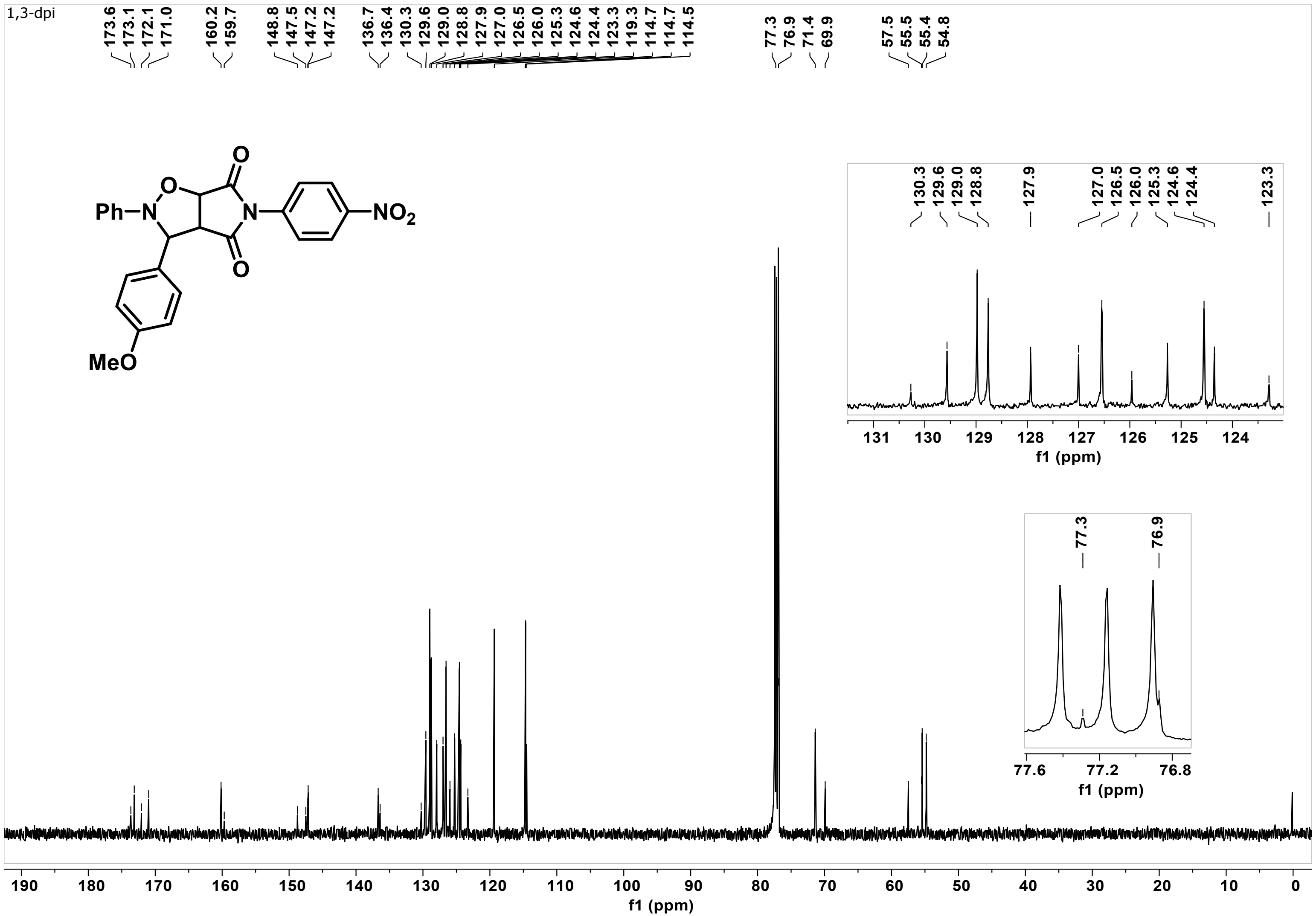


**Figure S31.** $^{13}$C NMR spectrum of **3g** (125 MHz, $CDCl_3$).

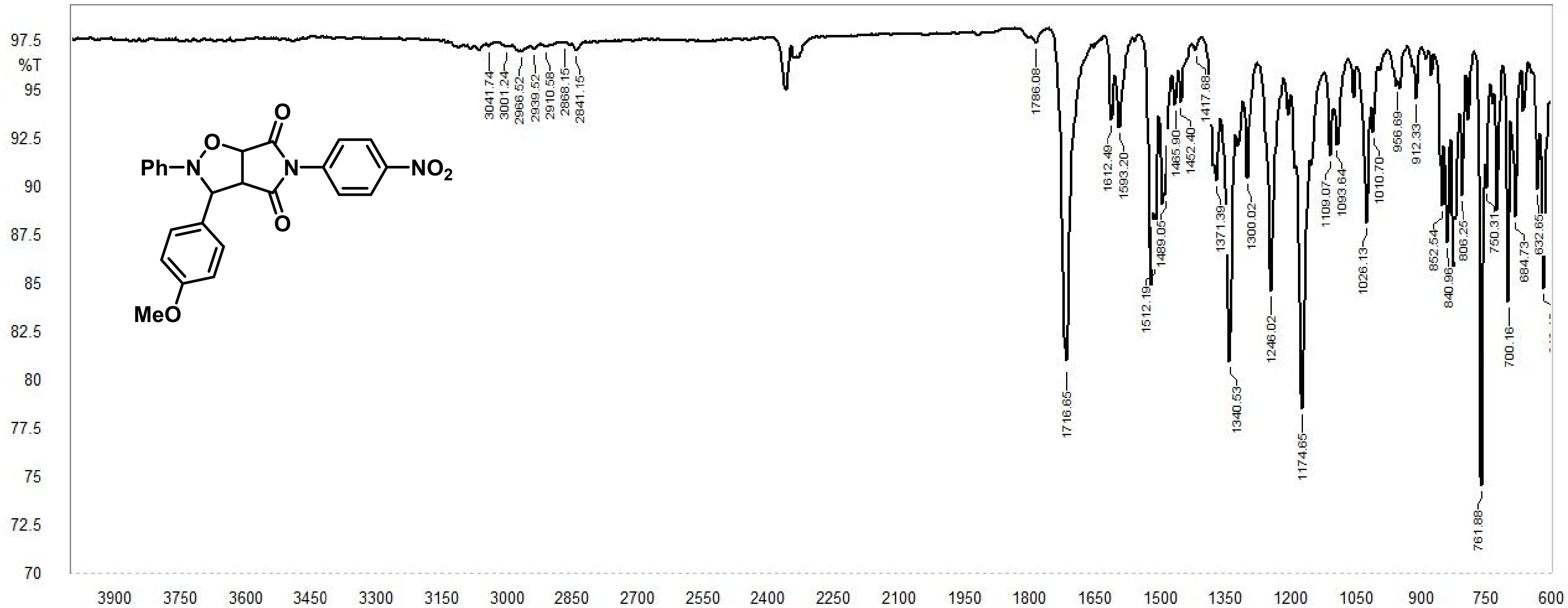

**Figure S32.** FT-IR spectrum of **3g**.

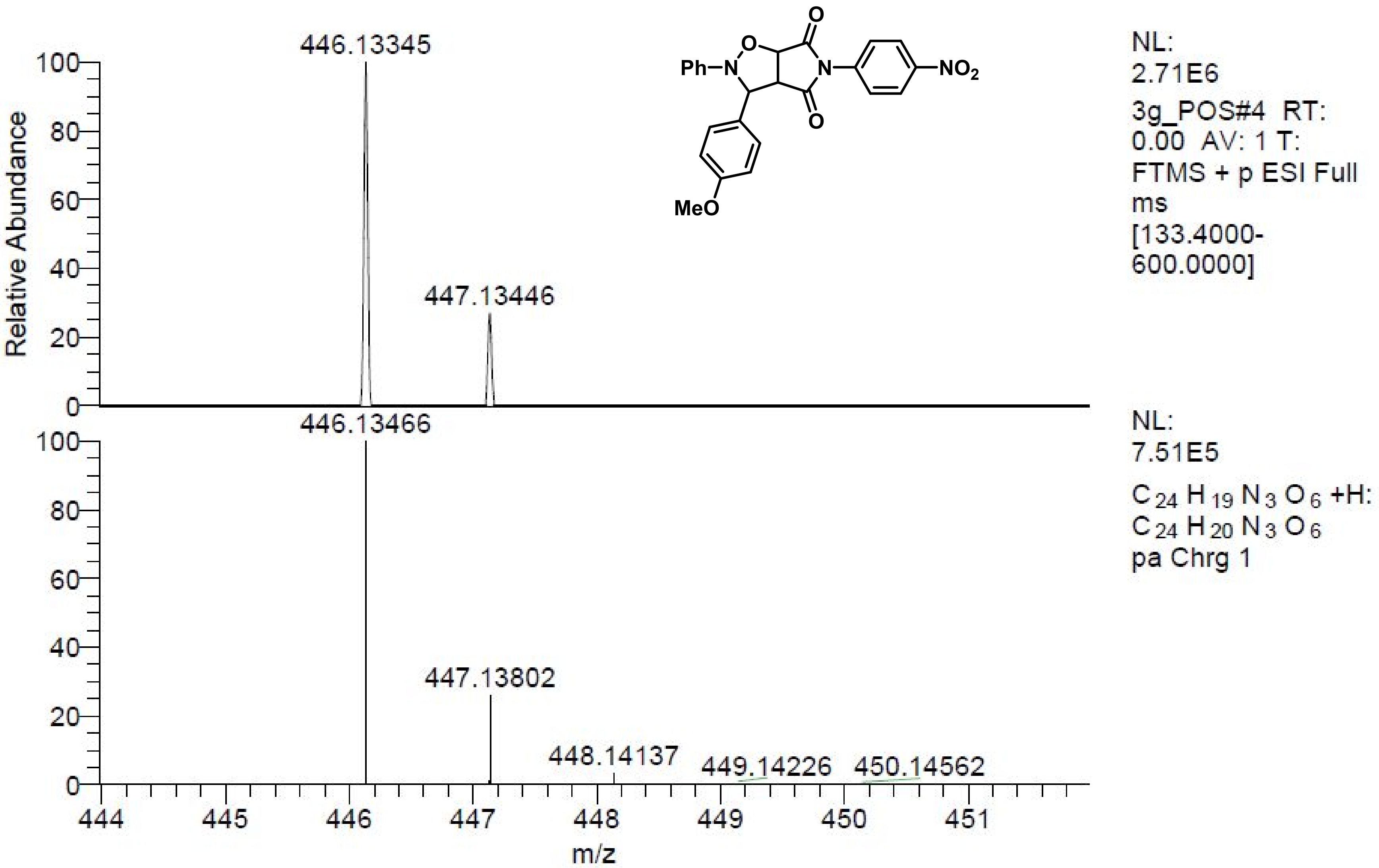


**Figure S33.** HRMS spectrum of **3g**.

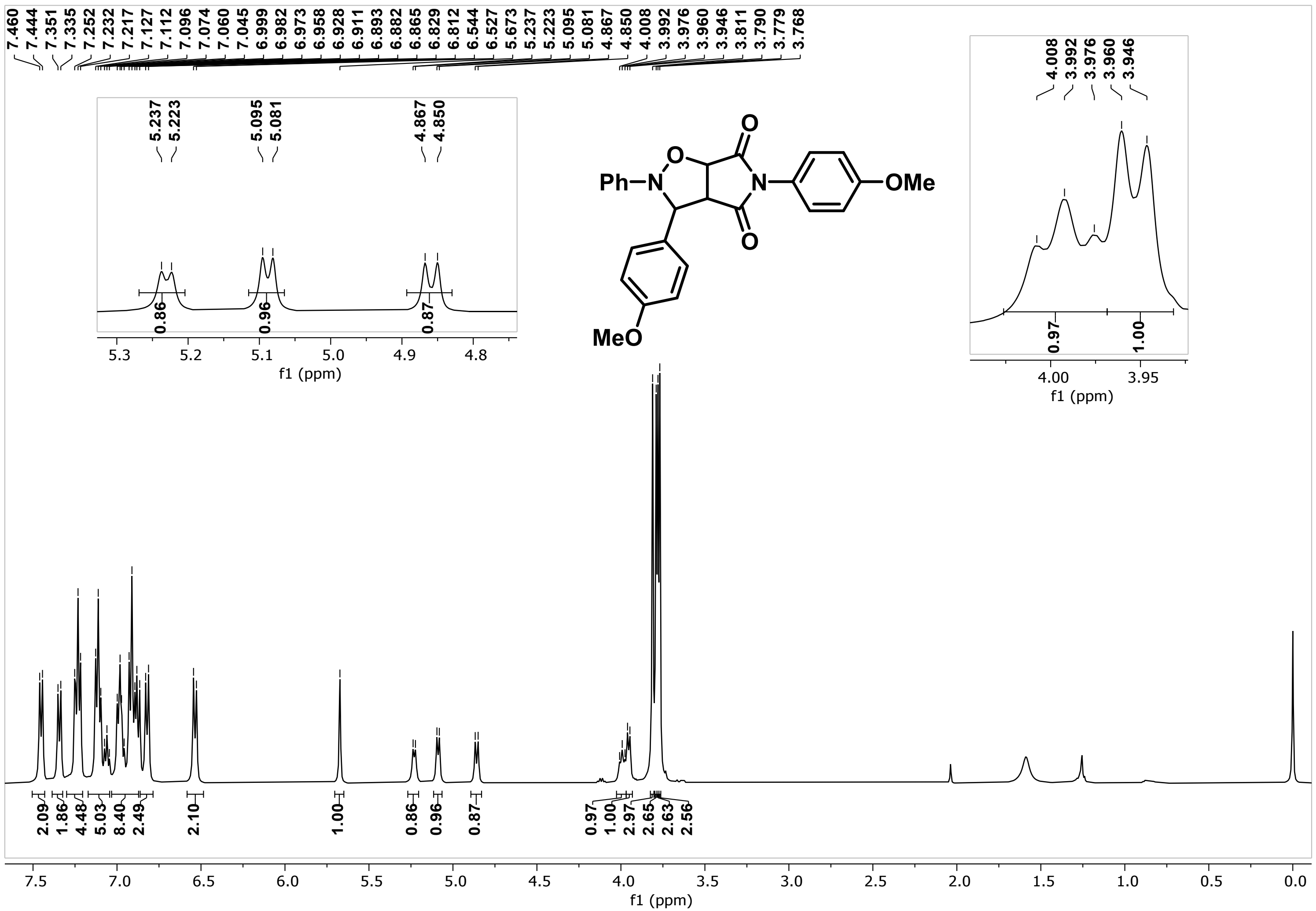

**Figure S34.** $^1$H NMR spectrum of **3h** (500 MHz, $CDCl_3$).

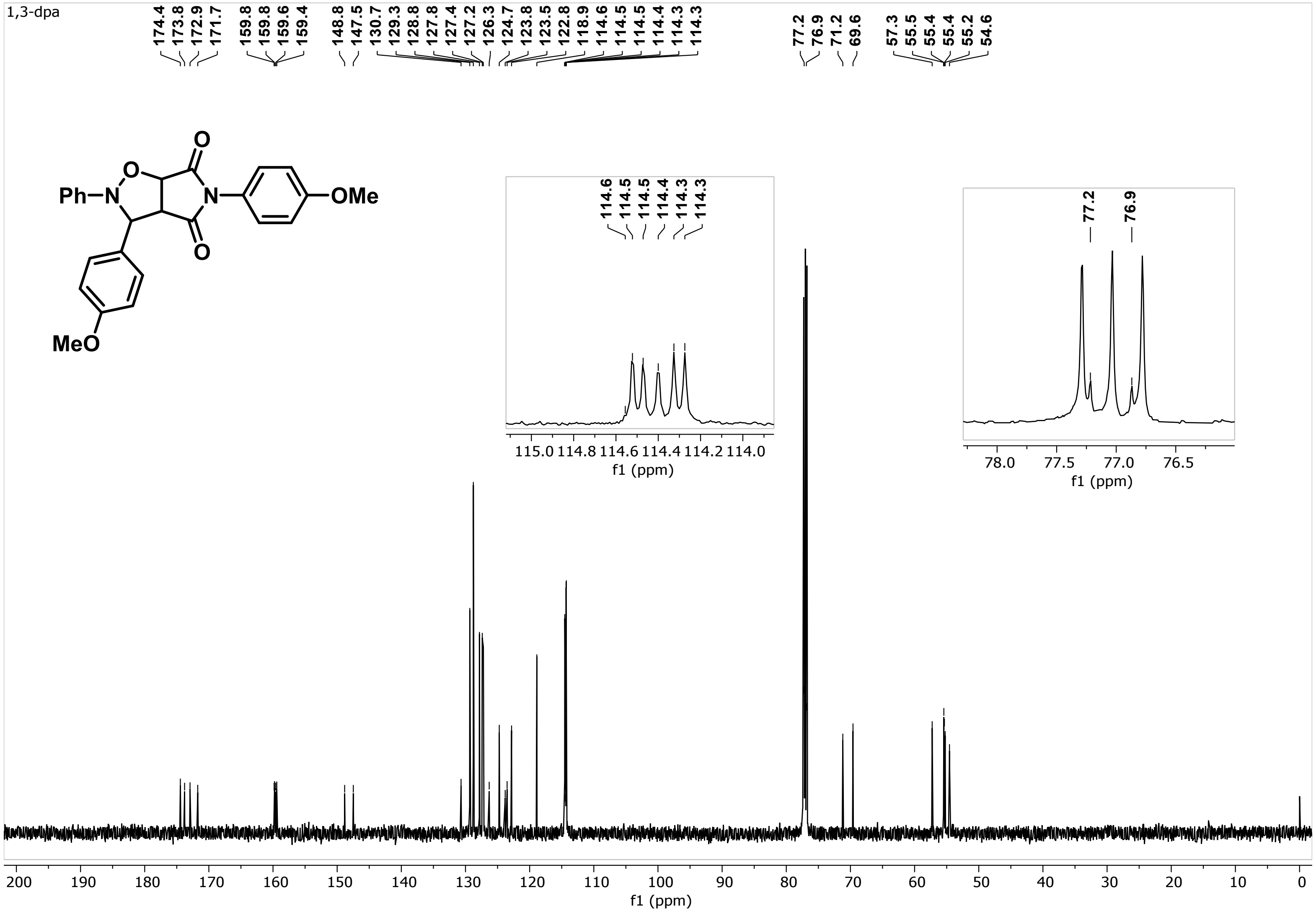


**Figure S35.** $^{13}$C NMR spectrum of **3h** (125 MHz, $CDCl_3$).

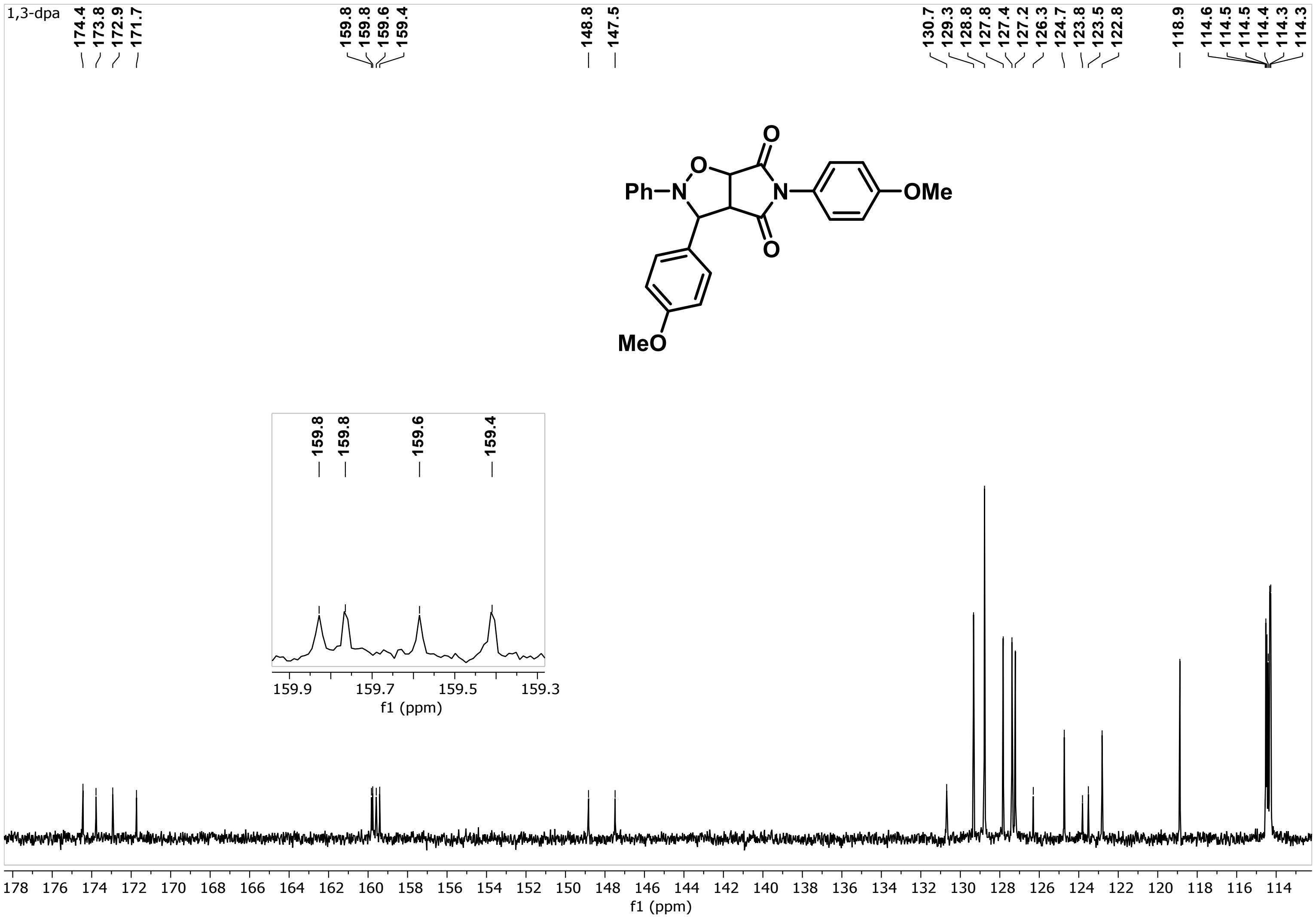


**Figure S36**. Expanded $^{13}$C NMR spectrum of **3h** (125 MHz, $CDCl_3$).

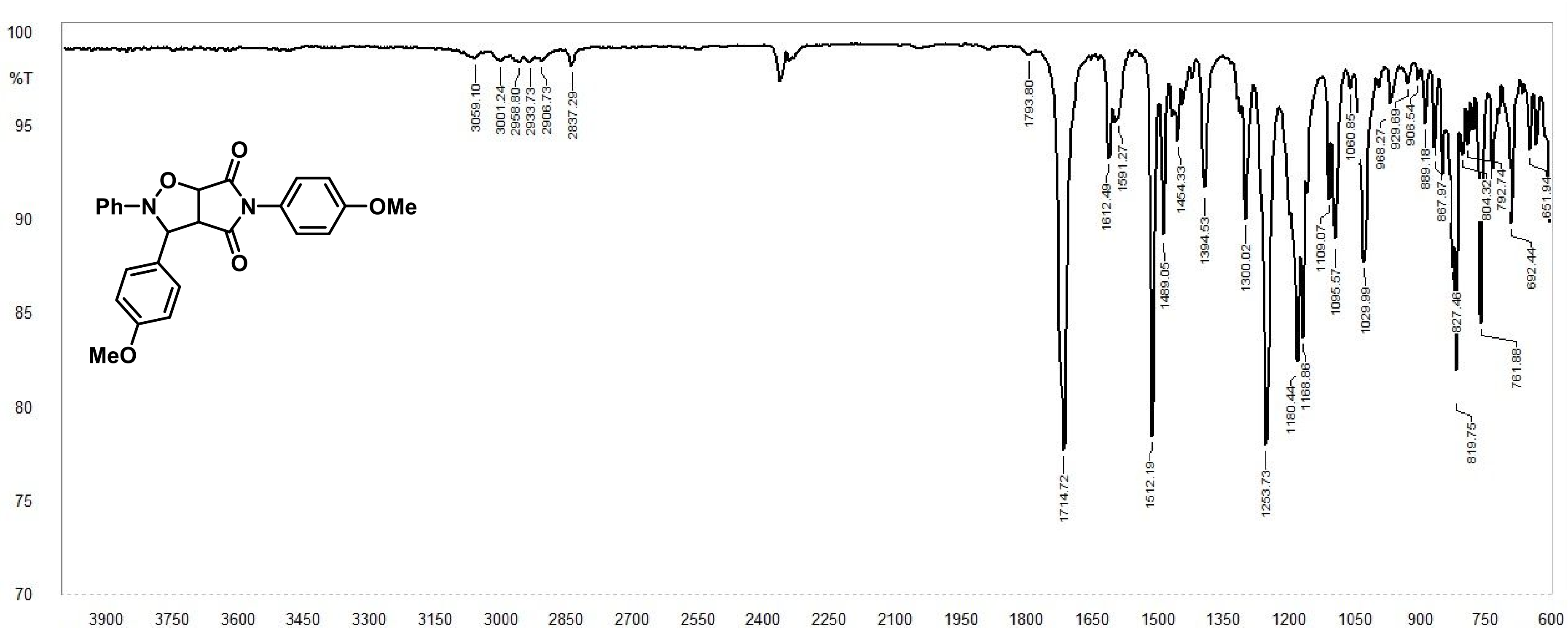


**Figure S37.** FT-IR spectrum of **3h**.

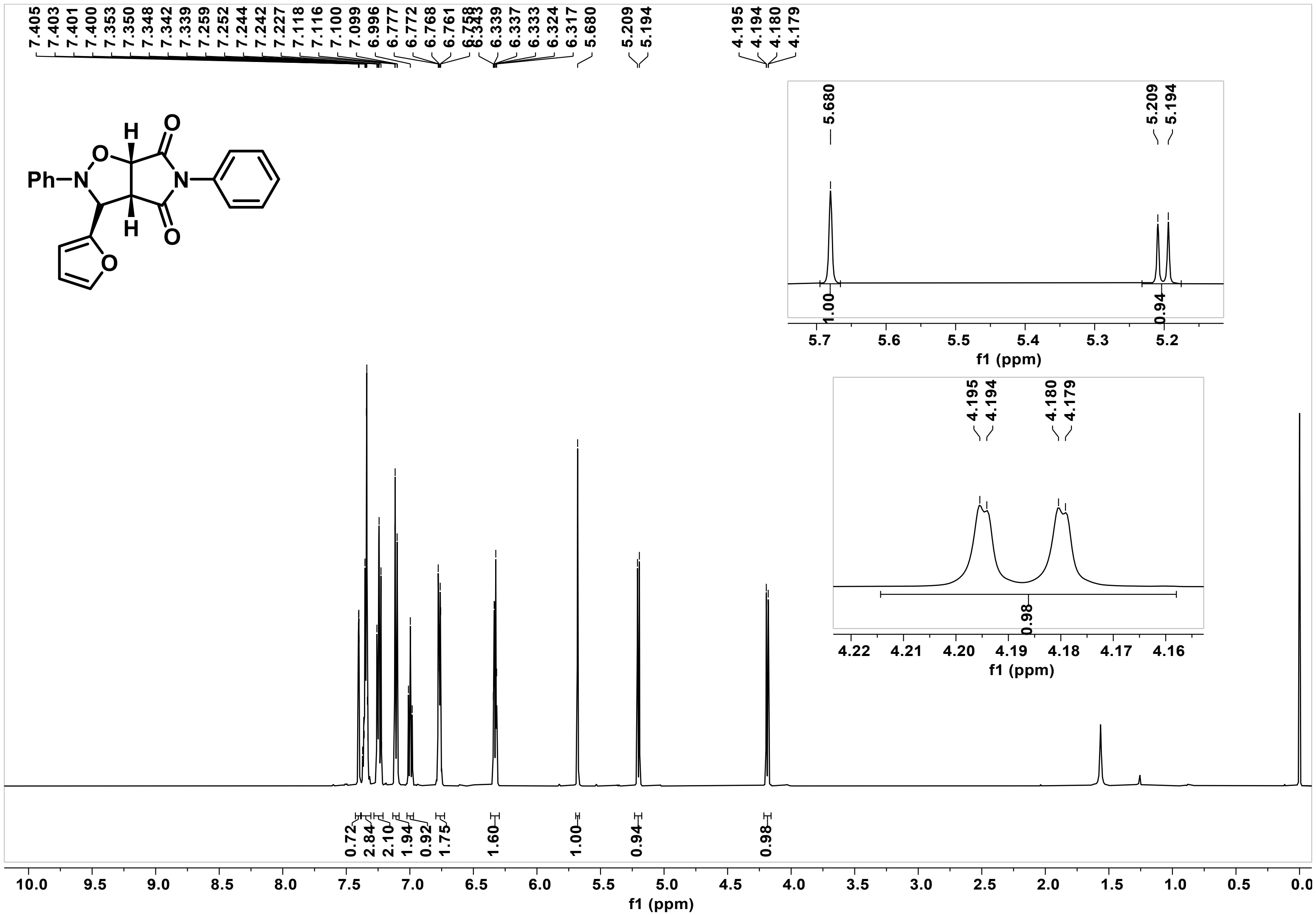

**Figure S38.** $^{1}$H NMR spectrum of **3i** ***(trans isomer)*** (500 MHz, $CDCl_3$).

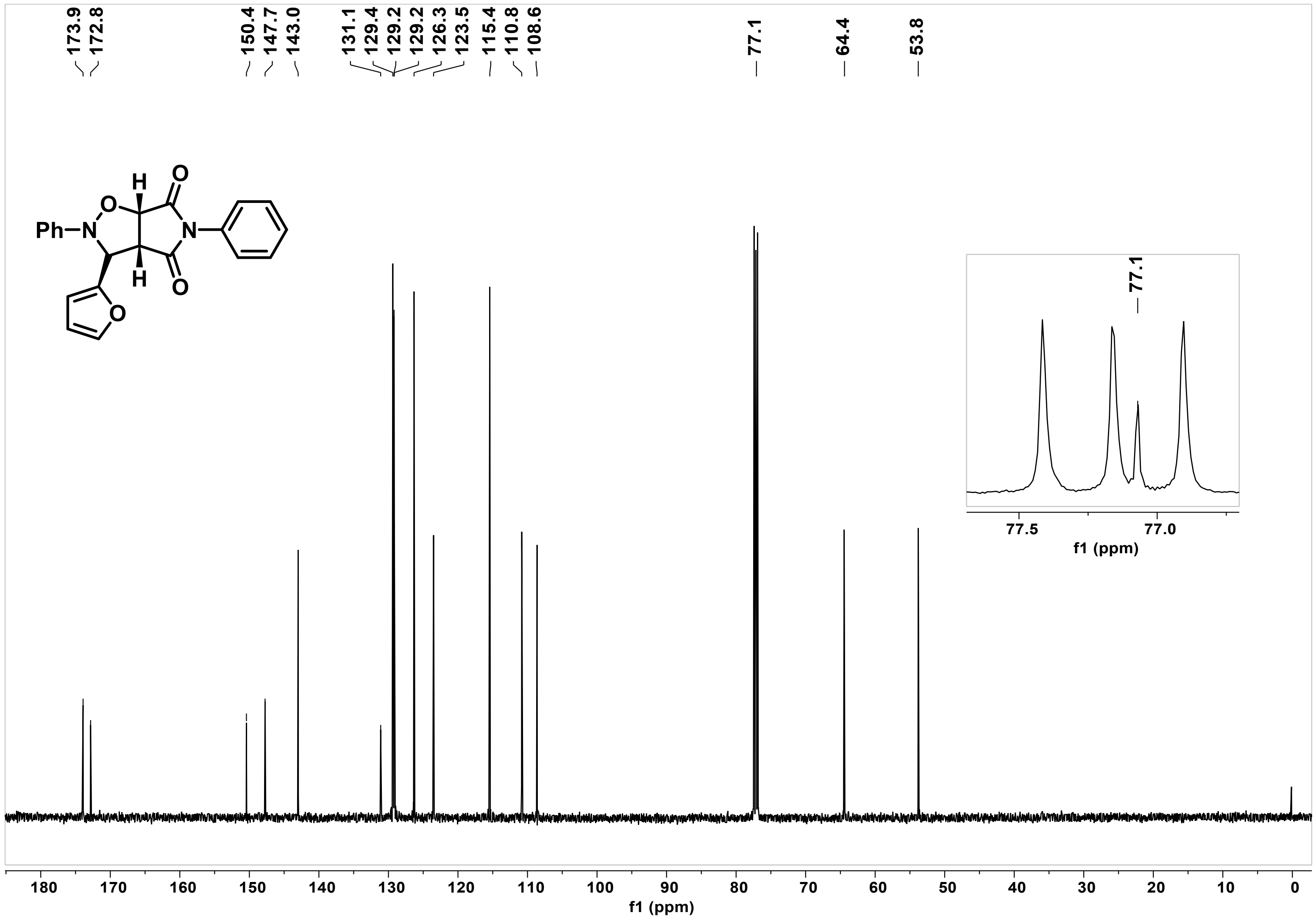


**Figure S39.** $^{13}$C NMR spectrum of **3i** ***(trans isomer)*** (125 MHz, $CDCl_3$).

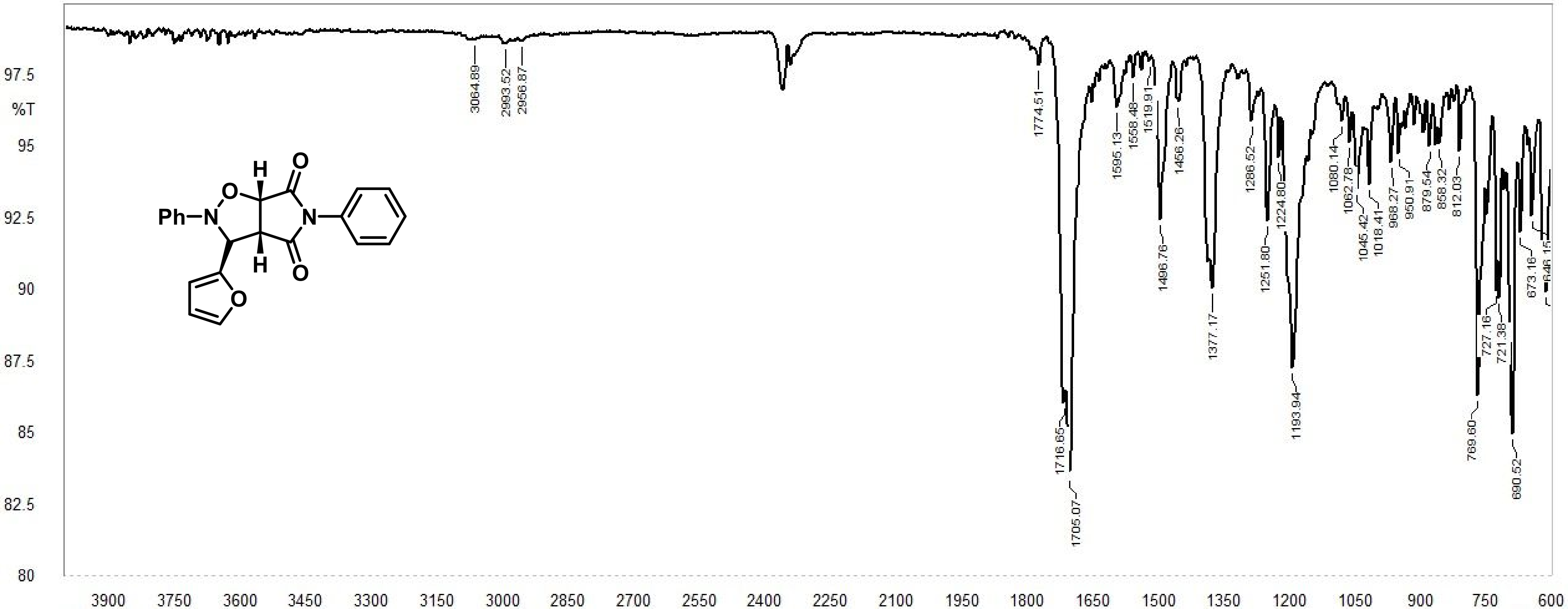


**Figure S40.** FT-IR spectrum of **3i** ***(trans isomer)***.

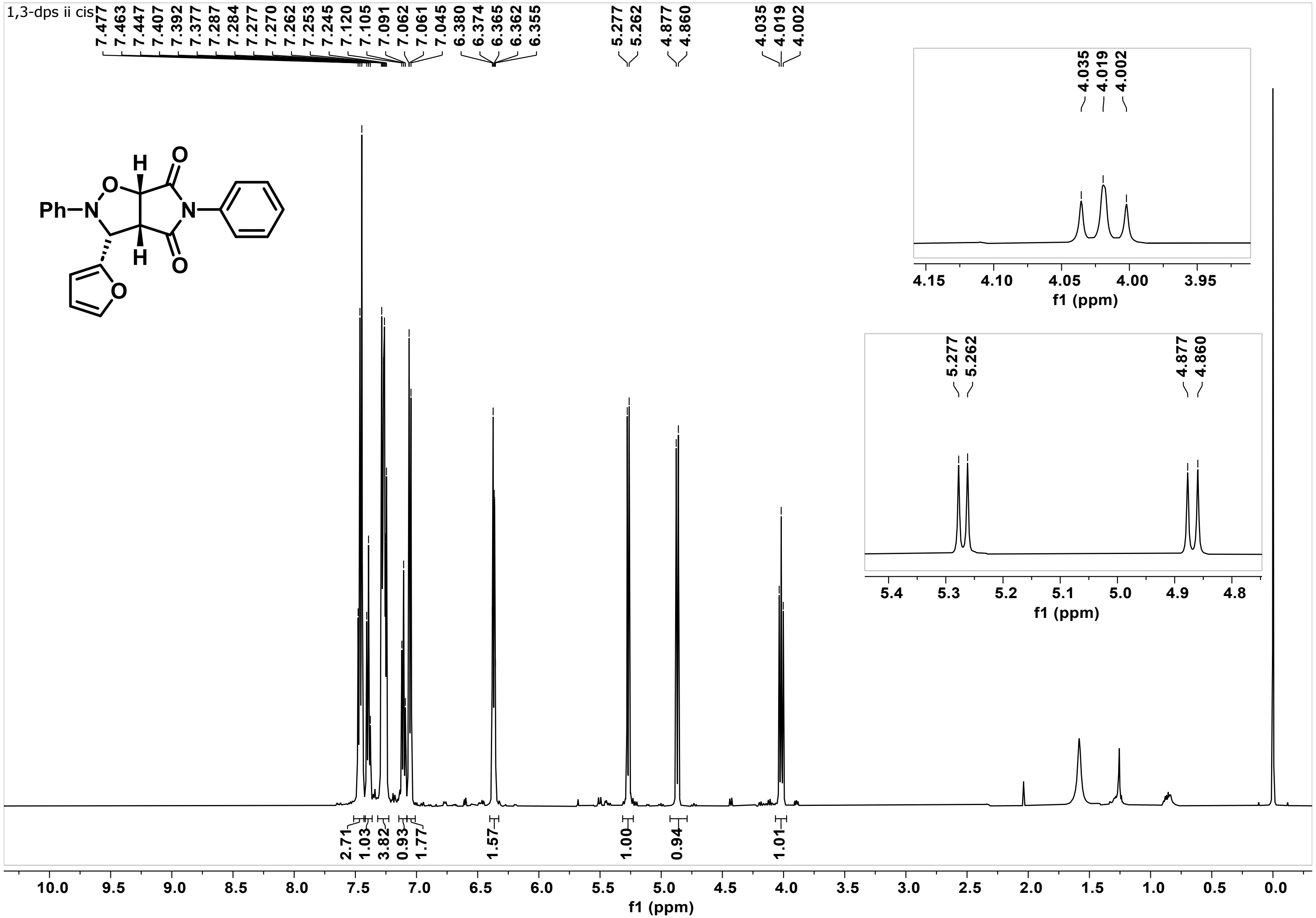


**Figure S41.** $^1$H NMR spectrum of **3i** ***(cis isomer)*** (500 MHz, $CDCl_3$).

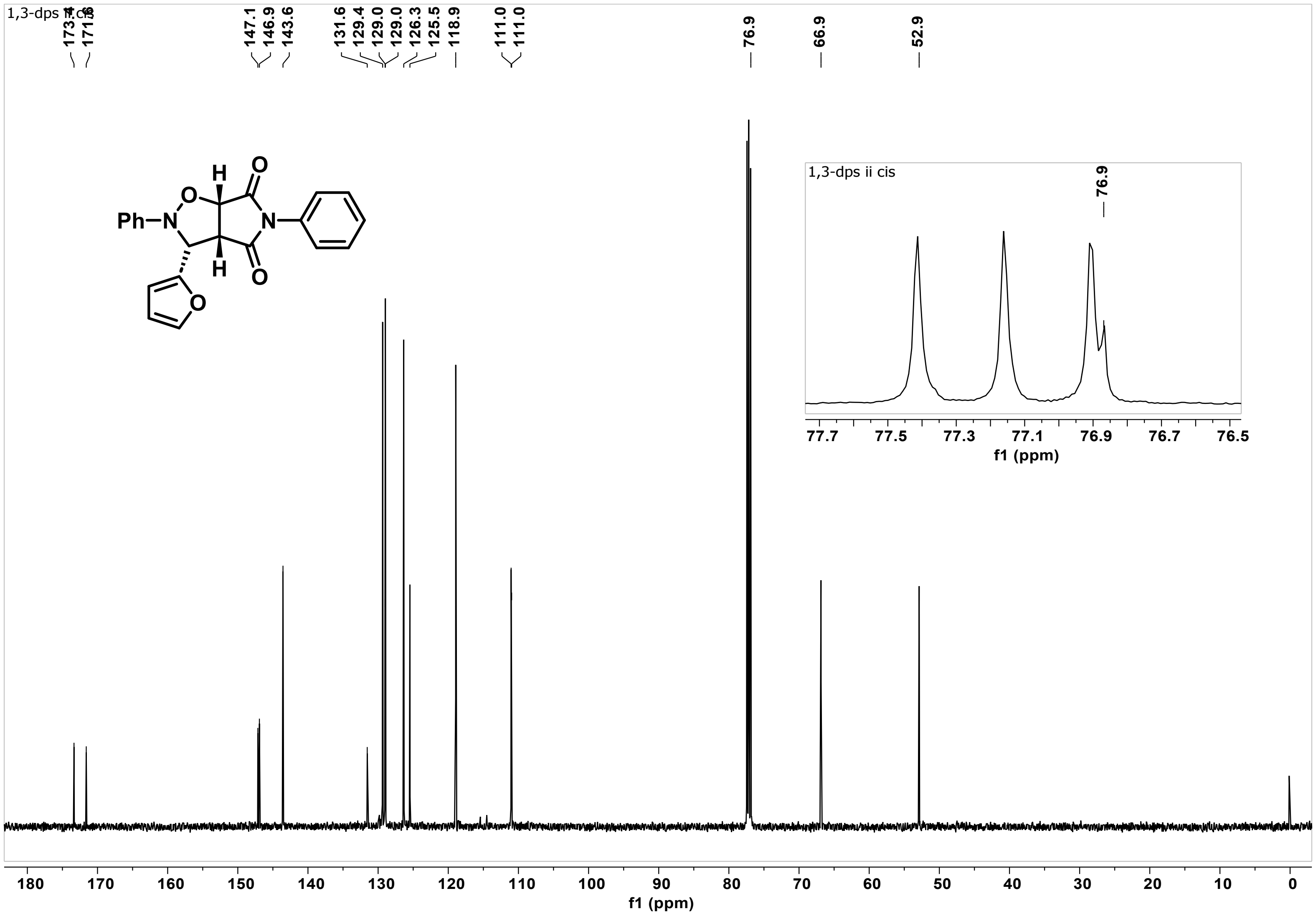


**Figure S42.** $^{13}$C NMR spectrum of **3i** ***(cis isomer)*** (125 MHz, $CDCl_3$).

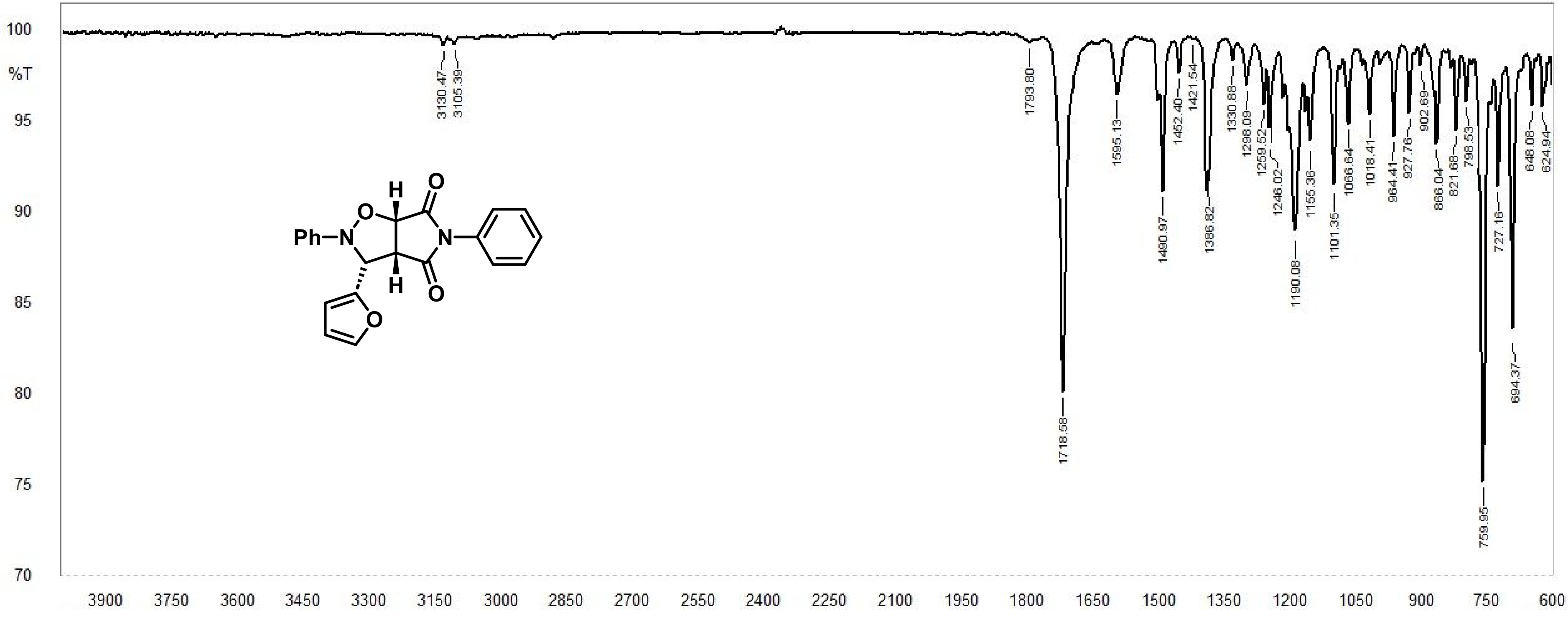


**Figure S43.** FT-IR spectrum of **3i** ***(cis isomer)***.

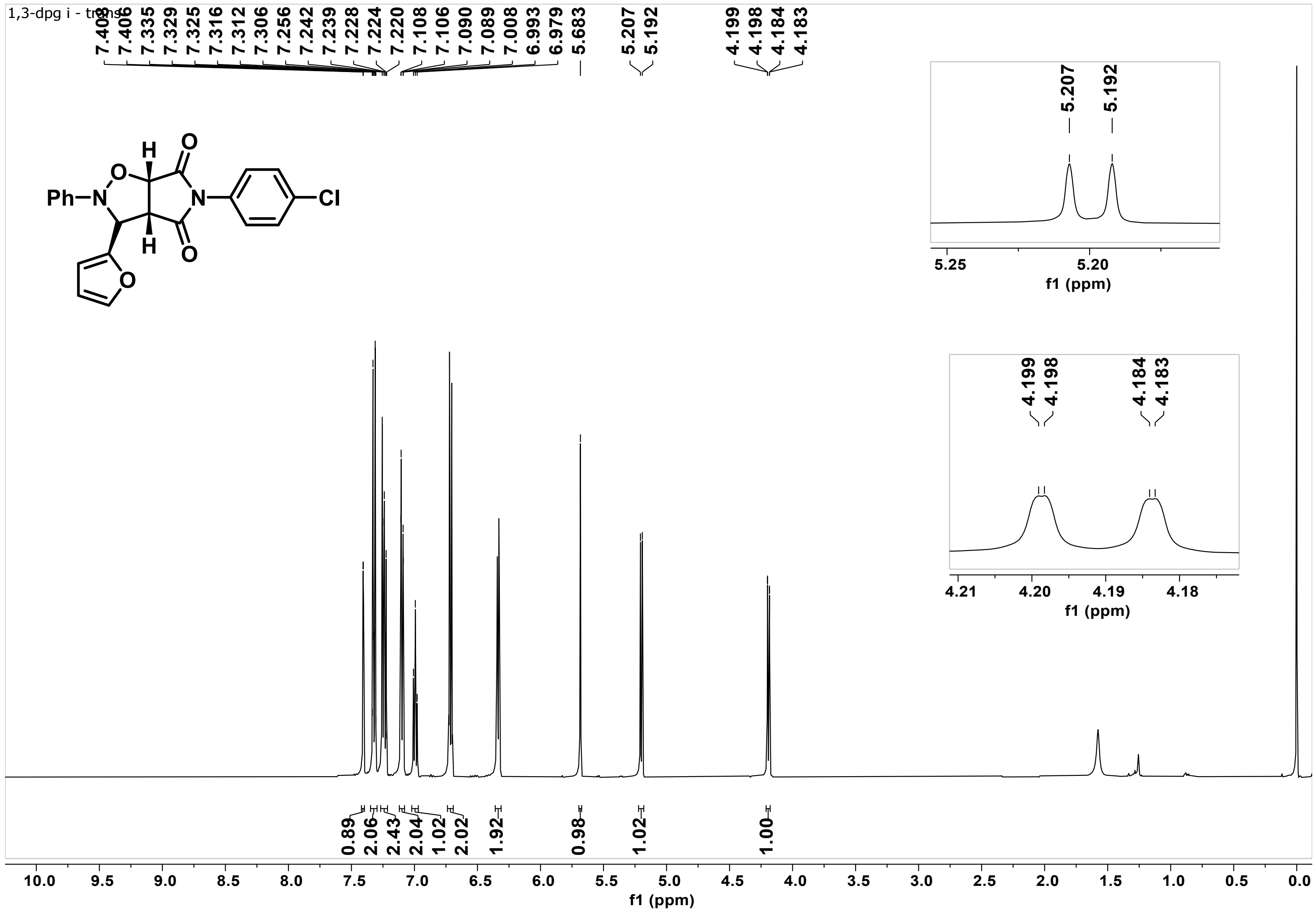


**Figure S44.** $^1$H NMR spectrum of **3j** ***(trans isomer)*** (500 MHz, $CDCl_3$).

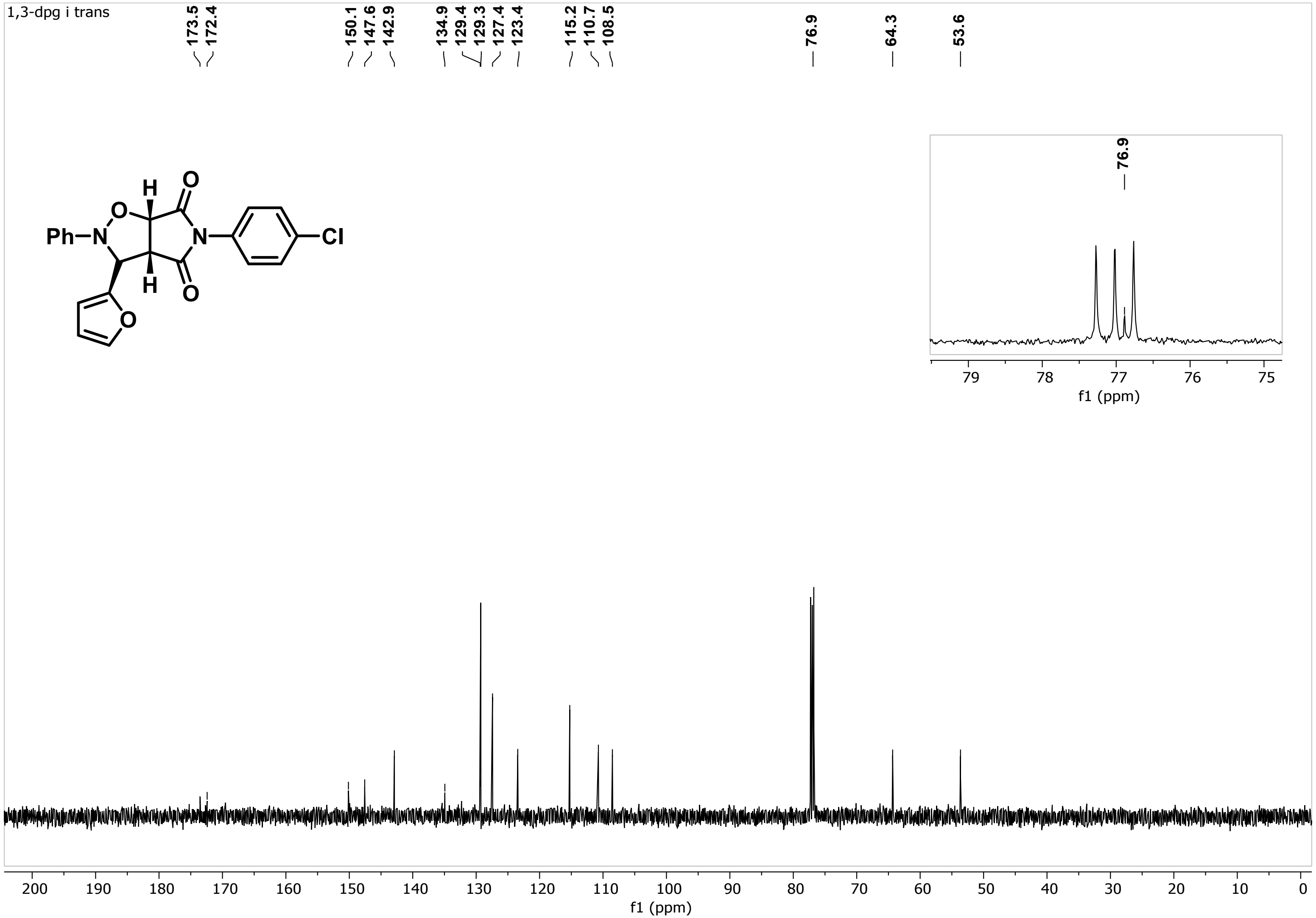


**Figure S45.** $^{13}$C NMR spectrum of **3j** ***(trans isomer)*** (125 MHz, $CDCl_3$).

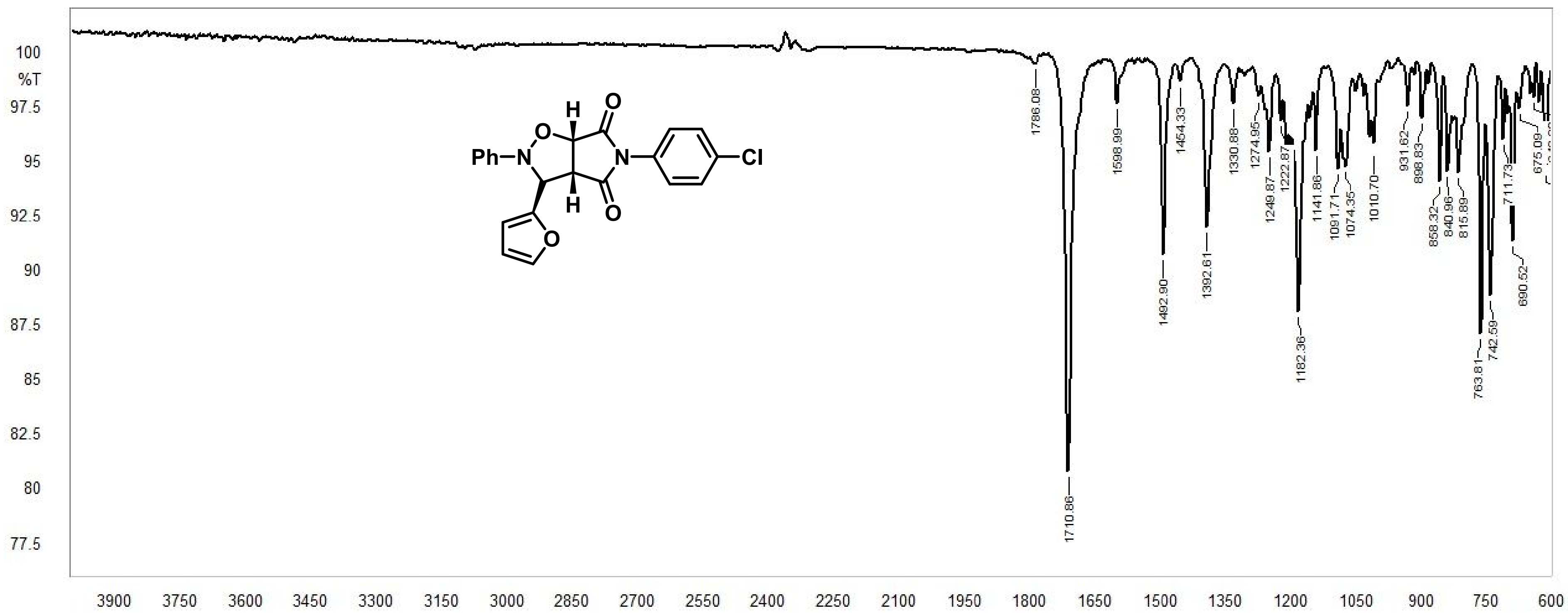


**Figure S46.** FT-IR spectrum of **3j** ***(trans isomer)***.

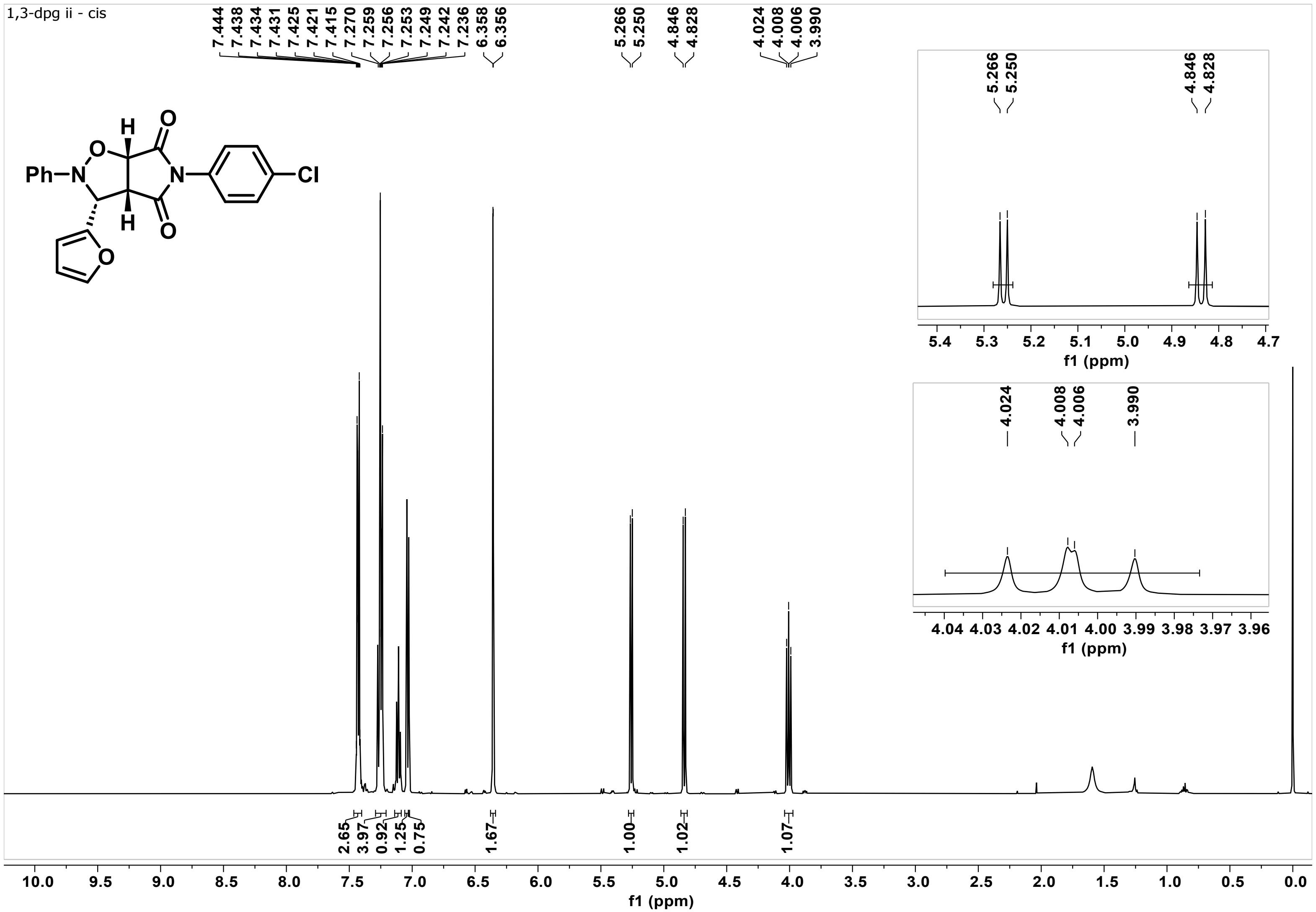


**Figure S47.** $^{1}$H NMR spectrum of **3j** ***(cis isomer)*** (500 MHz, $CDCl_3$).

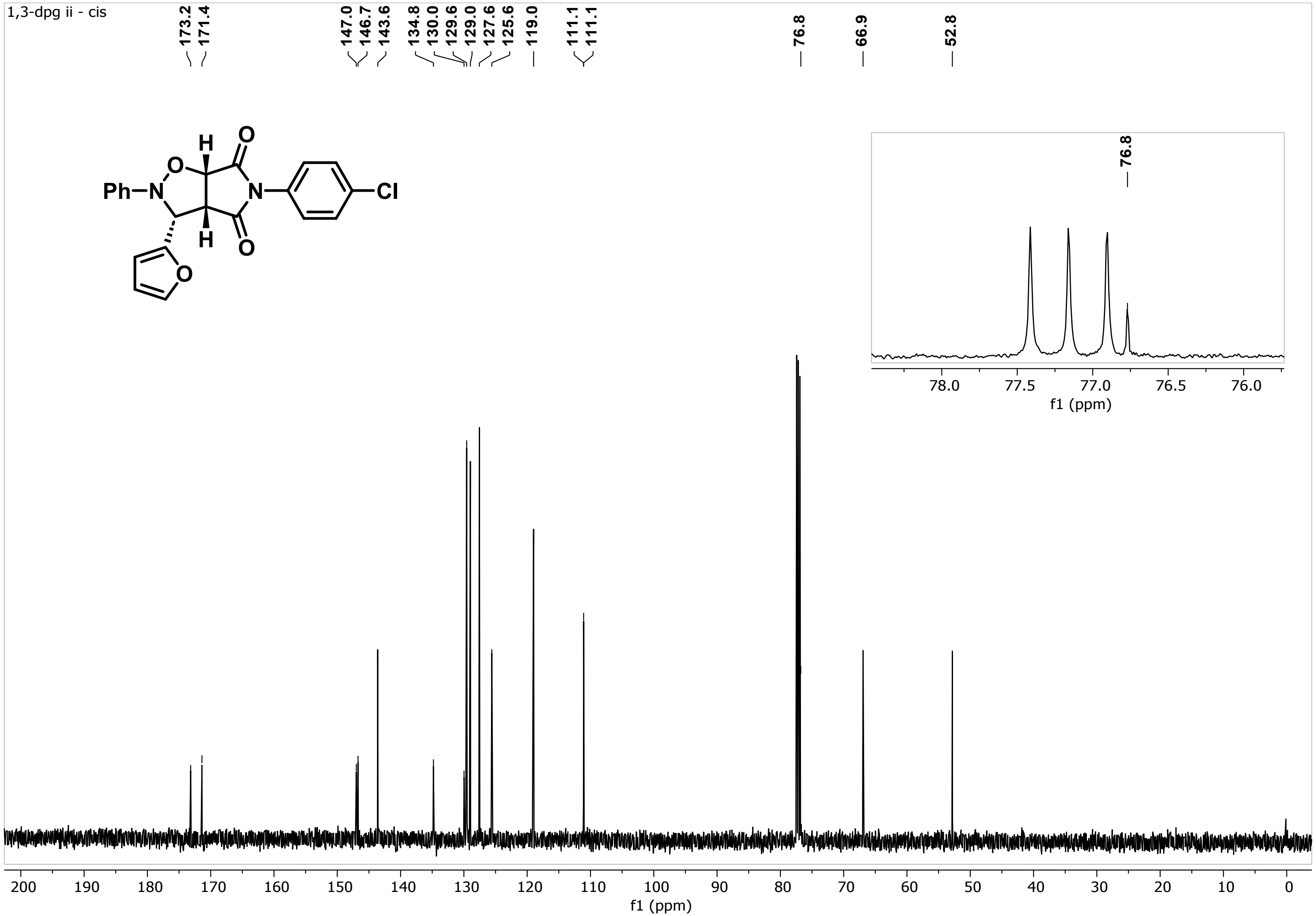


**Figure S48.** $^{13}$C NMR spectrum of **3j** ***(cis isomer)*** (125 MHz, $CDCl_3$).

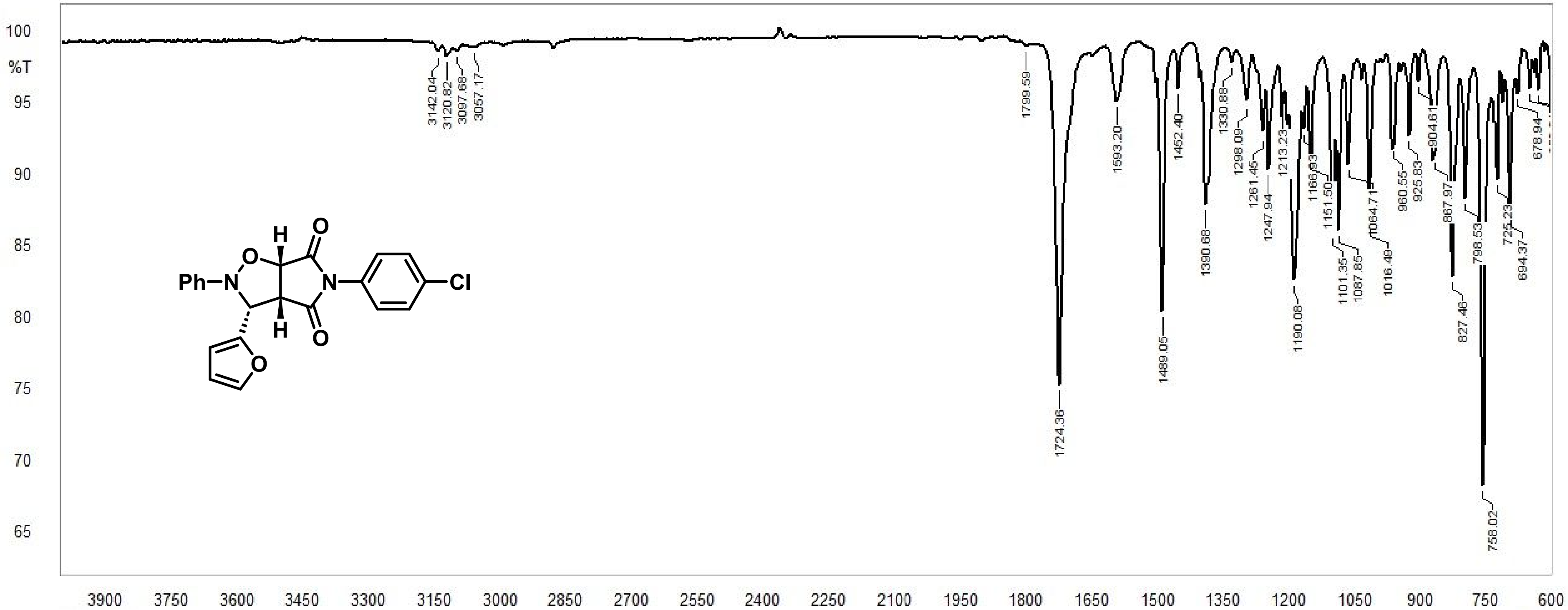

**Figure S49.** FT-IR spectrum of **3j** ***(cis isomer)***.

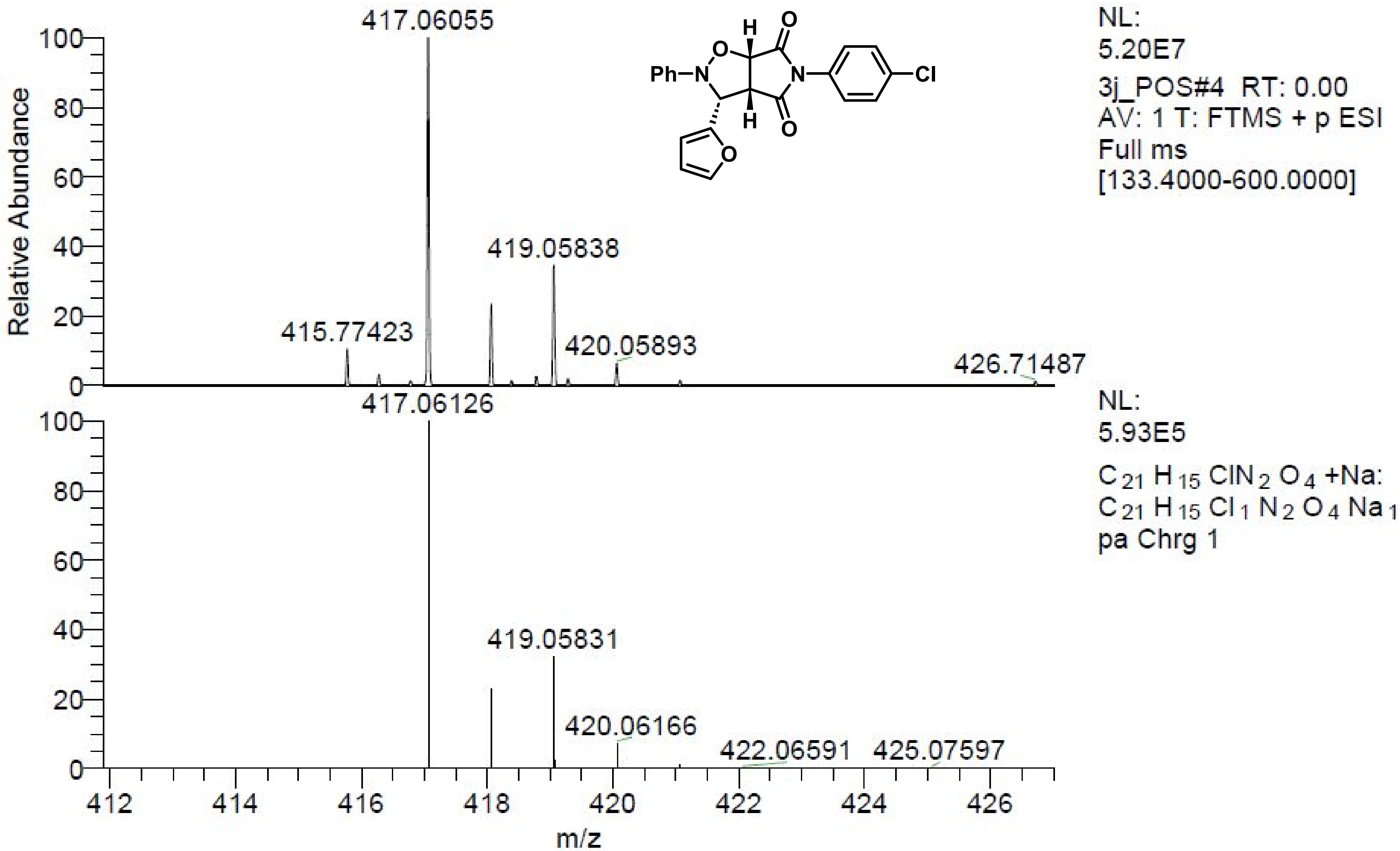


**Figure S50.** HRMS spectrum of **3j**.

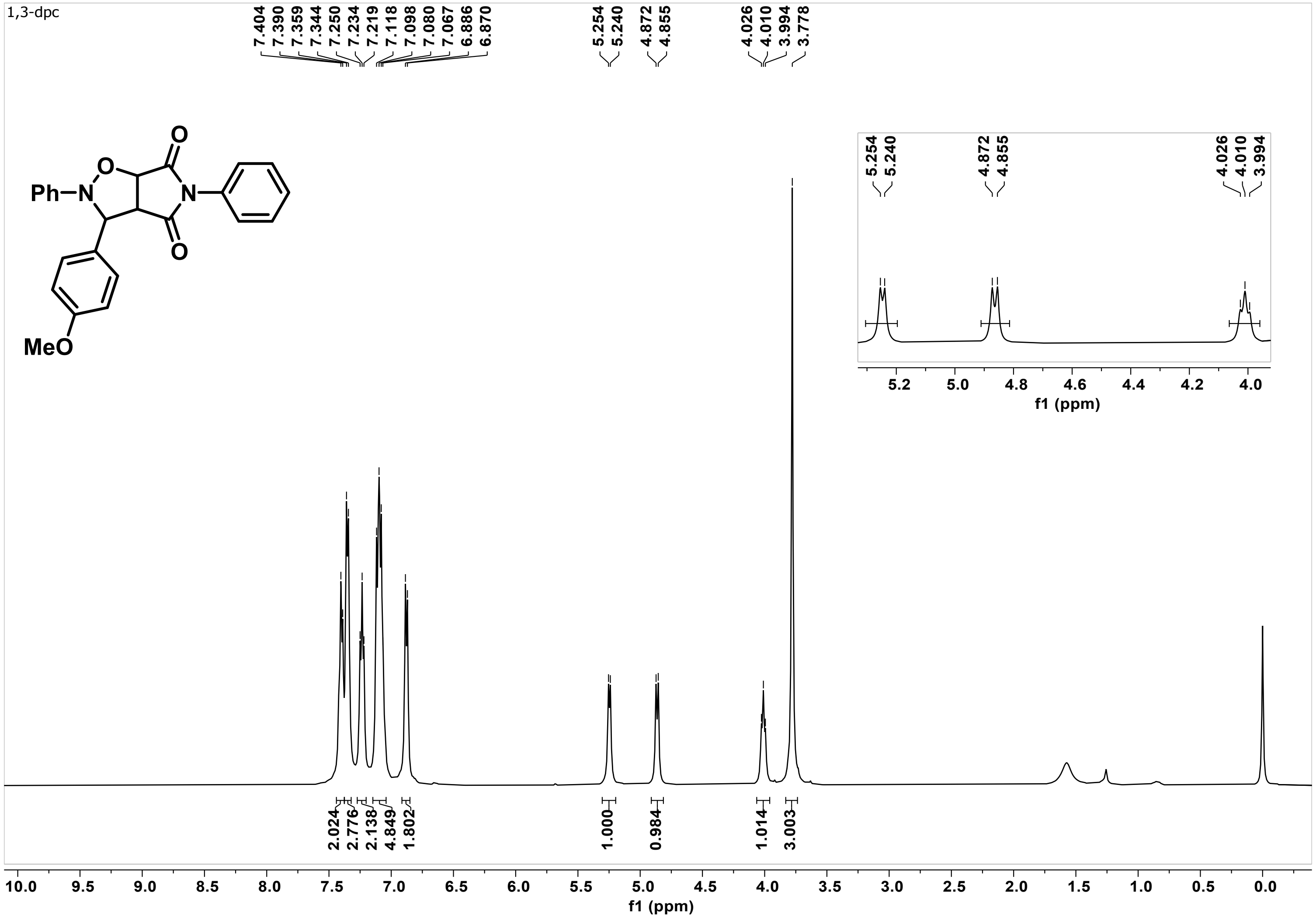


**Figure S51.** $^{1}$H NMR spectrum of **3k** ***(cis isomer)*** (500 MHz, $CDCl_3$).

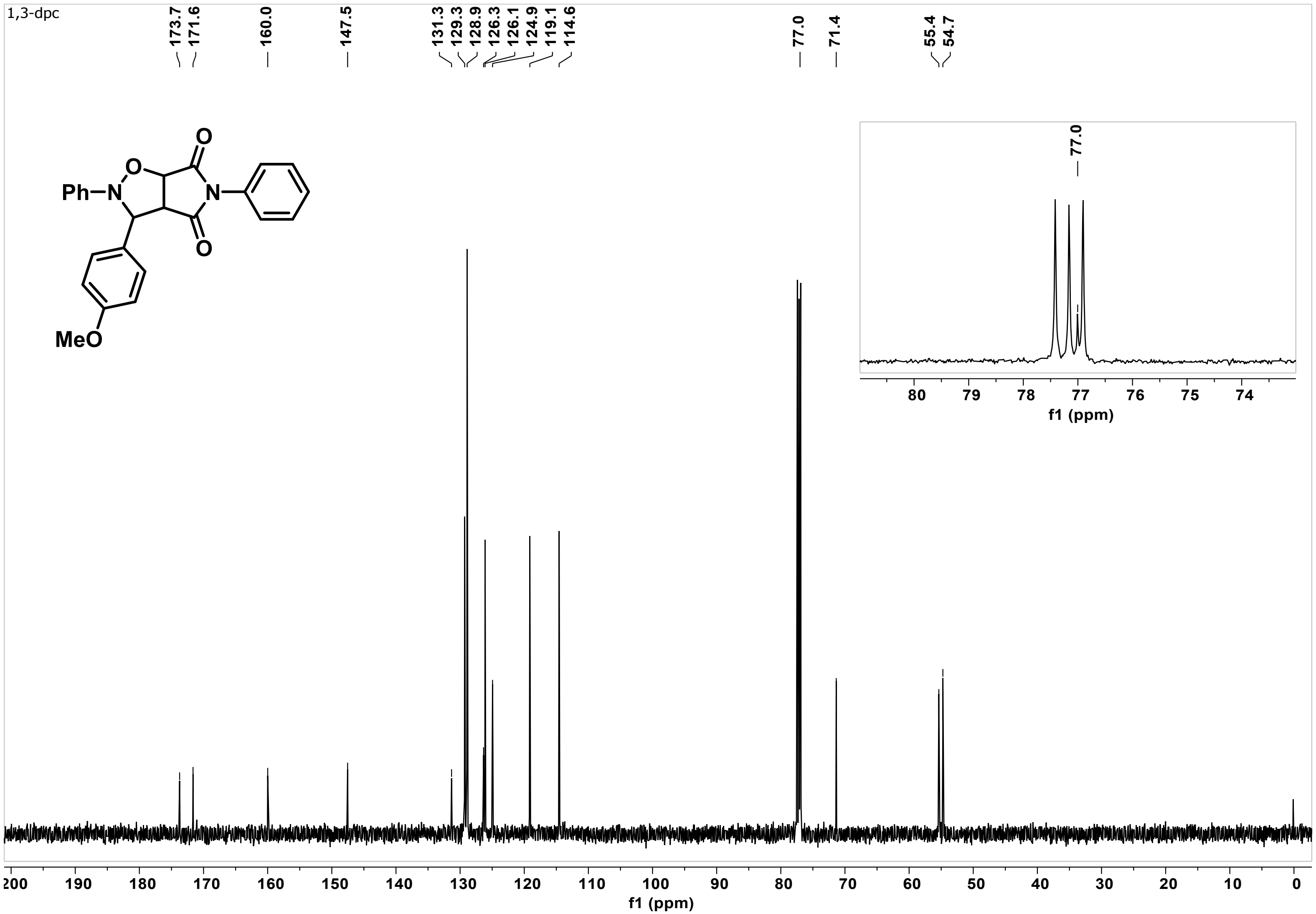


**Figure S52.** $^{13}$C NMR spectrum of **3k** ***(cis isomer)*** (125 MHz, $CDCl_3$).

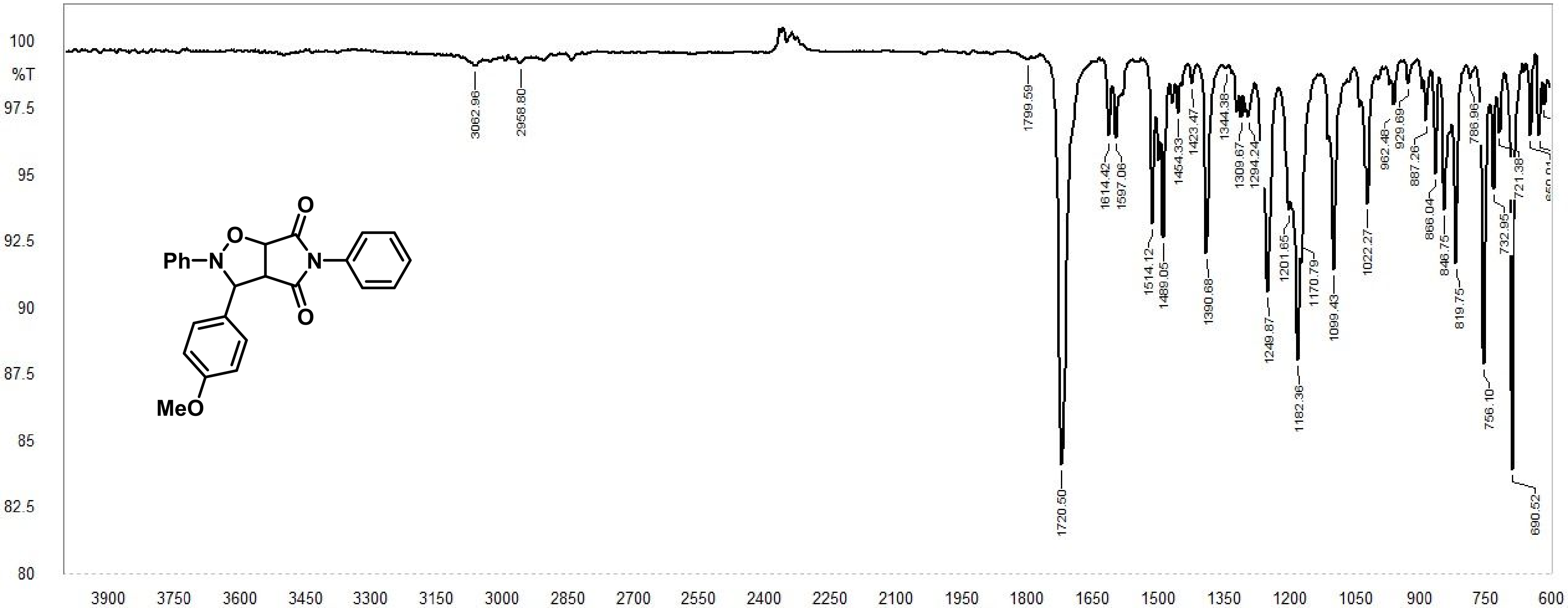

**Figure S53.** FT-IR spectrum of **3k** ***(cis isomer)***.

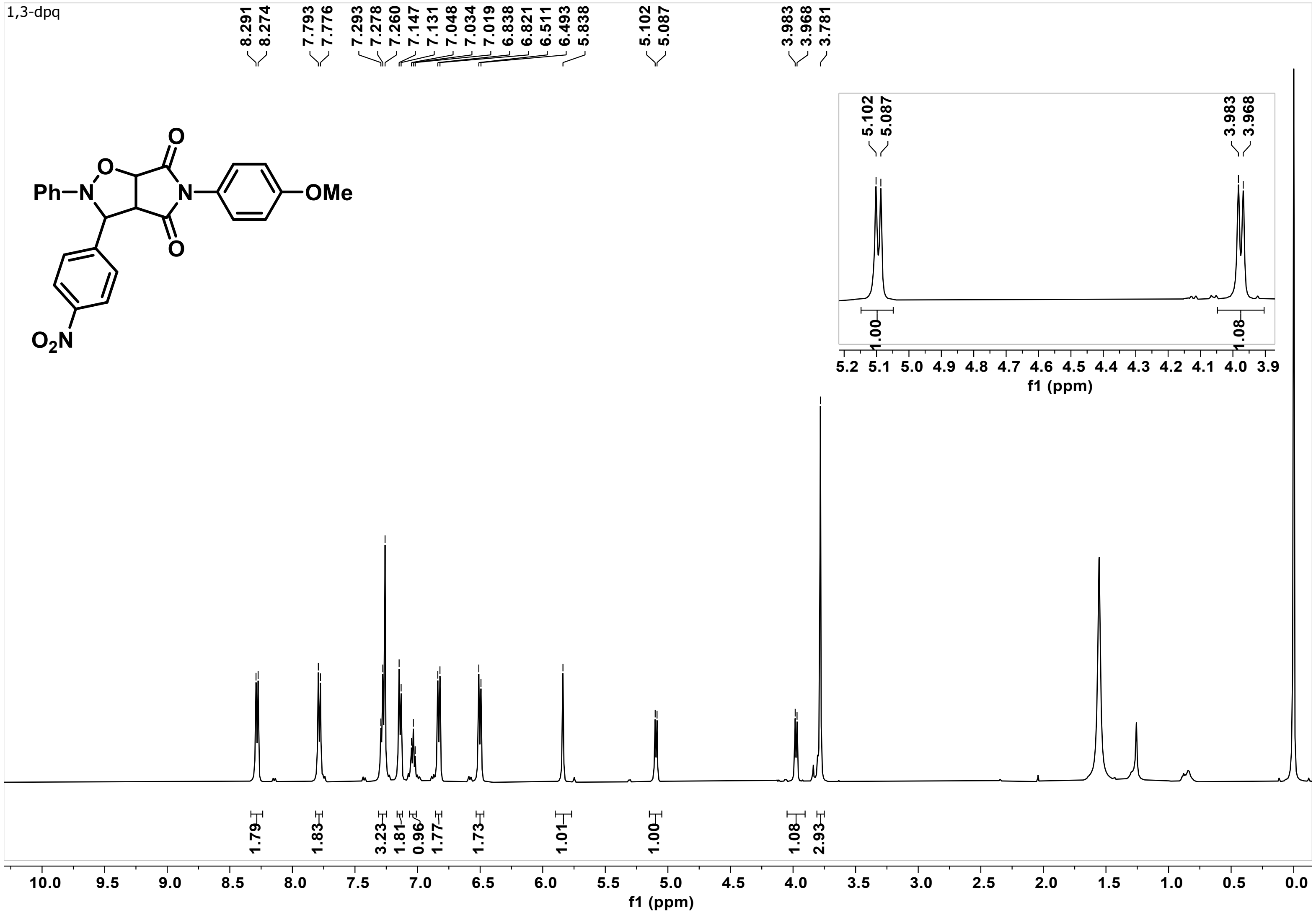


**Figure S54.** $^1$H NMR spectrum of **3l *(trans isomer)*** (500 MHz, $CDCl_3$).

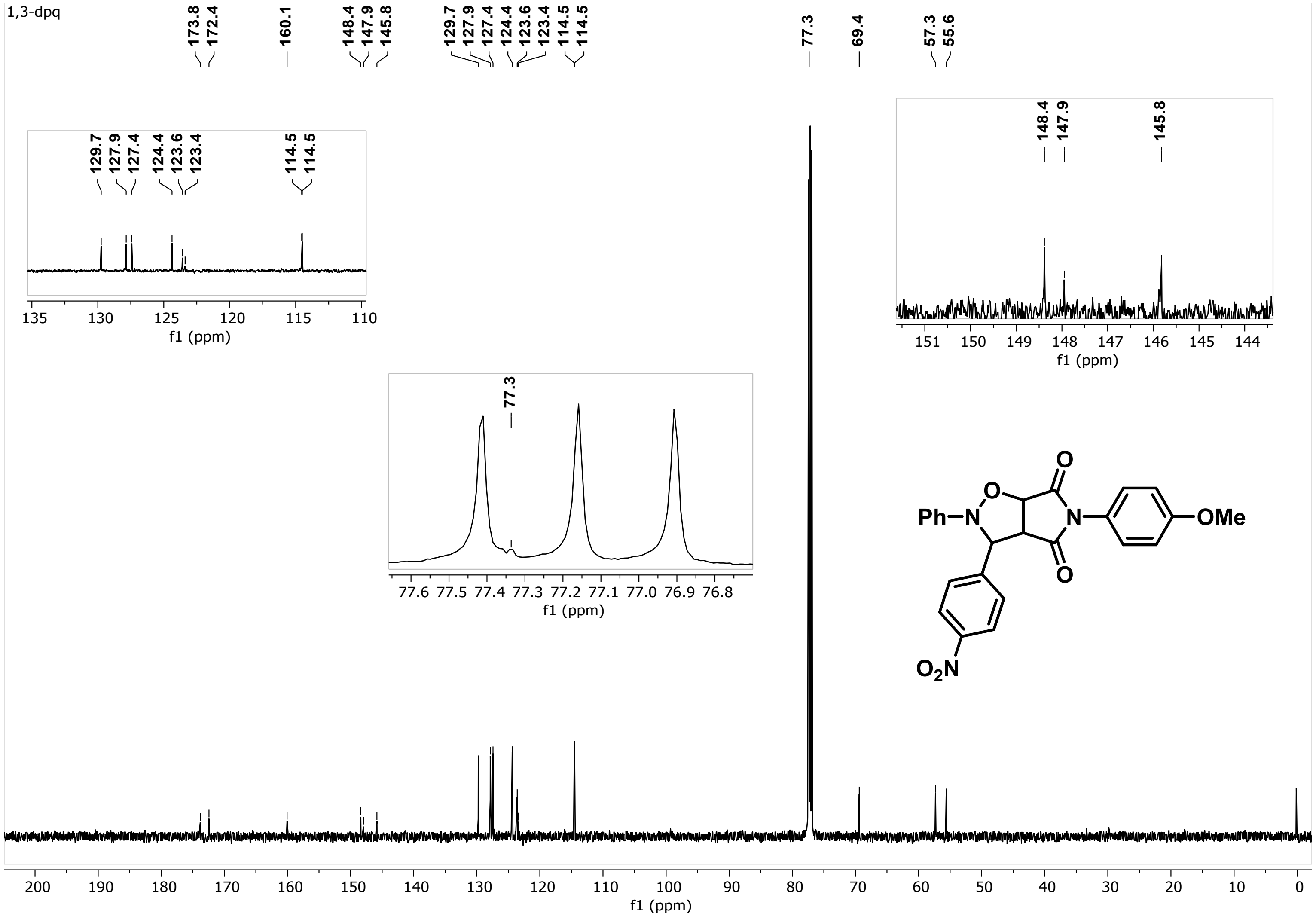


**Figure S55.** $^{13}$C NMR spectrum of **3l** ***(trans isomer)*** (125 MHz, $CDCl_3$).

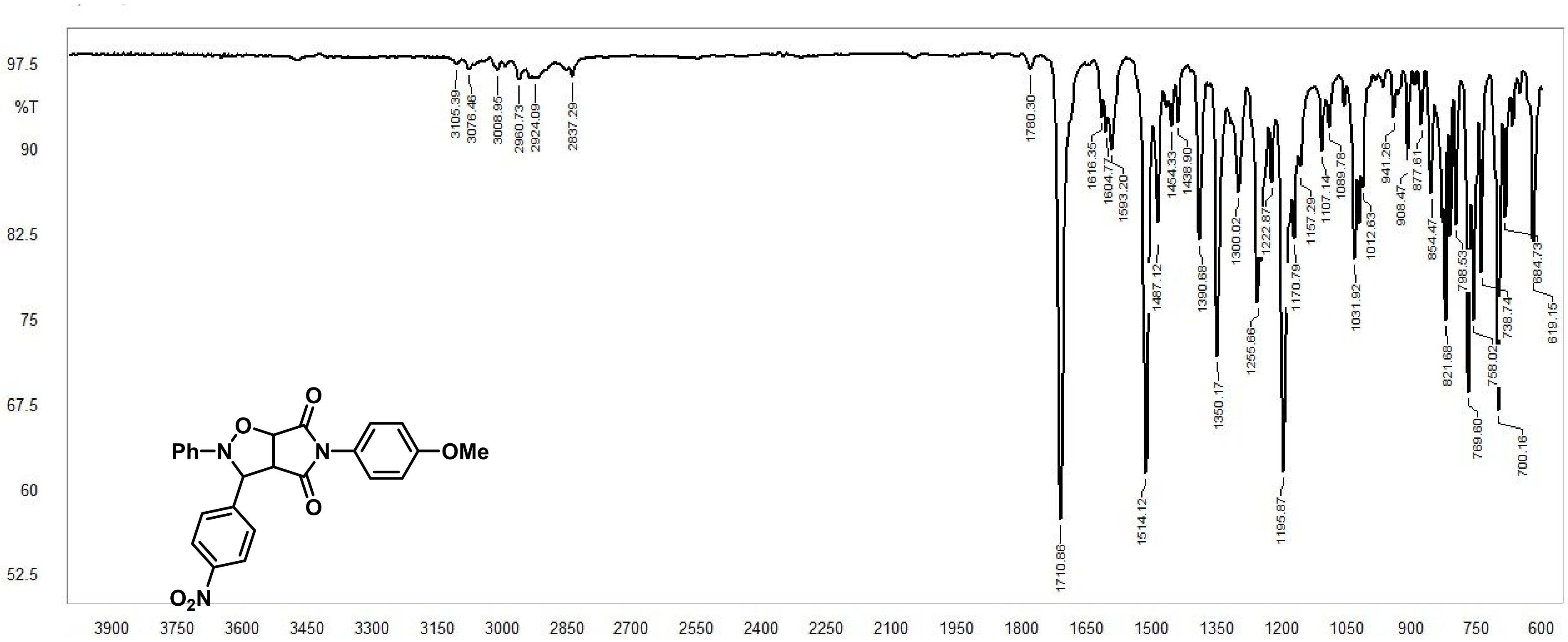


**Figure S56.** FT-IR spectrum of **3l** ***(trans isomer)*****.**

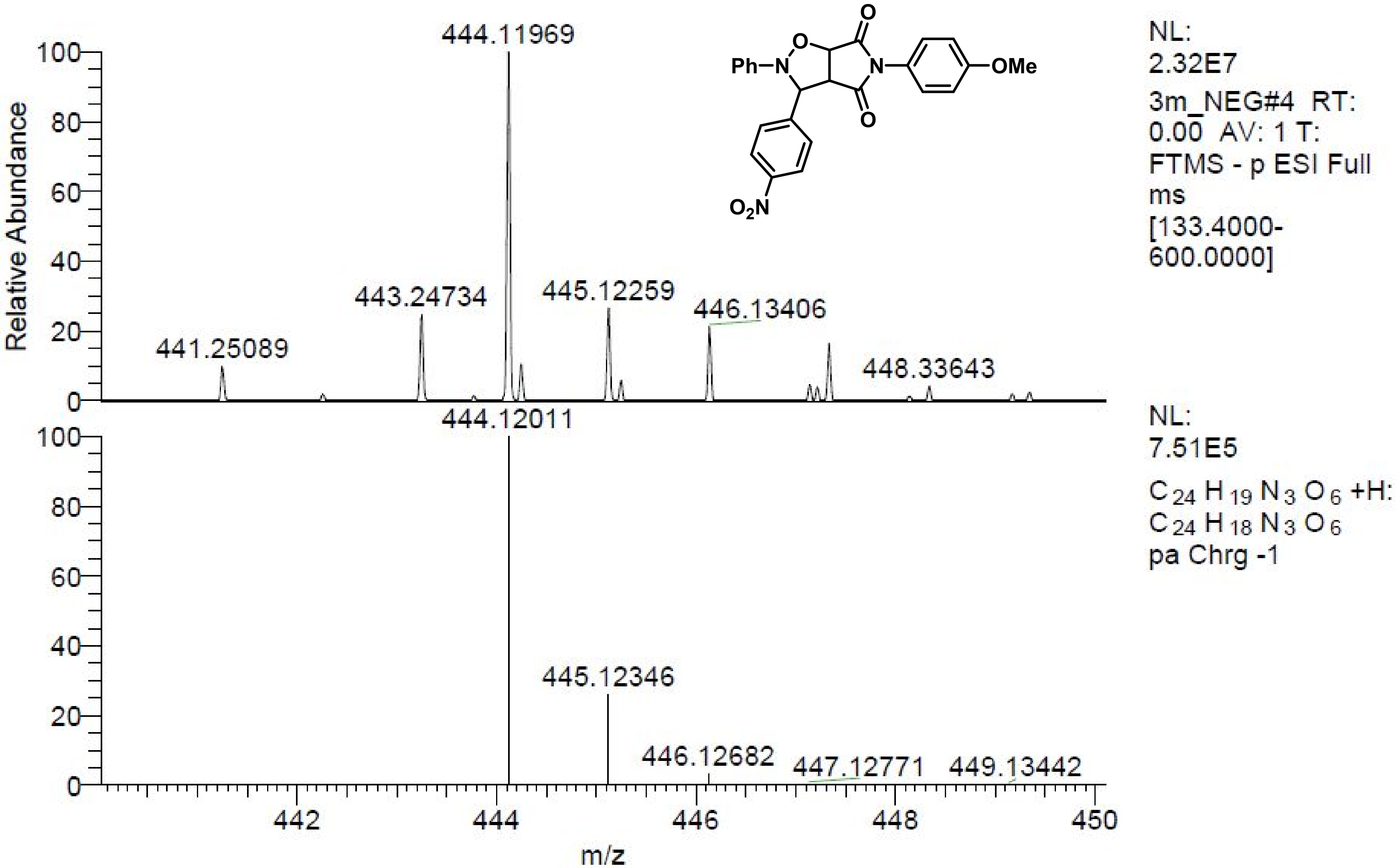


**Figure S57.** HRMS spectrum of **3l** ***(trans isomer)*.**

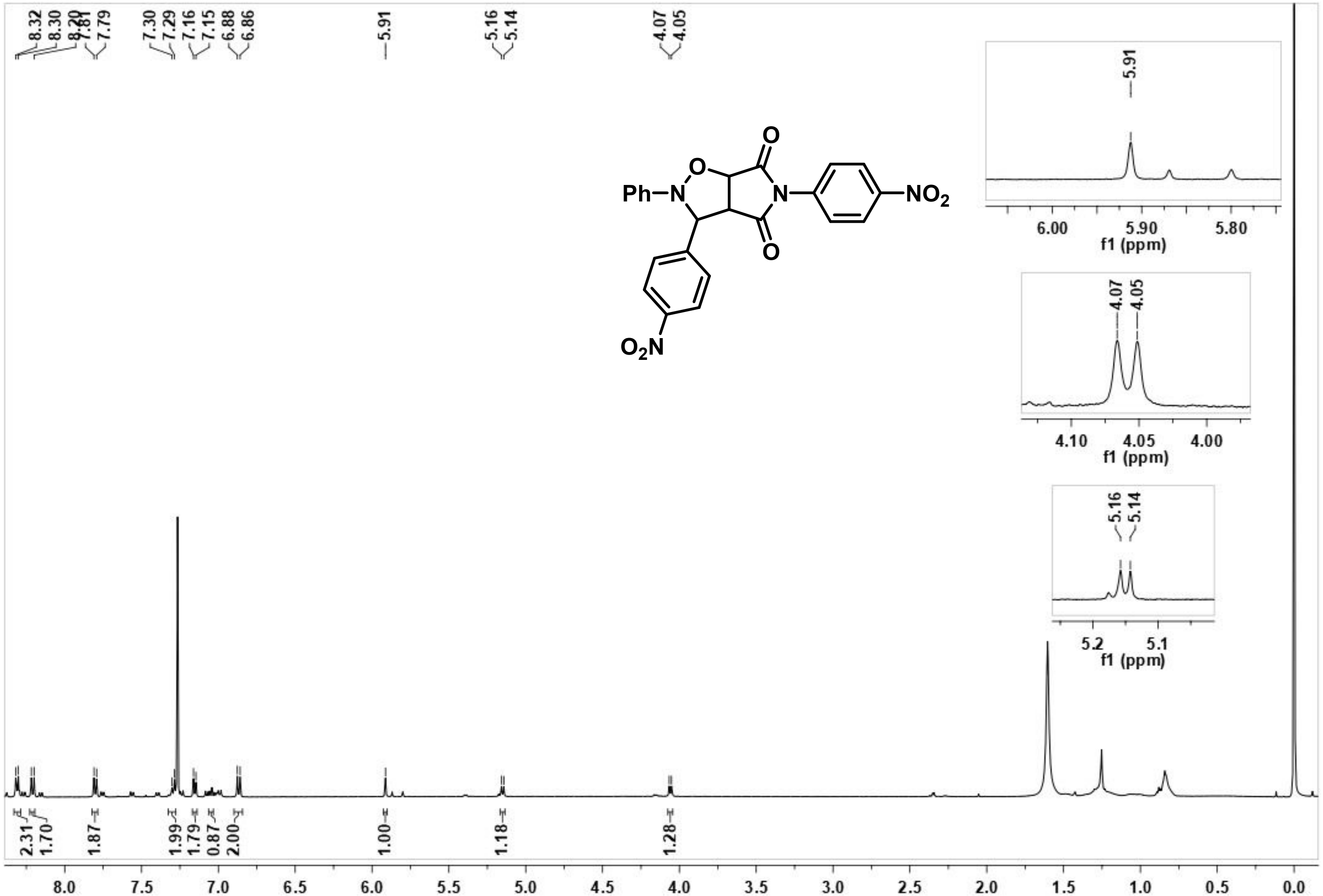

**Figure S58**. $^{1}$H NMR spectrum of **3m** ***(trans isomer)*** (500 MHz, $CDCl_3$).

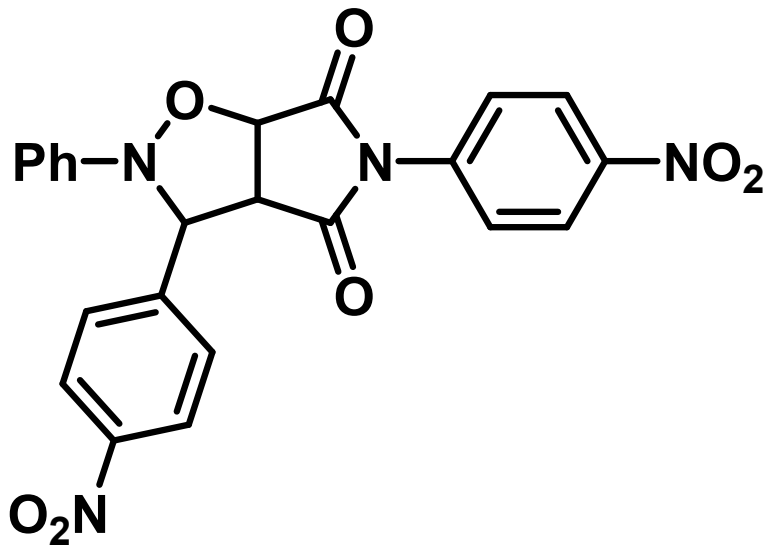


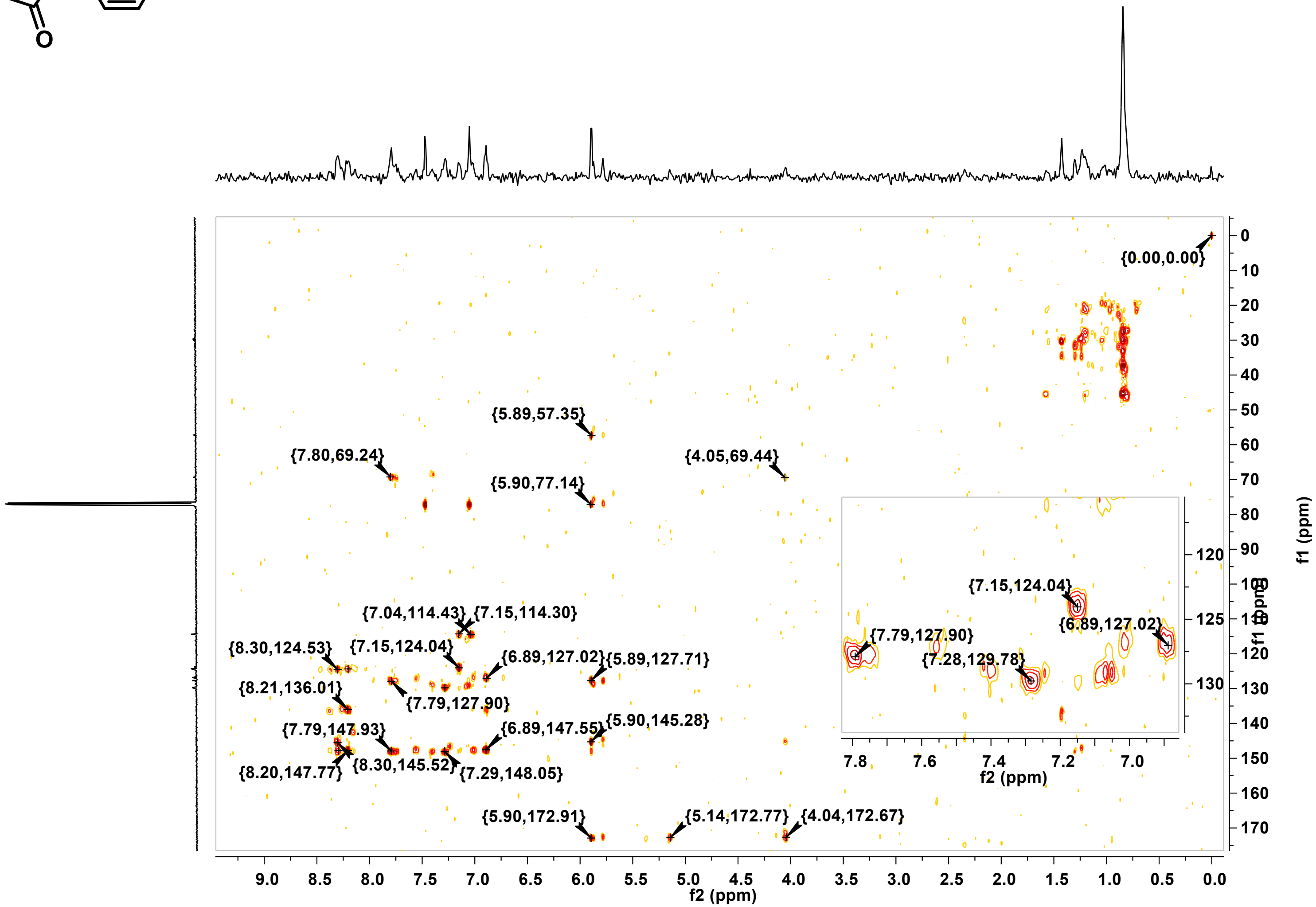


**Figure S59.** HMBC NMR spectrum of compound of **3m** ***(trans isomer)*** (125 MHz, $CDCl_3$).

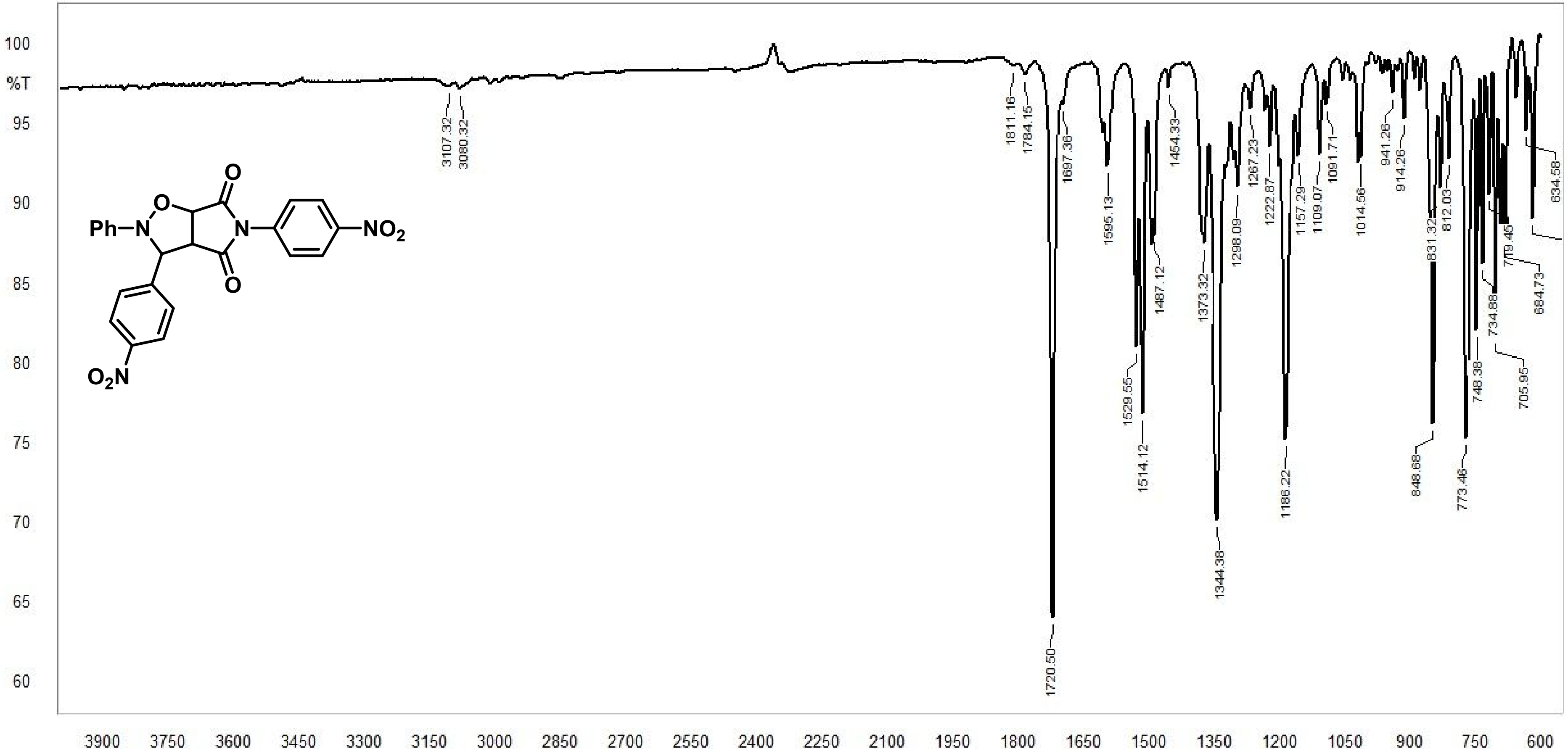


**Figure S60.** FT-IR spectrum of **3m** ***(trans isomer)***.

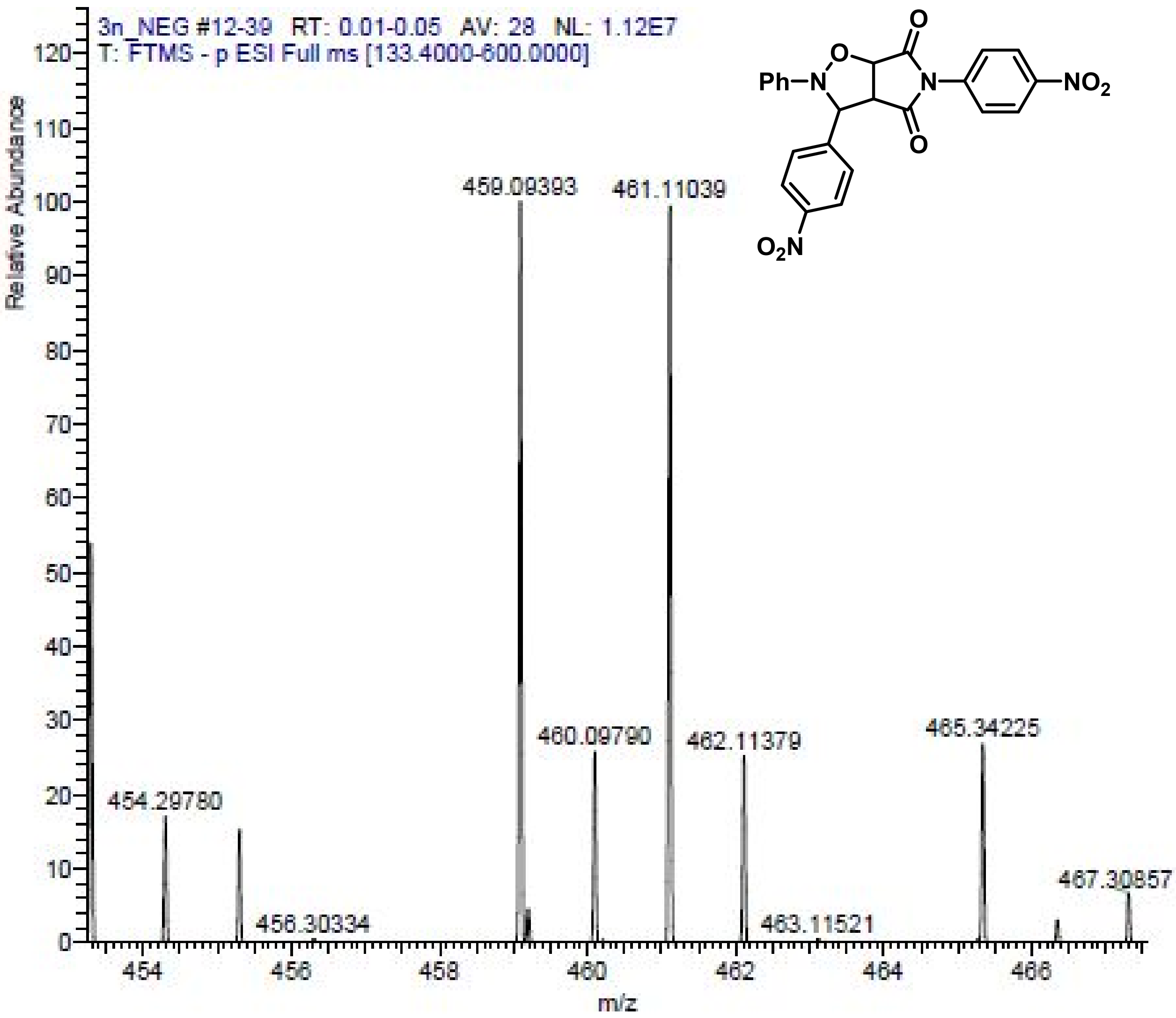


**Figure S61.** HRMS spectrum of **3m** ***(trans isomer)*****.**

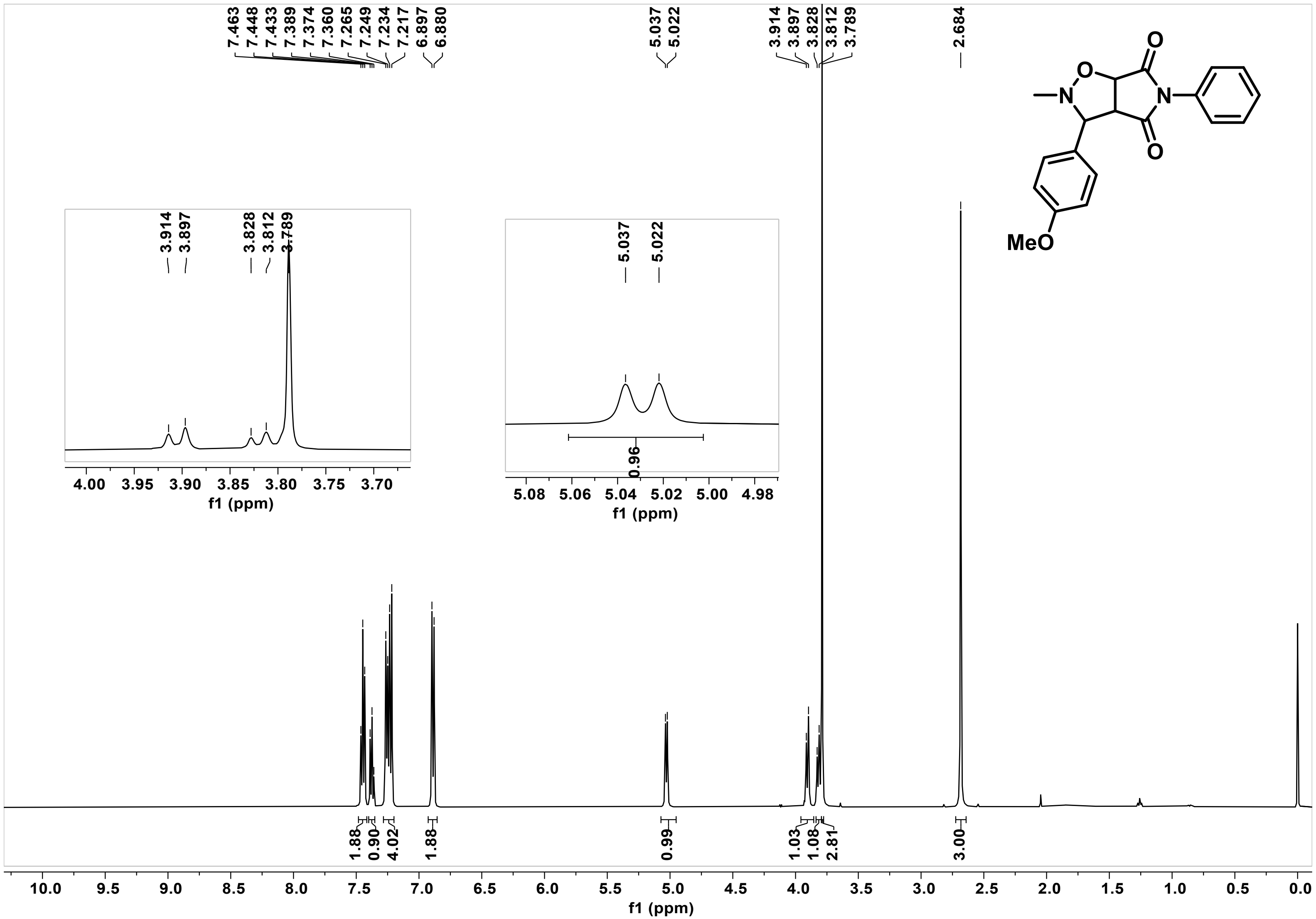


**Figure S62**. $^{1}$H NMR spectrum of **3m** ***(cis isomer)*** (500 MHz, $CDCl_3$).

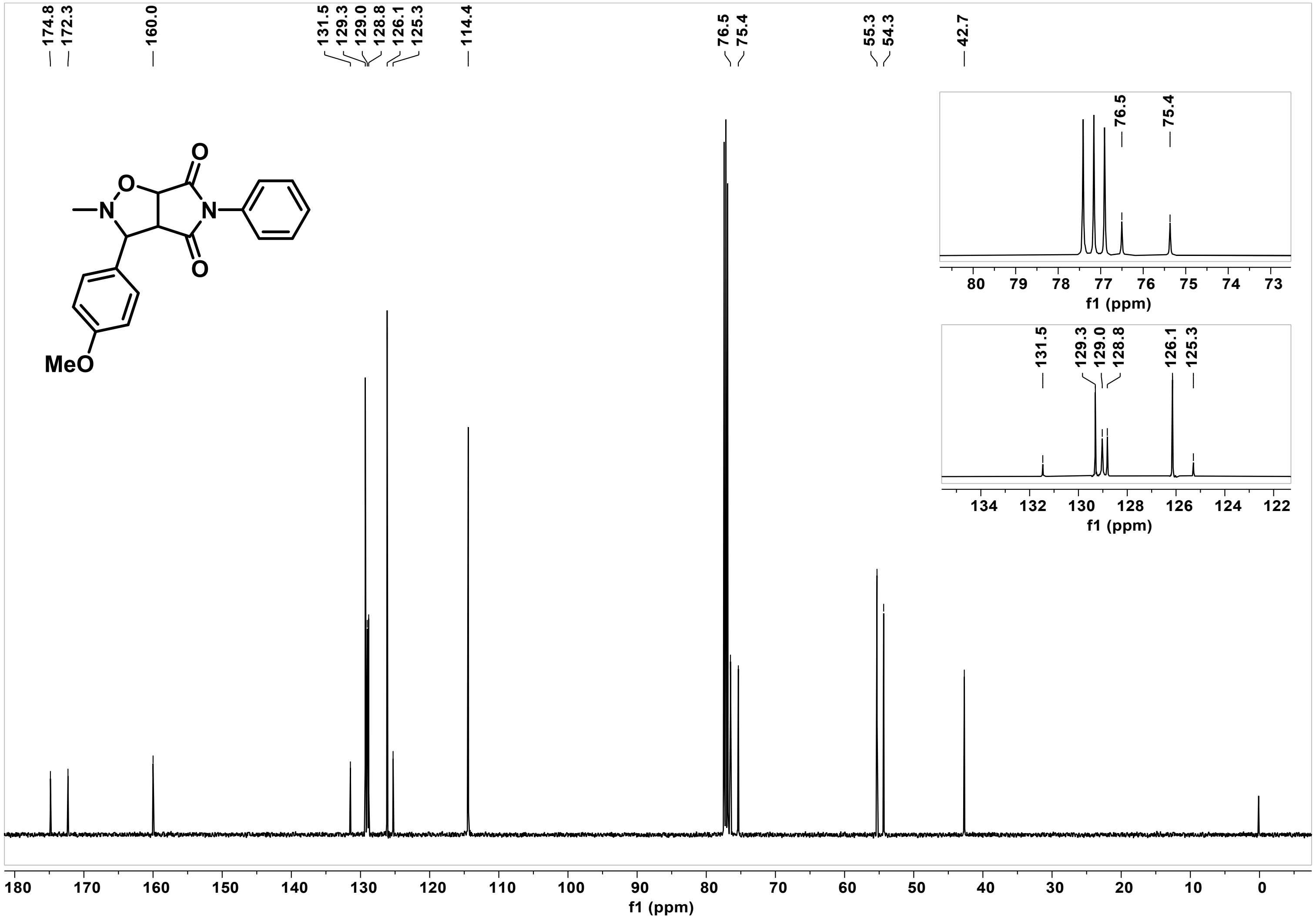


**Figure S63.** $^{13}C$ NMR spectrum of **3m** ***(cis isomer)*** (125 MHz, $CDCl_3$).

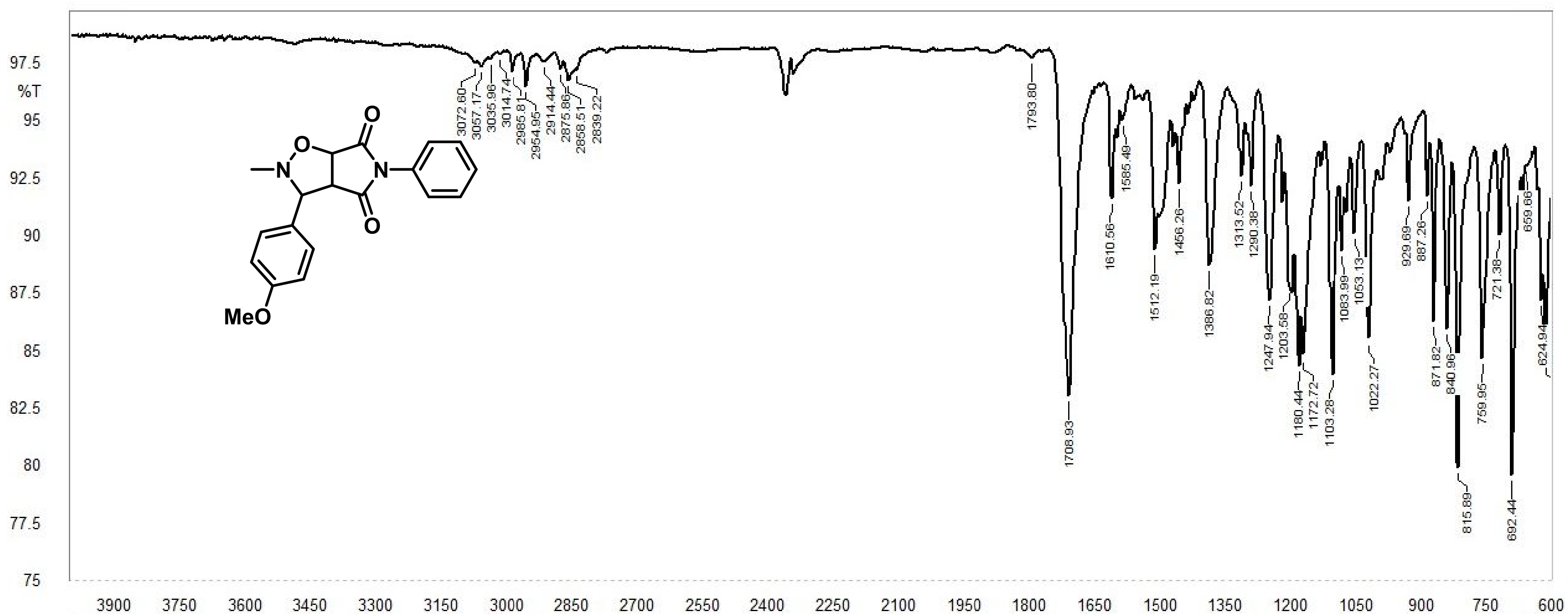


**Figure S64.** FT-IR spectrum of **3m** ***(cis isomer)*****.**

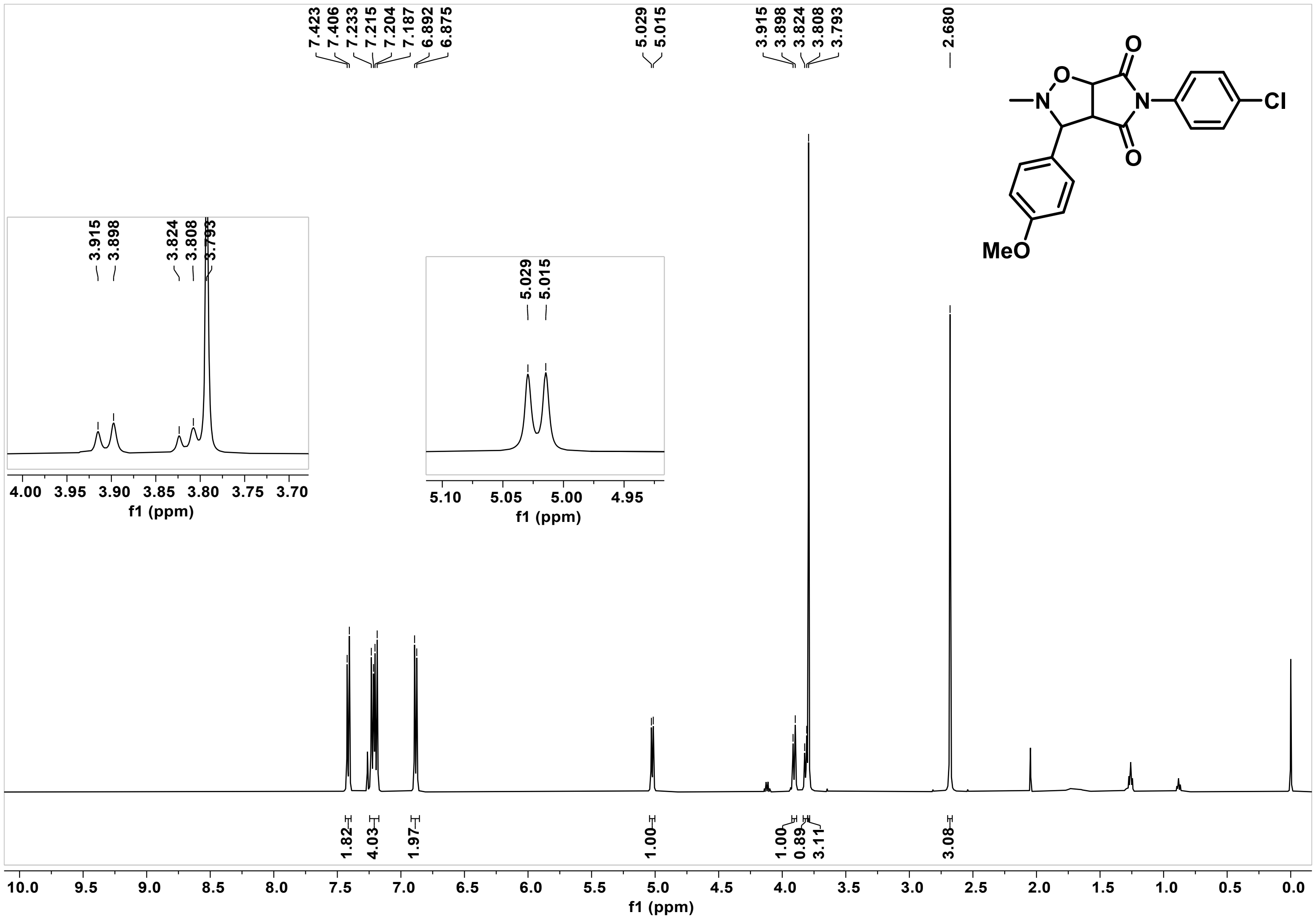

**Figure S65**. $^{1}$H NMR spectrum of **3o** ***(cis isomer)*** (500 MHz, $CDCl_3$).

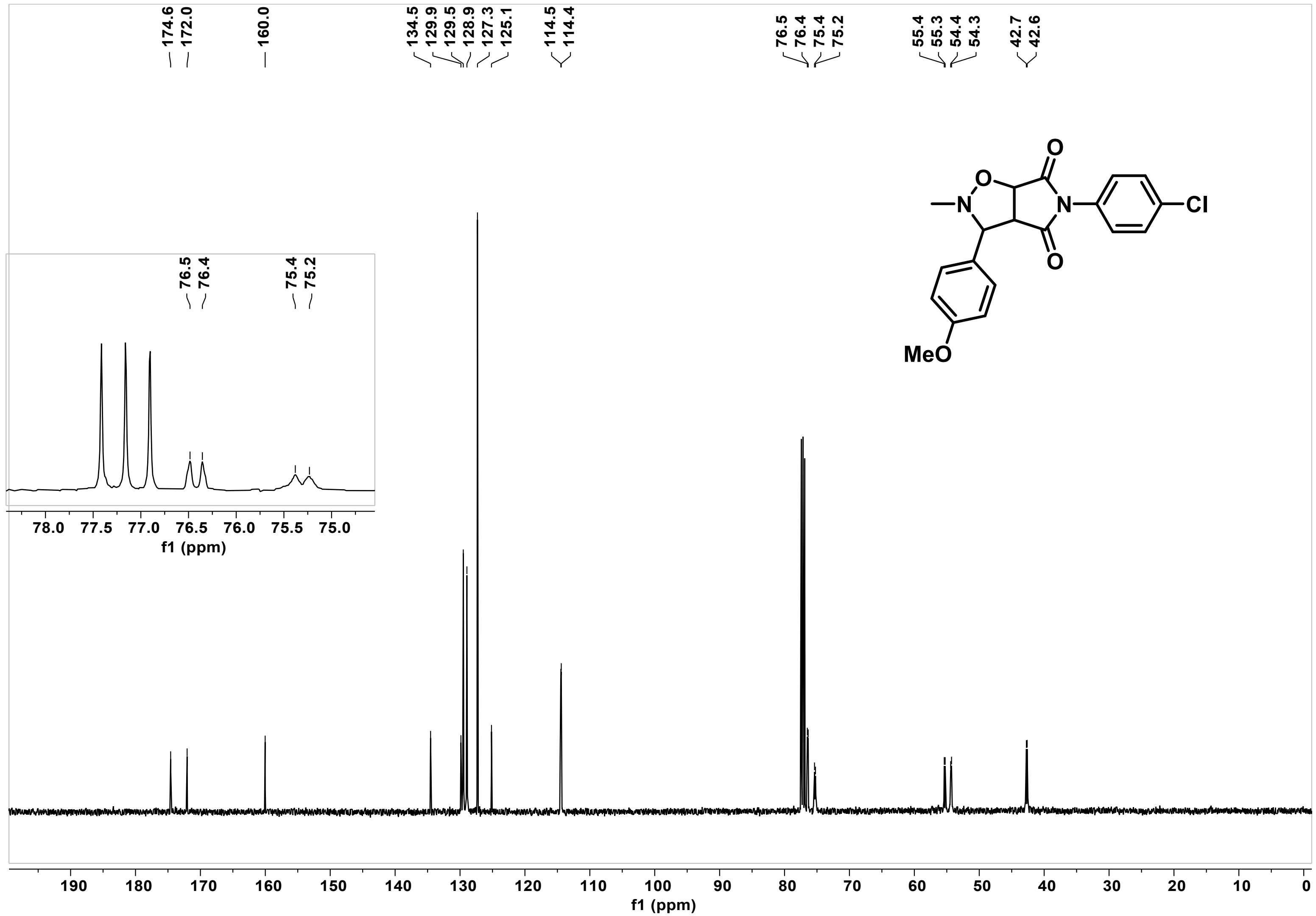


**Figure S66.** $^{13}$C NMR spectrum of **3o** ***(cis isomer)*** (125 MHz, $CDCl_3$).

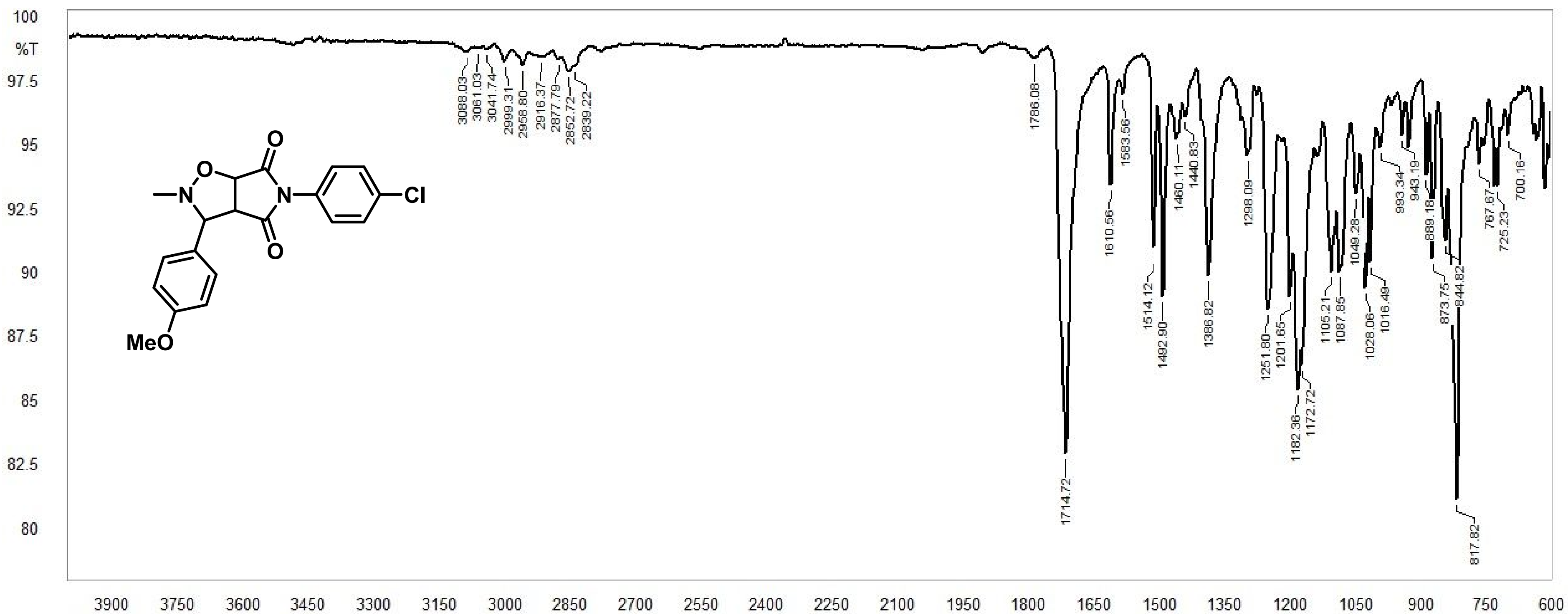

**Figure S67.** FT-IR spectrum of **3o** ***(cis isomer)*****.**

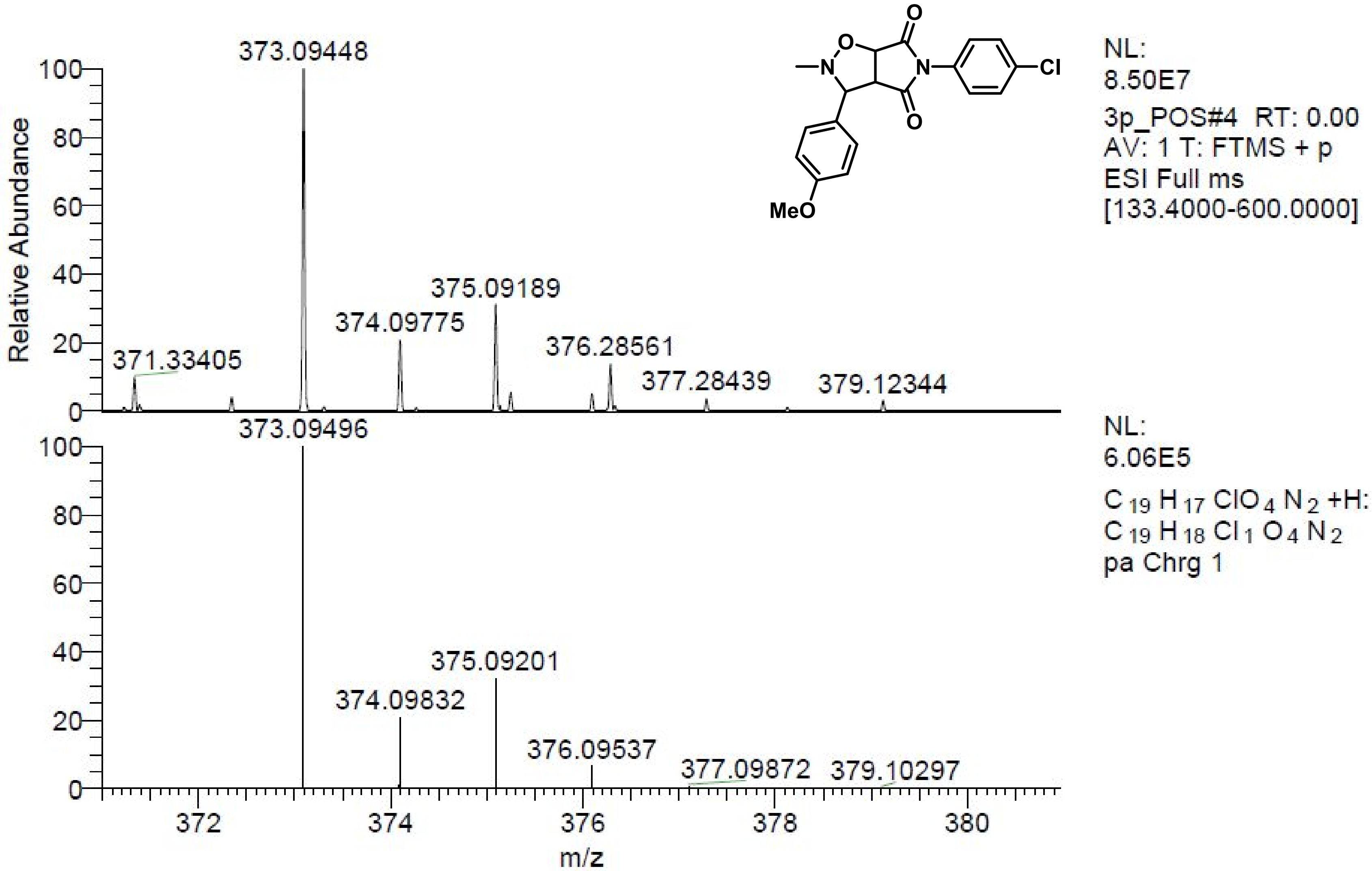


**Figure S68.** HRMS spectrum of **3o** ***(cis isomer)*.**

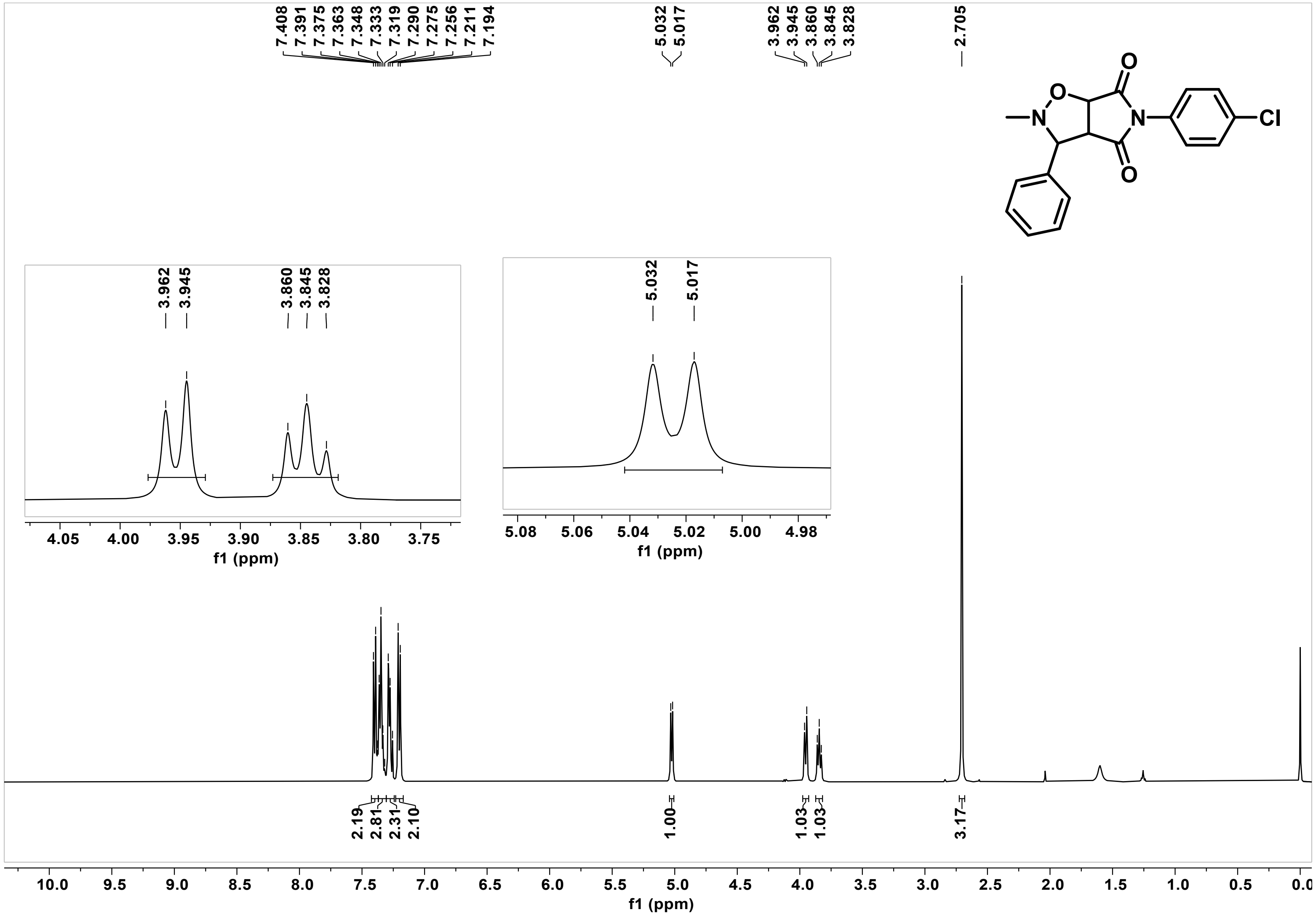


**Figure S69**. $^{1}$H NMR spectrum of **3p** ***(cis isomer)*** (500 MHz, $CDCl_3$).

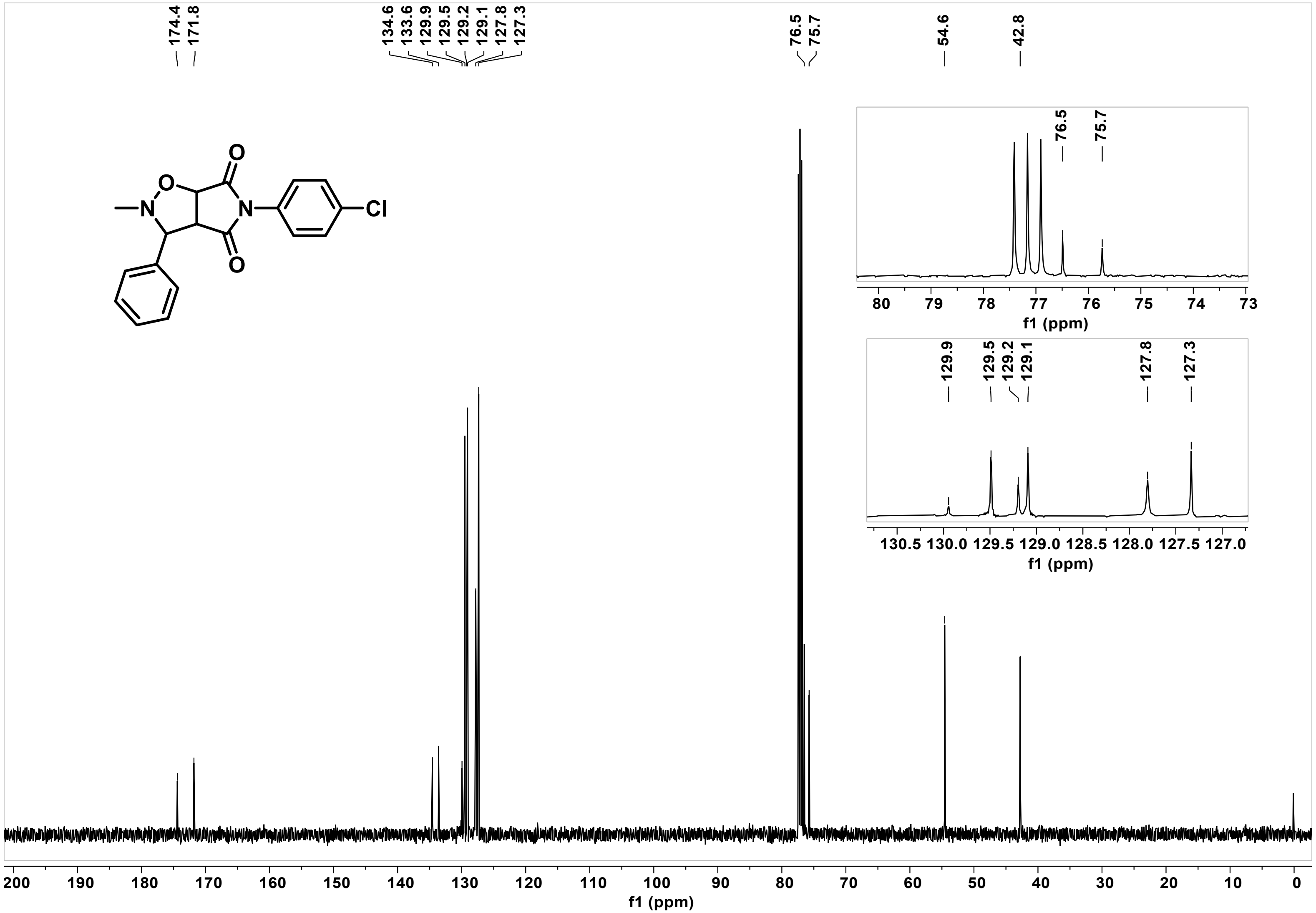


**Figure S70.** $^{13}$C NMR spectrum of **3p** ***(cis isomer)*** (125 MHz, $CDCl_3$).

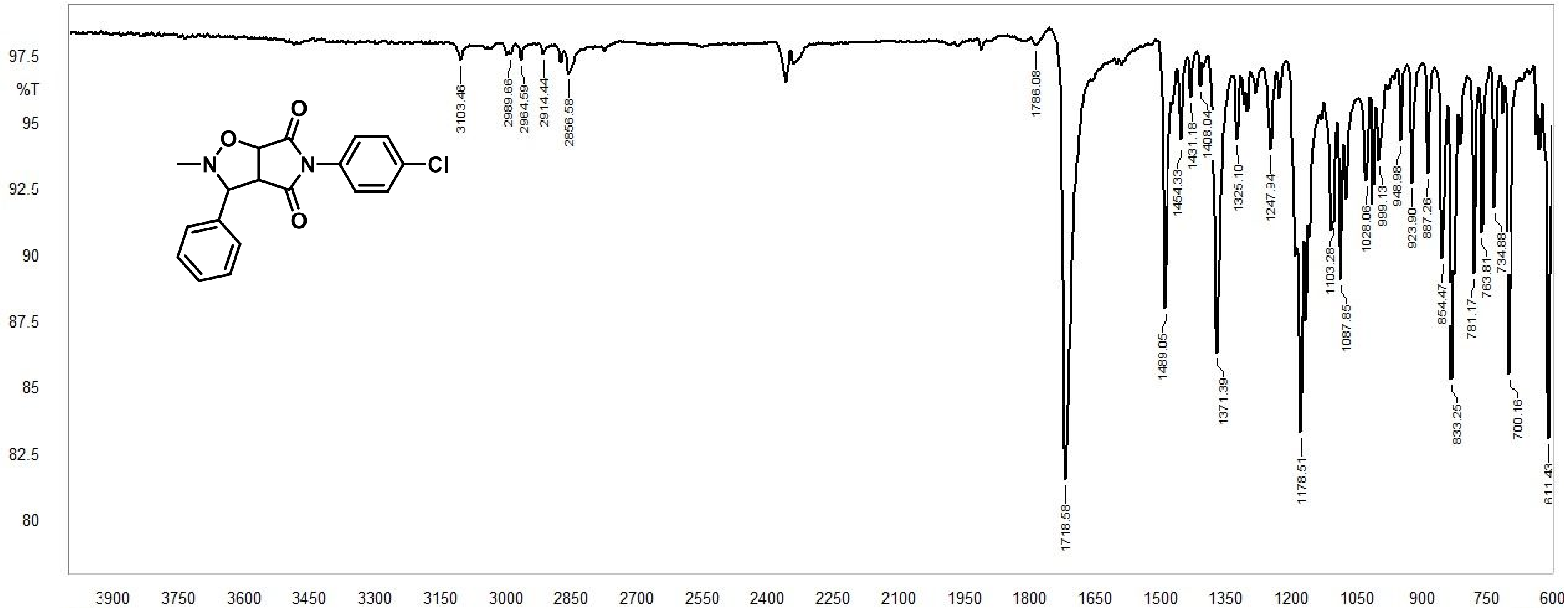


**Figure S71.** FT-IR spectrum of **3p** ***(cis isomer)*.**

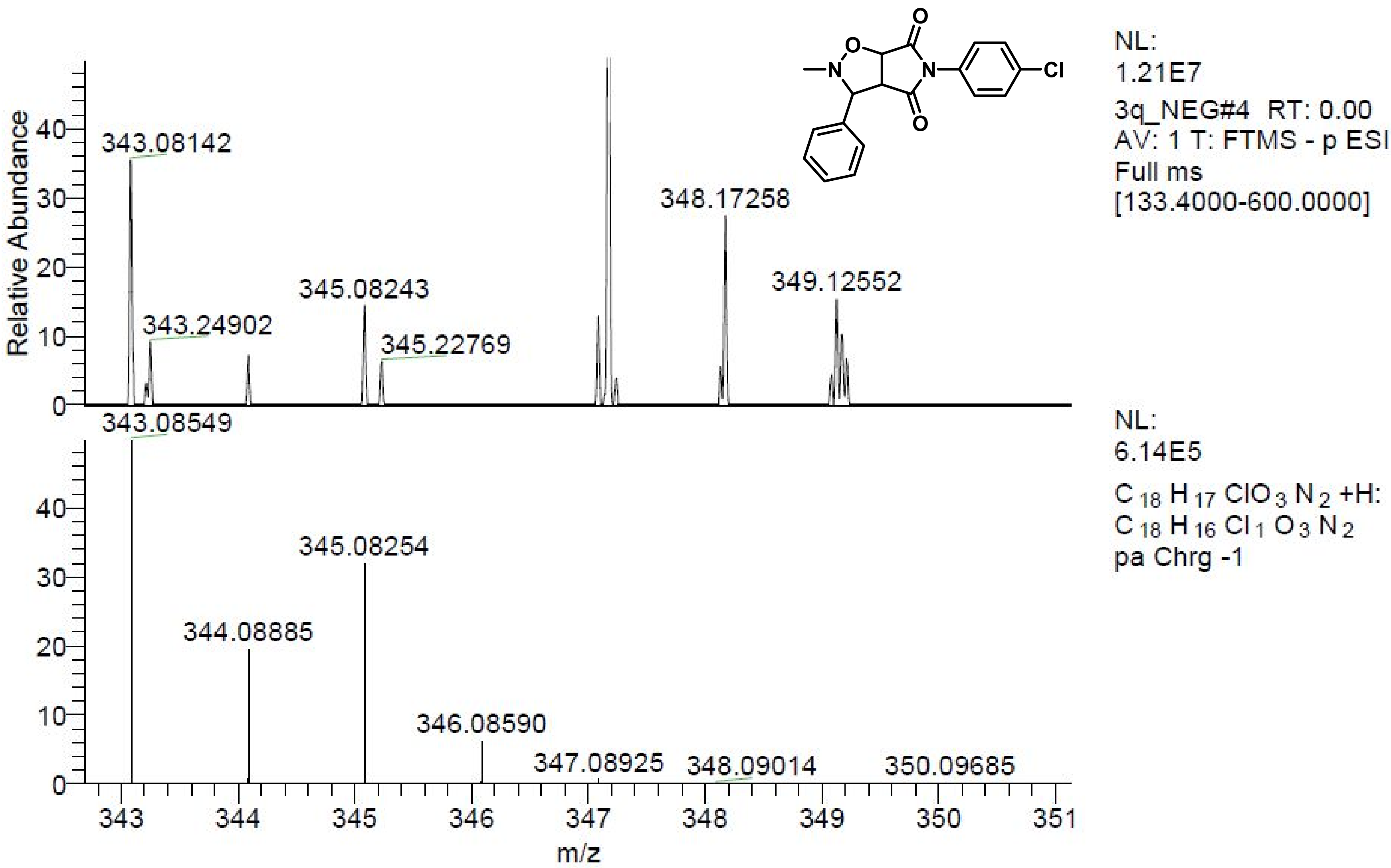


**Figure S72.** HRMS spectrum of **3p** ***(cis isomer)*****.**

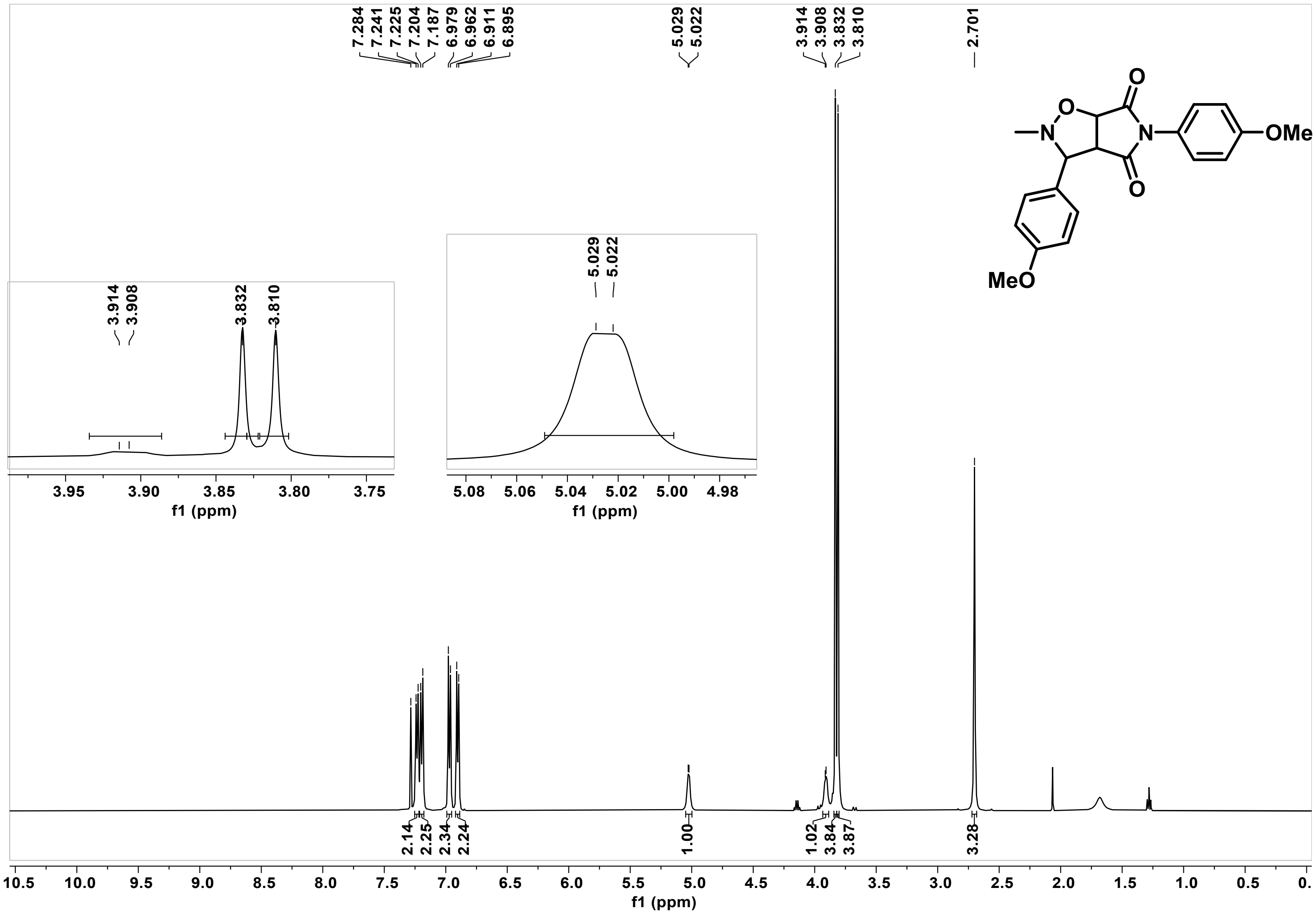


**Figure S73**. $^{1}$H NMR spectrum of **3q** ***(cis isomer)*** (500 MHz, $CDCl_3$).

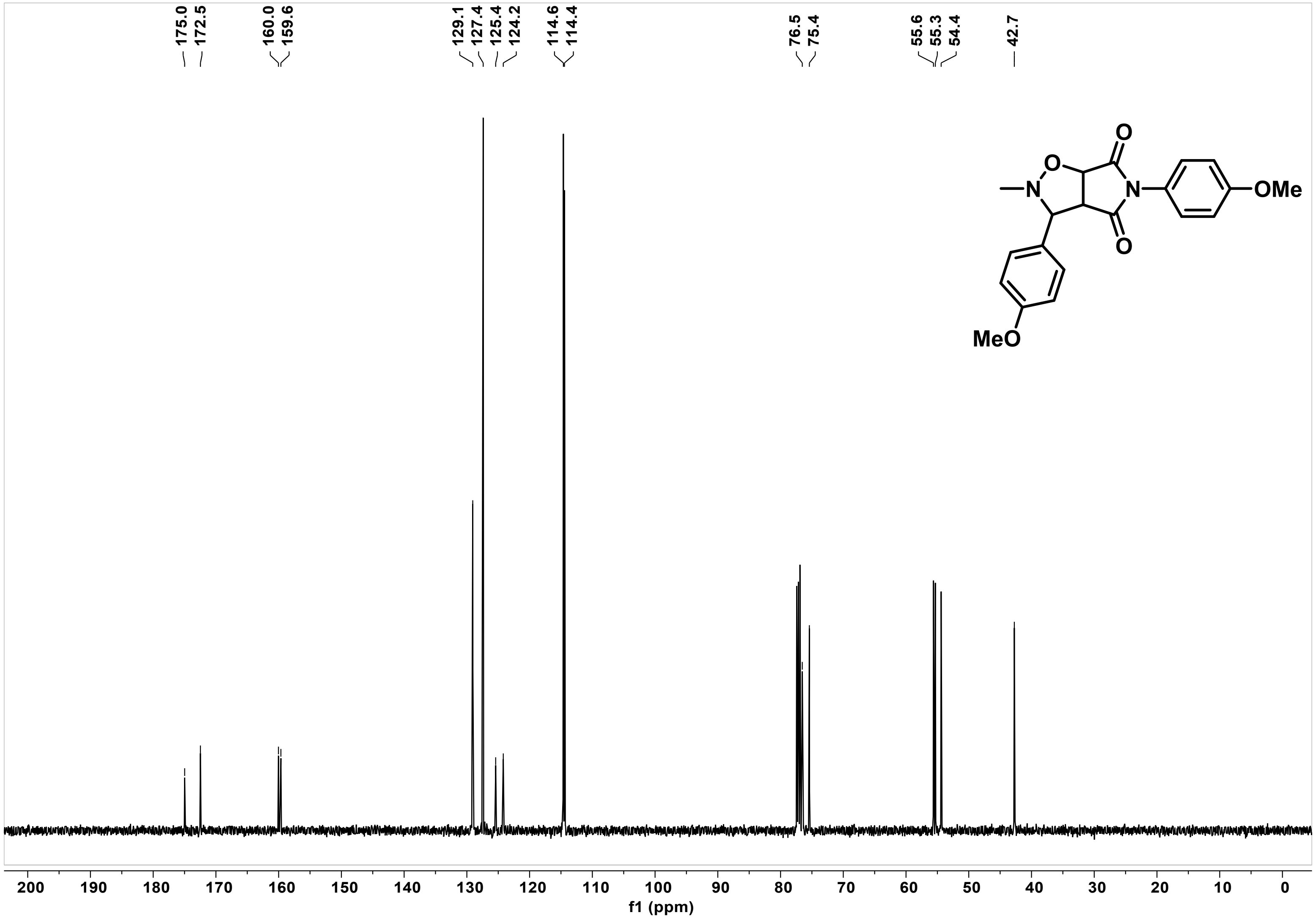


**Figure S74.** $^{13}C$ NMR spectrum of **3q** ***(cis isomer)*** (125 MHz, $CDCl_3$).

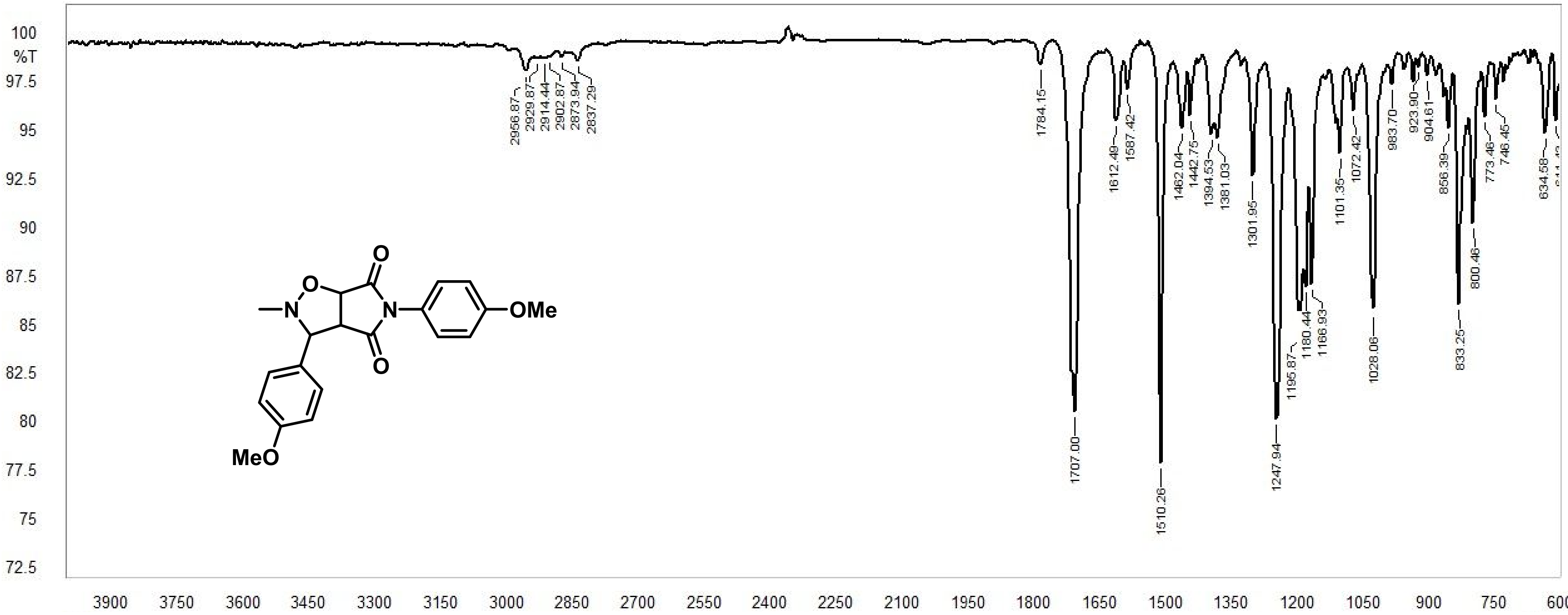


**Figure S75.** FT-IR spectrum of **3q** ***(cis isomer)*****.**

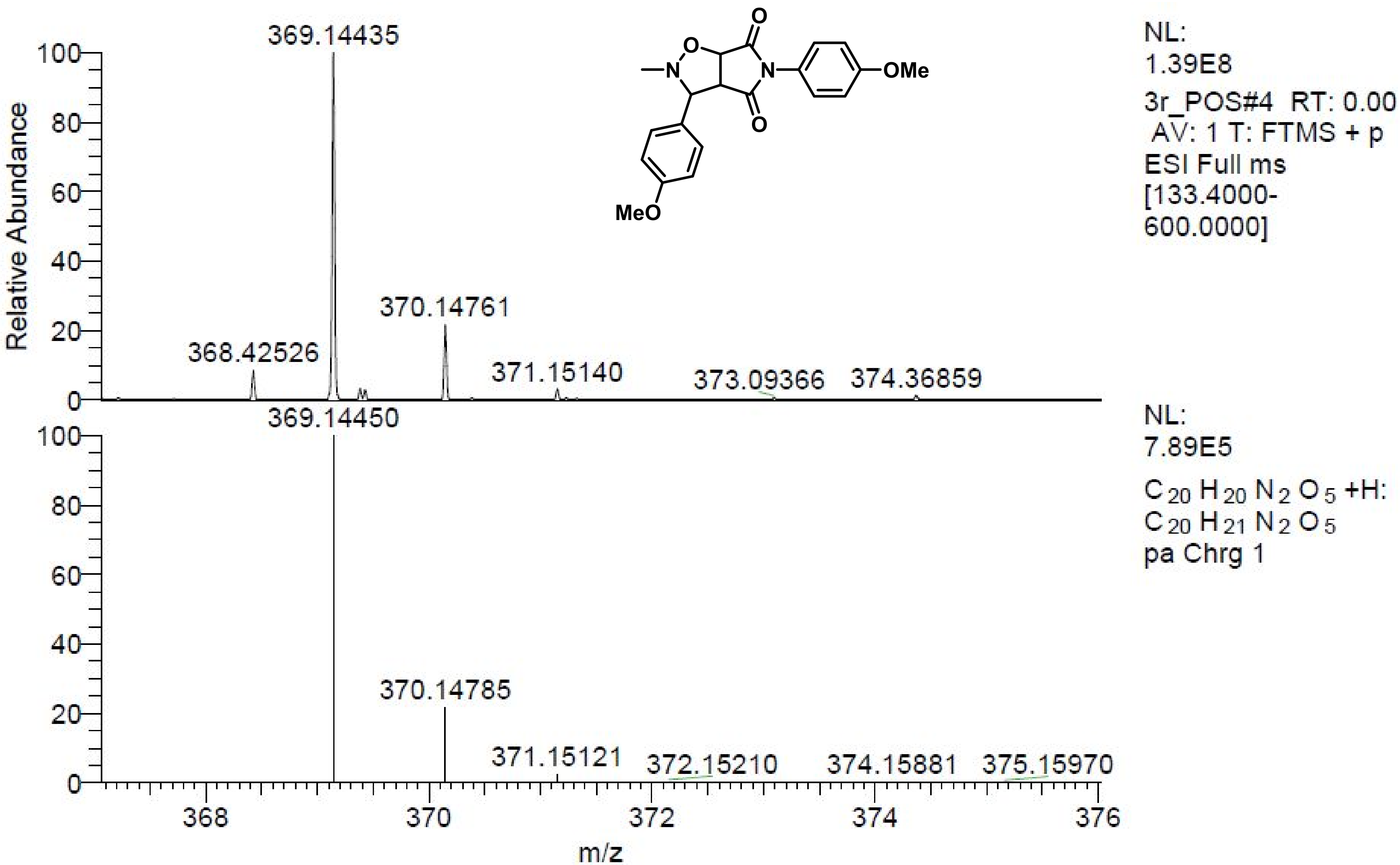


**Figure S76.** HRMS spectrum of **3q** ***(cis isomer)*.**

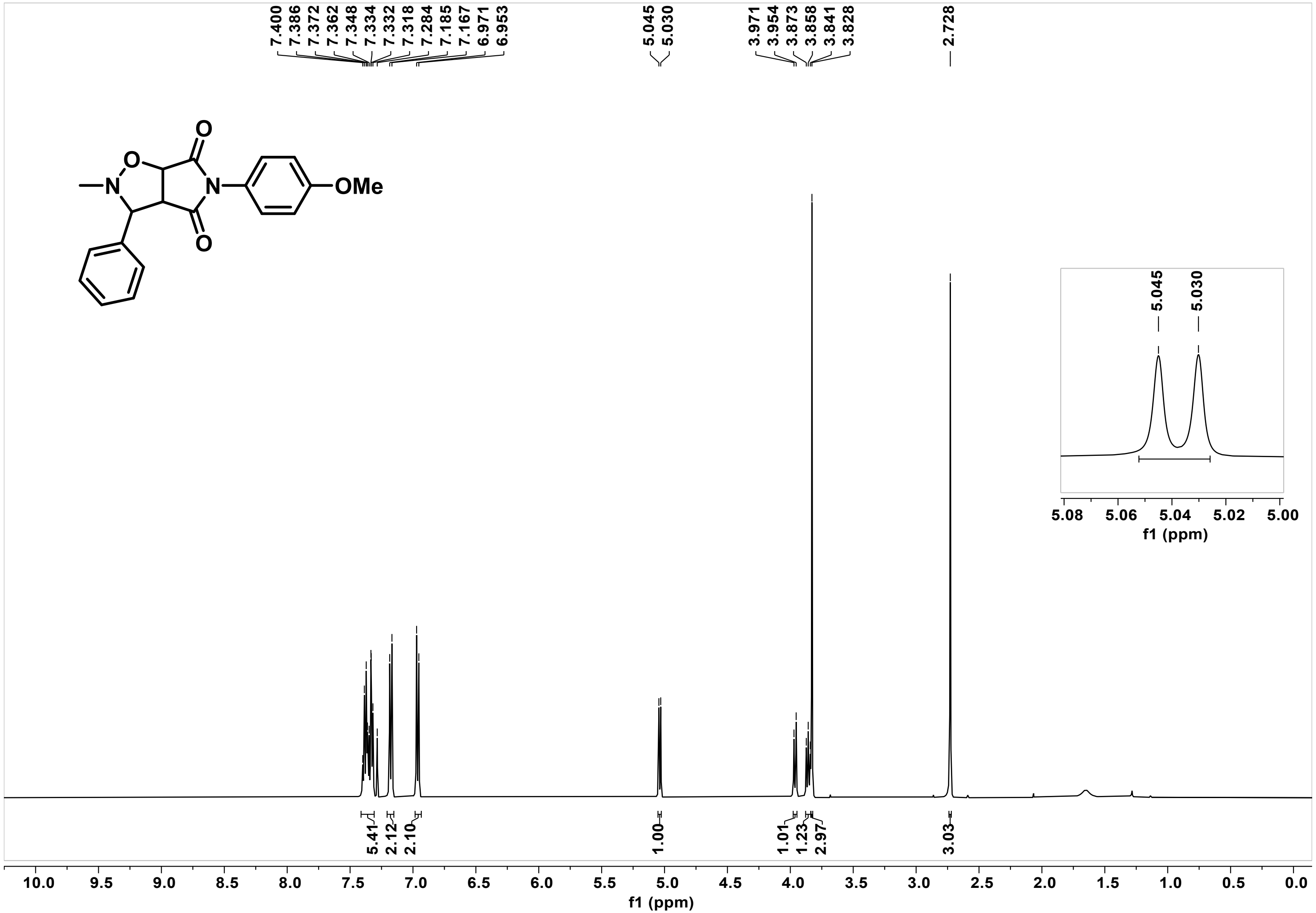


**Figure S77**. $^{1}$H NMR spectrum of **3r** ***(cis isomer)*** (500 MHz, $CDCl_3$).

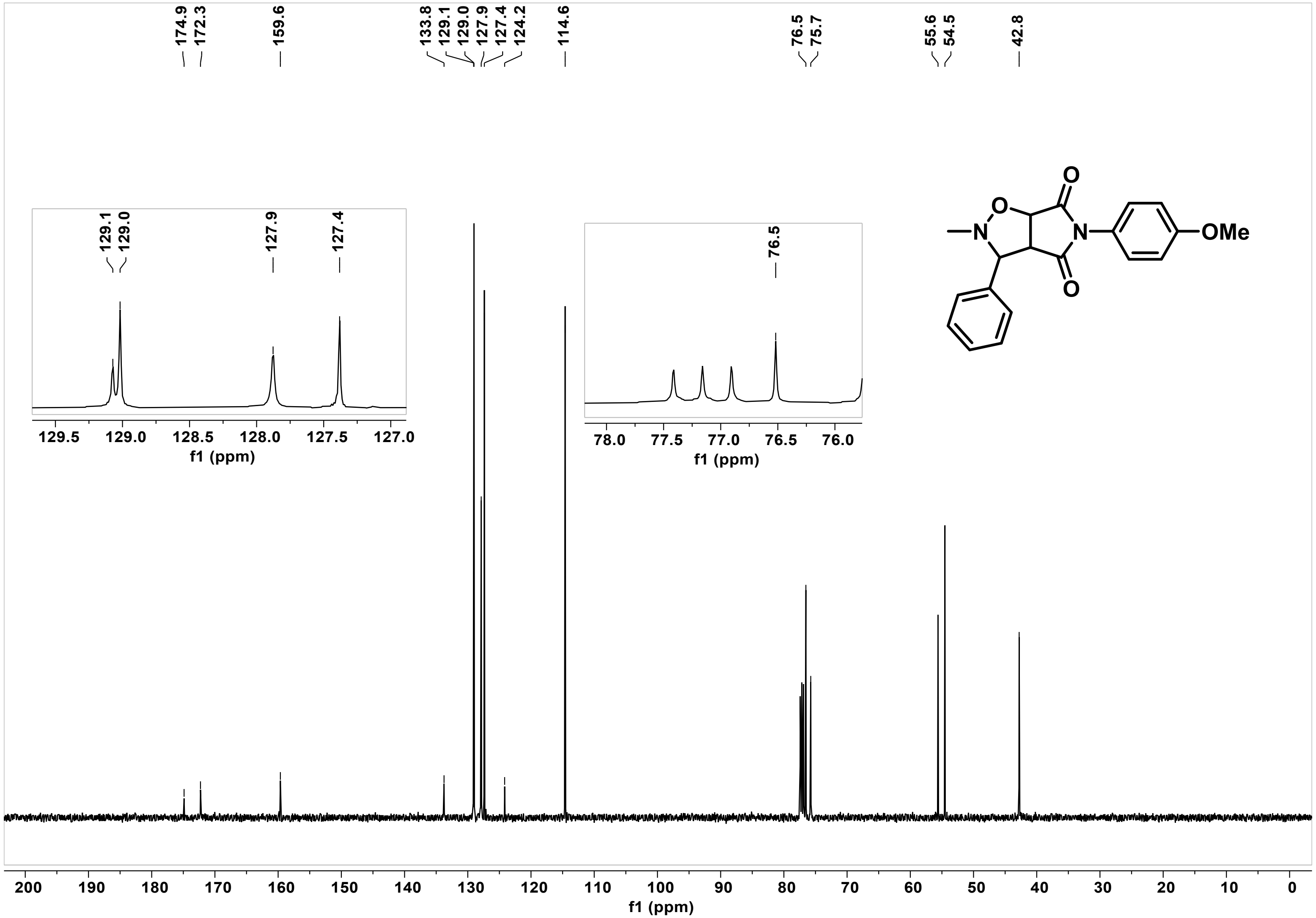


**Figure S78.** $^{13}$C NMR spectrum of **3r** ***(cis isomer)*** (125 MHz, $CDCl_3$).

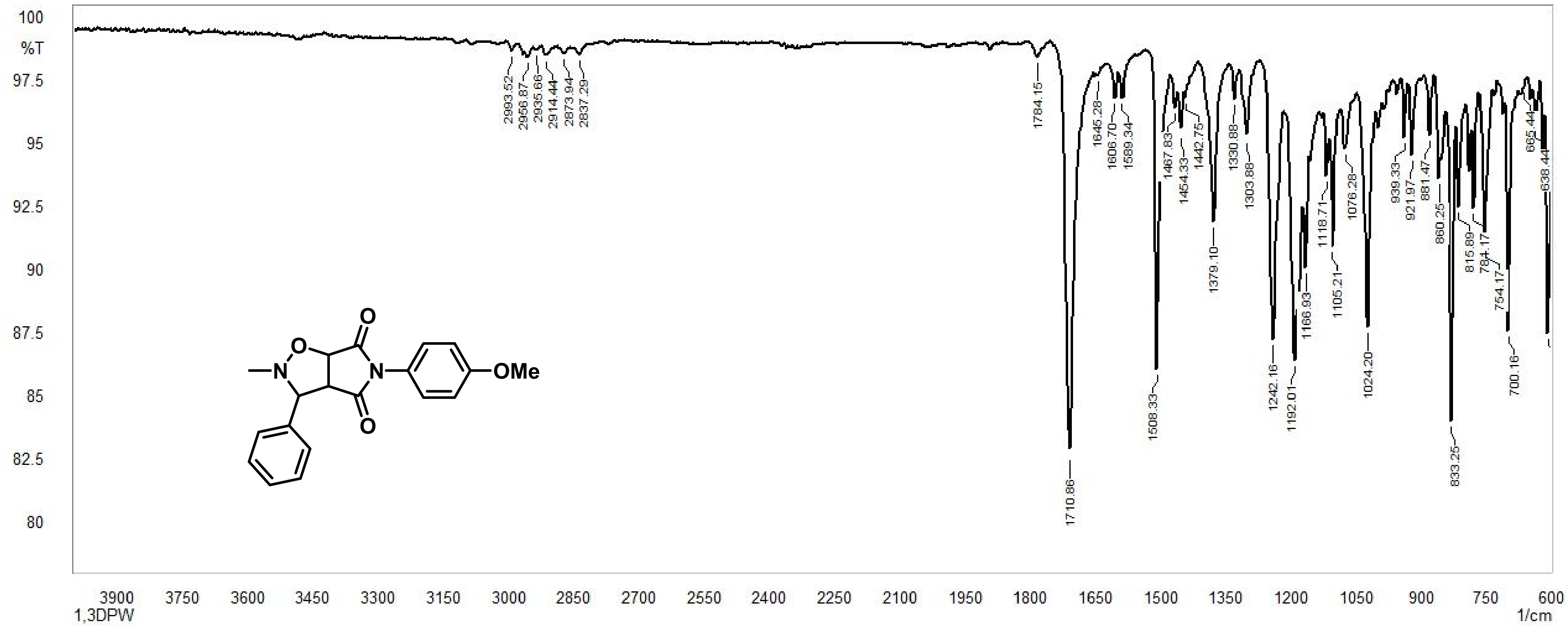


**Figure S79.** FT-IR spectrum of **3r** ***(cis isomer)*****.**

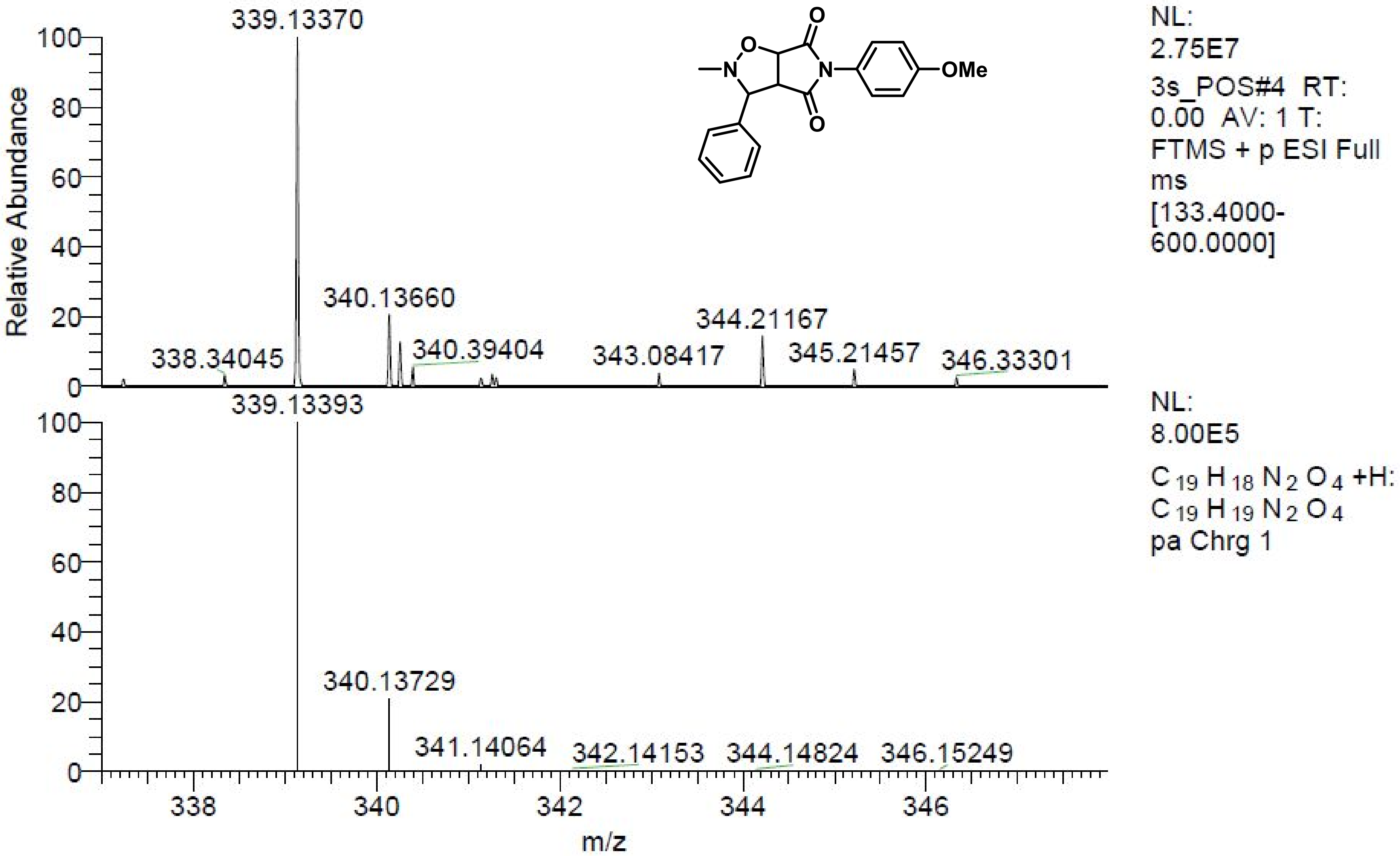


**Figure S80.** HRMS spectrum of **3r** ***(cis isomer)*.**

## 5. Scale-up experiments and Green Chemistry Metrics

***Procedure to scaled mechanochemical synthesis of 3-(4-methoxyphenyl)-2,5-diphenyltetrahydro-4H-pyrrolo[3,4-d]isoxazole-4,6(5H)-dione* 3k.**[45]

In the 12 mL stainless steel container of the ball mill, the nitrone **1a**, the maleimide **2b** and 10 stainless steel spheres (5 mm diameter) were added. The reactor was placed in a planetary ball mill, and the parameters were adjusted to a rotation speed of 550 rpm, with directional inversion every 30 minutes. When necessary, silica ($SiO_2$) was added as a milling auxiliary (indicated in each case). After grinding, the crude mixture was dissolved in ethyl acetate (AcOEt) and transferred to a 25 mL round-bottom flask, followed by concentration under reduced pressure. The crude product was then recrystallized from hexane/ethyl acetate to afford a white solid. In cases where silica ($SiO_2$) was used as a milling auxiliary, the reaction mixture was dissolved in ethyl acetate (AcOEt) and transferred to a sintered-glass funnel. The silica was washed twice with hot AcOEt to ensure maximal product recovery. The combined filtrates were concentrated under reduced pressure, and the resulting residue was recrystallized as described above.

**3k(a). 1a** = 228 mg (1 mmol), **2b** = 173 mg (1 mmol). Reaction time - **1** hour. Purified by recrystallization in AcOEt/hexane. White solid, (168 mg – 42 % yield -*cis* isomer), mp: 195–197 °C (Lit.[44] 193–195 °C). **3k(b). 1a** = 228 mg (1 mmol), **2b** = 173 mg (1 mmol). Silica = 400 mg Reaction time - **1** hour. Purified by recrystallization in AcOEt/hexane. White solid, (196.8 mg – 49.2 % yield -*cis* isomer), mp: 196–198 °C (Lit.[44] 193–195 °C).

**3k(c). 1a** = 228 mg (1 mmol), **2b** = 173 mg (1 mmol). Reaction time - **2** hours. Purified by recrystallization in AcOEt/hexane. White solid, (316 mg – 79 % yield -*cis* isomer), mp: 195–197 °C (Lit.[44] 193–195 °C). **3k(c). 1a** = 2.28 g (10 mmol), **2b** = 1.73 g (10 mmol). Silica = 2 g. 50 mL stainless steel container. 20 stainless steel spheres (5 mm diameter). Reaction time - 16 hours. Purified by recrystallization in AcOEt/hexane. White solid, (2.61 g – 65 % yield -*cis* isomer), mp: 195–197 °C (Lit.[44] 193–195 °C).

**Table S1.** Green Chemistry Metrics.

| Product | E factor | RME[a] | Mass Intensity | Mass Productivity | Time (h) |
|---|---|---|---|---|---|
| **3a** | 72,30 | 92 | 1,09 | 91,89 | 1 |
| **3b** | 45,10 | 87 | 1,16 | 86,53 | 1 |
| **3c** | 37,98 | 96 | 1,06 | 94,48 | 1 |
| **3d** | 37,90 | 94 | 1,06 | 94,11 | 1 |
| **3e** | 82,37 | 89 | 1,66 | 60,29 | 2 |
| **3f** | 56,91 | 87 | 1,14 | 88,05 | 1 |
| **3g** | 35,29 | 92 | 1,09 | 91,54 | 3 |
| **3h** | 77,09 | 87 | 1,15 | 86,66 | 1 |
| **3i** | 117,16 | 34 | 2,84 | 35,26 | 2 |
| **3j** | 183,36 | 41 | 2,48 | 40,38 | 3 |
| **3k** | 89,86 | 81 | 1,24 | 80,37 | 1 |
| **3l** | 33,88 | 92 | 1,05 | 95,17 | 2 |
| **3m** | 55,94 | 70 | 1,47 | 67,86 | 3 |
| | | | Scaled experiments | | |
| **3k(a)** | 80,54 | 42 | 2,39 | 41,89 | 1 |
| **3k(b)** | 69,26 | 49,2 | 2,04 | 49,08 | 1 |
| **3k(c)** | 42,82 | 79 | 1,27 | 78,80 | 2 |
| **3k(d)** | 27,75 | 65 | 1,54 | 64,84 | 16 |
| | | | Reference | | |
| Ref.[44] | 104,34 | 100 | 1,03 | 97,37 | Stirring |
| Ref.[46] | 300,98 | 58 | 1,70 | 58,91 | Reflux |
| Ref.[46] | 302,11 | 54 | 1,83 | 54,76 | Reflux |

[a] Reaction Mass Efficiency. [b]The metrics were calculated by this team based on data from a referenced article.

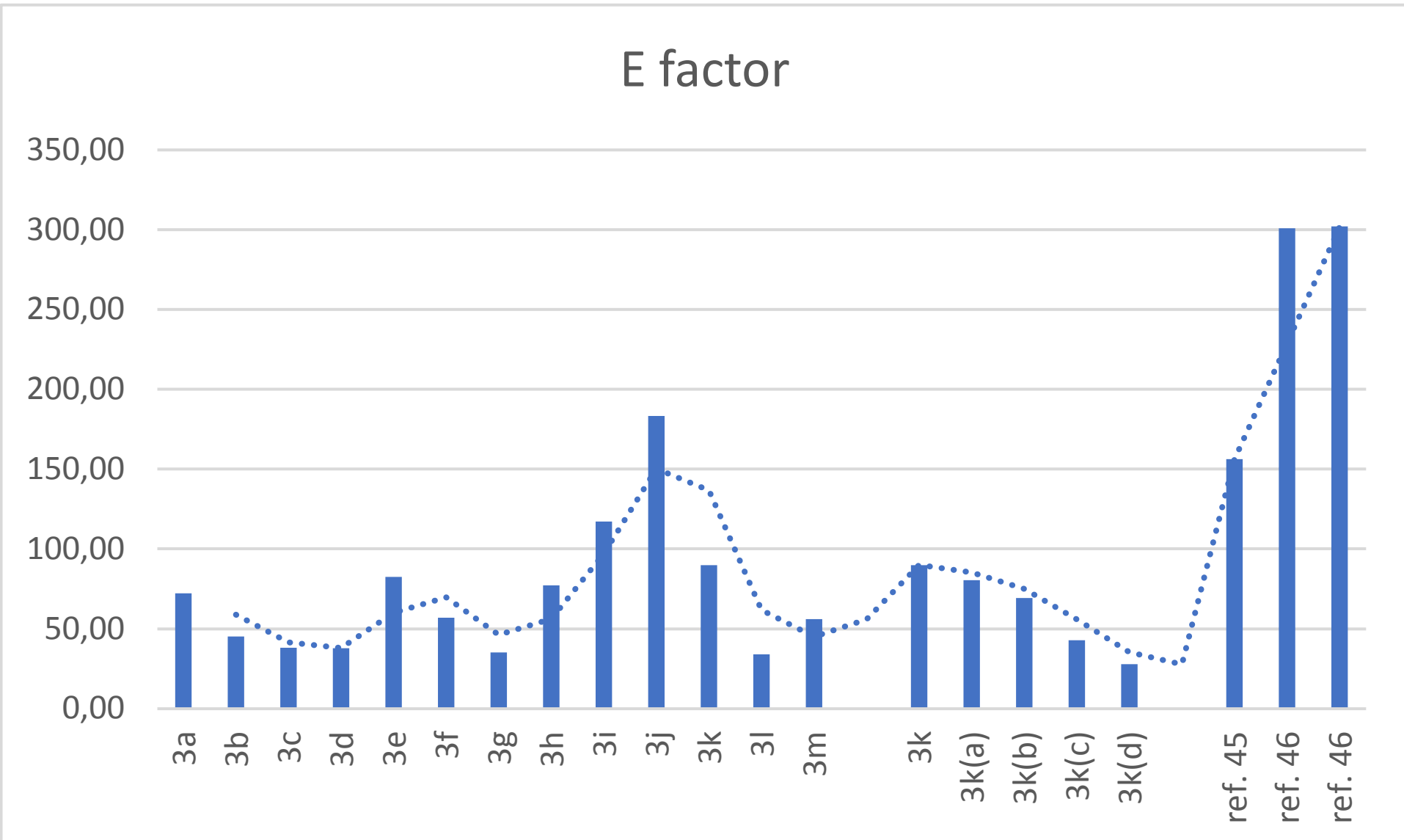


**Figure S81.** Green Chemistry Metrics: E factor.

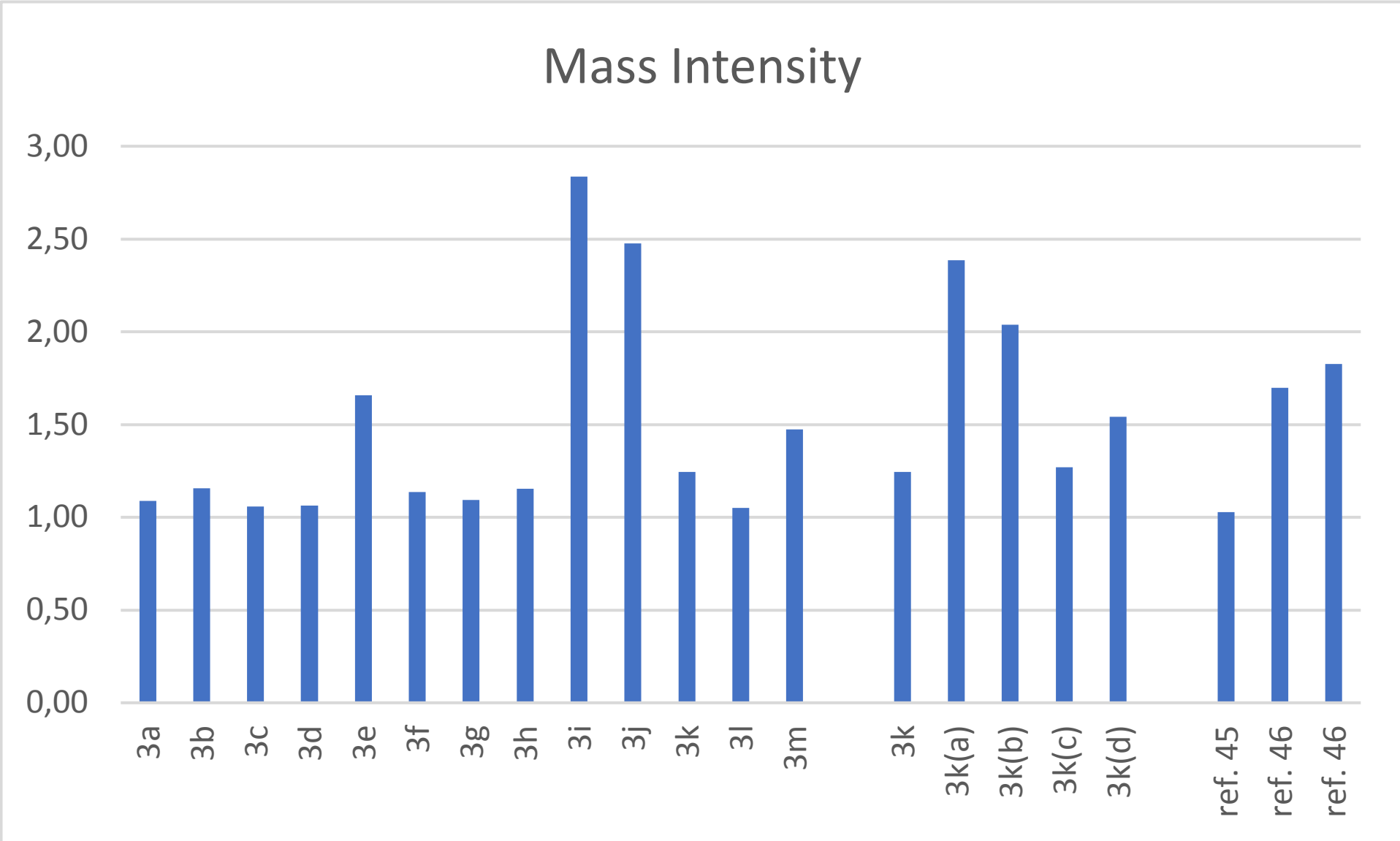


**Figure S82.** Green Chemistry Metrics: Mass Intensity.

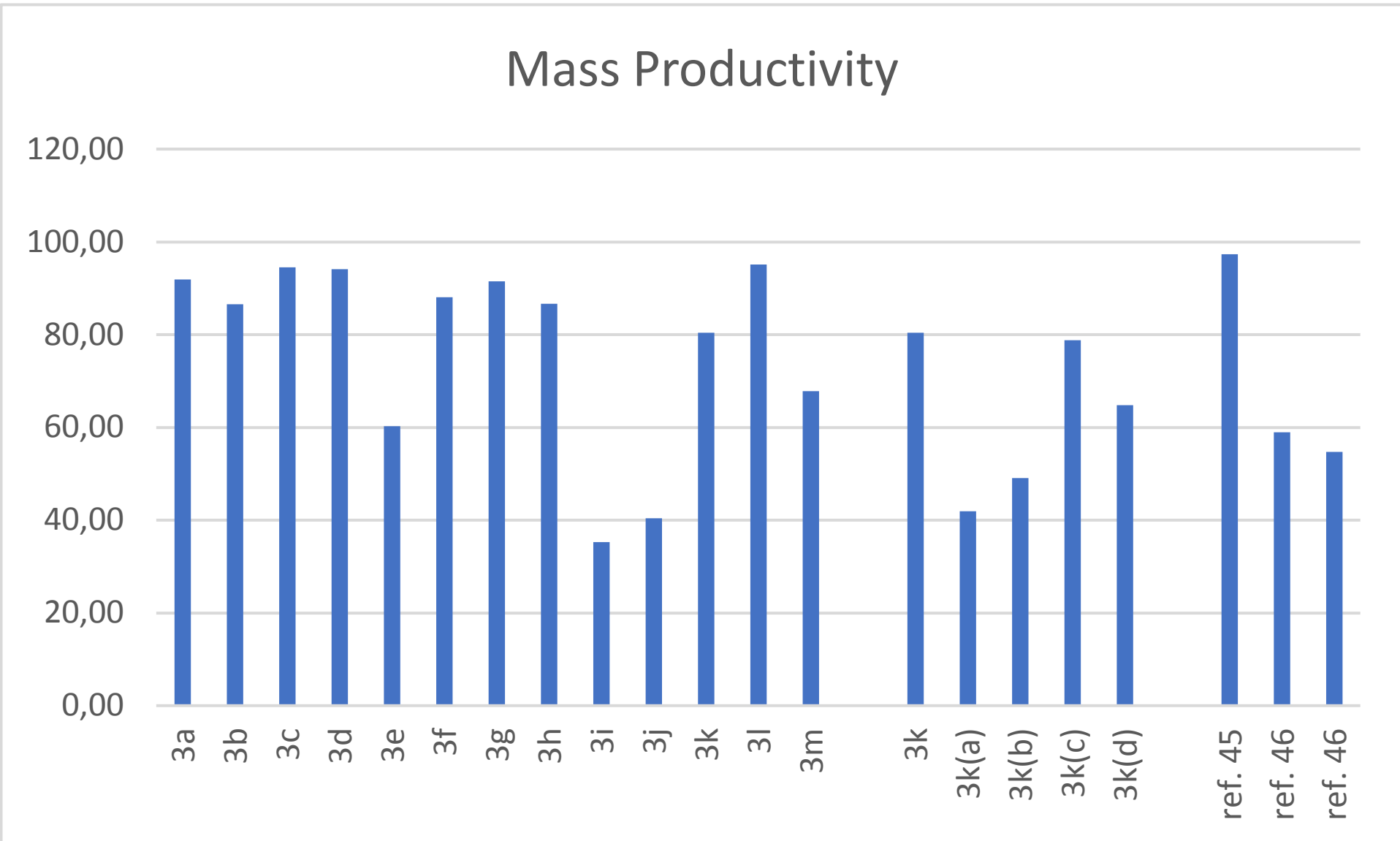


**Figure S83.** Green Chemistry Metrics: Mass Productivity.

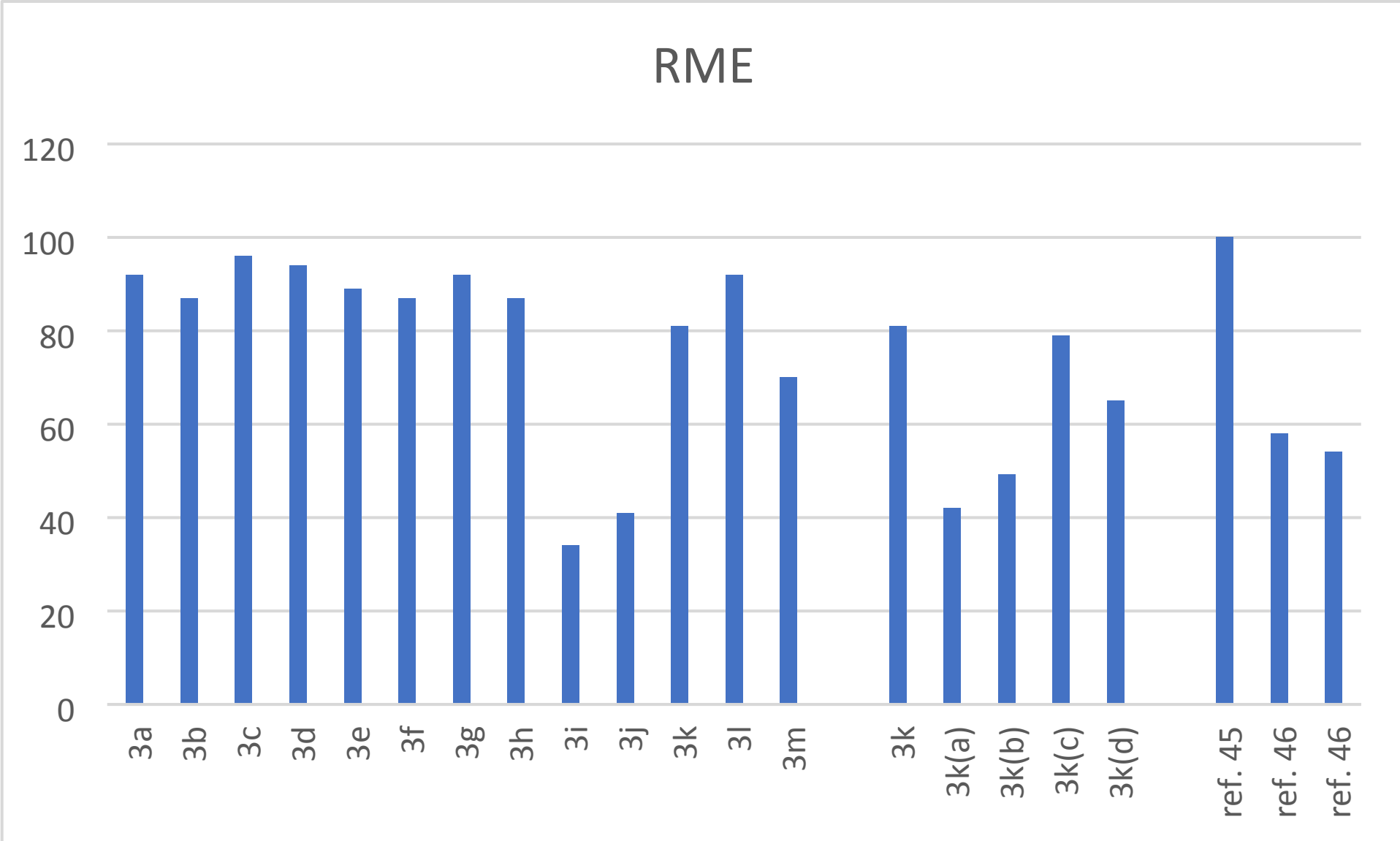


**Figure S84.** Green Chemistry Metrics: Reaction Mass Eficiency (RME).
